# Construction of a hierarchical linear regression prediction model for classical Chinese poetry writing ability development

**DOI:** 10.1371/journal.pone.0361497

**Published:** 2026-10-30

**Authors:** Peigen Shi, Lei Zheng

**Affiliations:** 1 School of Teacher Education, Heze University, Heze, Shandong, China; 2 Basic Teaching Department, Heze Technician College, Heze, Shandong, China; Bahir Dar University, ETHIOPIA

## Abstract

Although classical poetry is central to Chinese language education, how poetry learning is linked to writing development has rarely been characterized empirically. This one-year longitudinal study followed 1,920 students in Grades 3–6 from 48 classes in 12 primary schools across four waves and applied a four-level growth model (measurement occasions nested within students, students within classes, and classes within schools). The cognitive load components were indexed provisionally by NASA-TLX subscales, and results that depend on their distinction are interpreted as preliminary. Eight confirmatory tests were controlled with the Holm procedure and all remaining analyses were reported as exploratory with Benjamini-Hochberg control; a random-intercept cross-lagged panel model examined within-person lagged associations, which inform rather than establish the ordering of the modeled associations; and the 12-school highest level was examined with leave-one-school-out refits, a wild cluster bootstrap, prior sensitivity analyses, a school fixed-effects model, and a calibrated Monte Carlo simulation. Intrinsic (β = −0.41) and extraneous (β = −0.49) cognitive load were negatively and germane cognitive load (β = 0.45) positively associated with writing ability (Holm-adjusted p < .001), and schema construction, imagery processing, and emotional resonance carried statistical indirect associations totaling 0.587 (76.2% of the total association). The interaction of teaching method with cognitive load (γ = 0.47, Holm-adjusted p = .011) was retained under all small-cluster sensitivity analyses, whereas the interactions with grade and class environment were exploratory and the school-level variance component was imprecisely estimated, and writing ability followed a mildly decelerating quadratic trajectory ($\pi_{\text{2}}$ = −0.17 points per wave², Holm-adjusted p < .001). With imputation, centering, model reselection, refitting, and BLUP computation confined to the training folds, prediction for new students in the sampled classes and schools reached $R^{\text{2}}$ = 0.847 and RMSE = 4.28, meeting the preset RMSE target, whereas three-band accuracy (80.3%) fell short of the preset 85% target, and prediction for unseen classes and schools reached $R^{\text{2}}$ = 0.768 and 0.656. The findings characterize the multilevel associations between classical poetry learning and writing development and support instructional recommendations restricted to the confirmatory results.

## 1. Introduction

Classical poetry, a signature vehicle of Chinese culture, occupies a central place in Chinese language education. Yet the longitudinal association between students’ cognitive load during poetry learning and the development of their writing ability remains poorly characterized, and systematic evidence on how cognitive resources are distributed across grade levels and how skills transfer to writing is still lacking.

Recent progress in cognitive load theory frames the present investigation. Sweller et al. emphasized the dynamic interaction among intrinsic, extraneous, and germane load and the moderating role of learner and environmental factors [1], and Sweller argued that long-term-memory content shaped by environmental exposure is the principal source of individual variation [2].

Empirical work links classical-poetry processing to language outcomes: interactive Tang-poetry instruction improved motivation and comprehension [3], schema-associative training raised poetry retention by 131% [4], and emotion-congruent multimedia design added further benefits [5]. Component skills and higher-order cognitive resources jointly determine writing quality [6], writing trajectories differ across learners [7], working-memory updating predicts children’s writing [8], working memory underpins literacy development [9,10], reading transfers cumulatively to writing [11], and hierarchical linear modeling is the standard for nested educational data [12,13].

A critical gap remains in understanding how the cognitive load of classical poetry learning is longitudinally linked to writing ability across grades, in particular: (1) the differentiated associations of the cognitive load subcomponents; (2) the mediating role of schema construction, imagery processing, and emotional resonance; (3) cross-level moderation by instructional context; and (4) the nonlinear growth trajectory of writing ability.

The present study addresses these gaps with a multilevel longitudinal predictive model of the cognitive-load-writing pathway (Fig 1). Students in Grades 3–6 (1,920 students in 48 classes across 12 primary schools) were followed for one year, a four-level growth model separated occasion, student, class, and school effects, and a supplementary random-intercept cross-lagged panel analysis (Section 4.3) was used to examine within-person lagged associations, which inform, but cannot establish, the ordering of the modeled associations. The three cognitive load types were indexed provisionally by NASA-TLX subscales. Predictive performance is evaluated primarily for new students within the sampled classes and schools, and its transfer to unseen classes and schools is assessed with leave-one-class-out and leave-one-school-out validation, with imputation, preprocessing, model fitting, and random-effect prediction performed within the training folds. The analyses separate a small set of confirmatory hypotheses, tested with familywise error control, from exploratory analyses, and the stability of the school-level estimates is examined explicitly because the highest level comprises only 12 schools.

**Fig 1:**
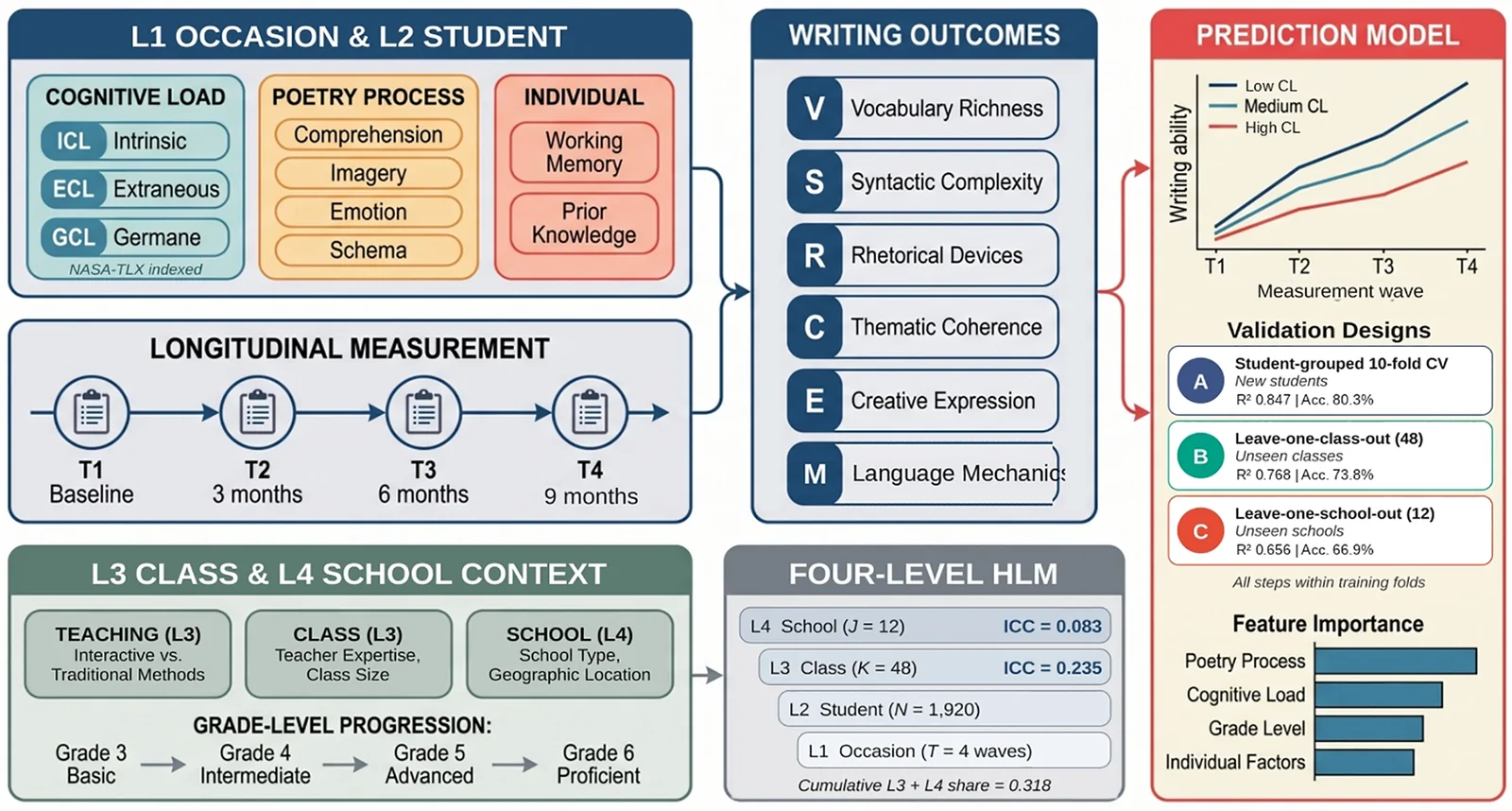
Research framework. A four-level HLM of the classical poetry cognitive load and writing development association, with student-grouped, leave-one-class-out, and leave-one-school-out validation.

## 2. Literature review and theoretical framework

### 2.1. Application of cognitive load theory in language education

Cognitive load theory distinguishes task complexity (intrinsic), ineffective instruction (extraneous) [1], and schema construction (germane) together with available resources and element interactivity, treats cognitive load as a multidimensional construct spanning mental load, mental effort, and performance [14], and still requires clearer measurement of the tripartite distinction [15]. Visual-analog and Likert scales [16], multi-source evidence [17], and physiological indices from EEG, eye tracking [18], and fNIRS [19] have been proposed, none of which maps one-to-one onto the load types.

### 2.2. Classical poetry learning and cognitive processing

Recent work has begun to expose the cognitive mechanisms of Chinese poetry learning. An interactive Tang-poetry application raised motivation (Cohen’s d = 1.15) and comprehension (d = 0.63) in Grade 3 learners while risking cognitive overload [3], schema-associative memory training raised poetry retention by 131% in 127 pupils [4], and emotion-congruent multimedia design improved retention and transfer (n = 166) [5]. Immersive environments are an instructive counter-case: a virtual-reality poetry intervention increased engagement yet impaired memory retention [20], a finding revisited in Section 5.2, and evidence from VR-based language learning [21] and Chinese instructional settings [22] shows that these mechanisms are general but culture-sensitive.

The framework presupposes that late-childhood learners can differentiate the three load components in self-reports. Metacognitive monitoring and executive function mature substantially between ages 7 and 12 [23], and cognitive load theory has been refined conceptually and operationally [24]. The Klepsch et al. questionnaire differentiates intrinsic, extraneous, and germane load [25] but was validated with learners older than the present participants, whereas NASA-TLX was developed as a general workload inventory; germane load is now treated as the working-memory resources devoted to intrinsic load rather than as an independent additive load [1], and the tripartite distinction lacks unambiguous measurement [15]. The mapping of NASA-TLX subscales onto the three load types is therefore treated as a provisional operationalization whose assumptions are stated in Section 3.3; items were anchored to the preceding poetry task in age-appropriate language, a convergent validation with an adapted Chinese subset of the Klepsch et al. instrument was conducted in a 20% subsample (Section 4.6), and results that depend on the three load labels are interpreted with caution.

### 2.3. Writing ability development and assessment

Writing-development research treats writing ability as multidimensional: component skills and higher-order cognitive resources jointly determine writing quality [6], children follow steady, accelerated, or plateau trajectories [7], strategy instruction outperforms grammar instruction (ES = 0.96 versus 0.11) [26], and metacognitive and explicit writing instruction raise writing quality [27,28]. Writing ability is therefore modeled with six outcomes (vocabulary, syntax, rhetoric, coherence, creativity, mechanics).

### 2.4. Hierarchical linear models in educational research

Hierarchical linear modeling is the de facto standard for nested educational data [12,29]. In a Monte Carlo comparison, omitting existing random effects inflated Type I error rates, whereas redundant random effects caused convergence problems under REML and losses of power and of accuracy in $R^{\text{2}}$ measures under Bayesian estimation [13].

These findings indicate that the choice of estimator does not remove the uncertainty attached to random-effects parameters, which is greatest at the highest level of a design with few units. In the present design the highest level comprises 12 schools, so the stability of the school-level variance component and of school-level and cross-level coefficients has to be examined in the data themselves. The study therefore uses REML for the main four-level model, reports how Bayesian estimates depend on the prior, and subjects the principal Level-4 and cross-level estimates to dedicated small-cluster sensitivity analyses (Sections 3.5 and 4.6.5).

### 2.5. Theoretical model construction and research hypotheses

Integrating cognitive load theory, writing-development research, and Chinese poetry education, this study proposes a multilevel model built around six components (Fig 2). Individual-level predictors comprise intrinsic (ICL), extraneous (ECL), and germane (GCL) cognitive load, indexed by NASA-TLX subscales under the provisional mapping described in Section 3.3, together with working-memory capacity and prior knowledge.

**Fig 2:**
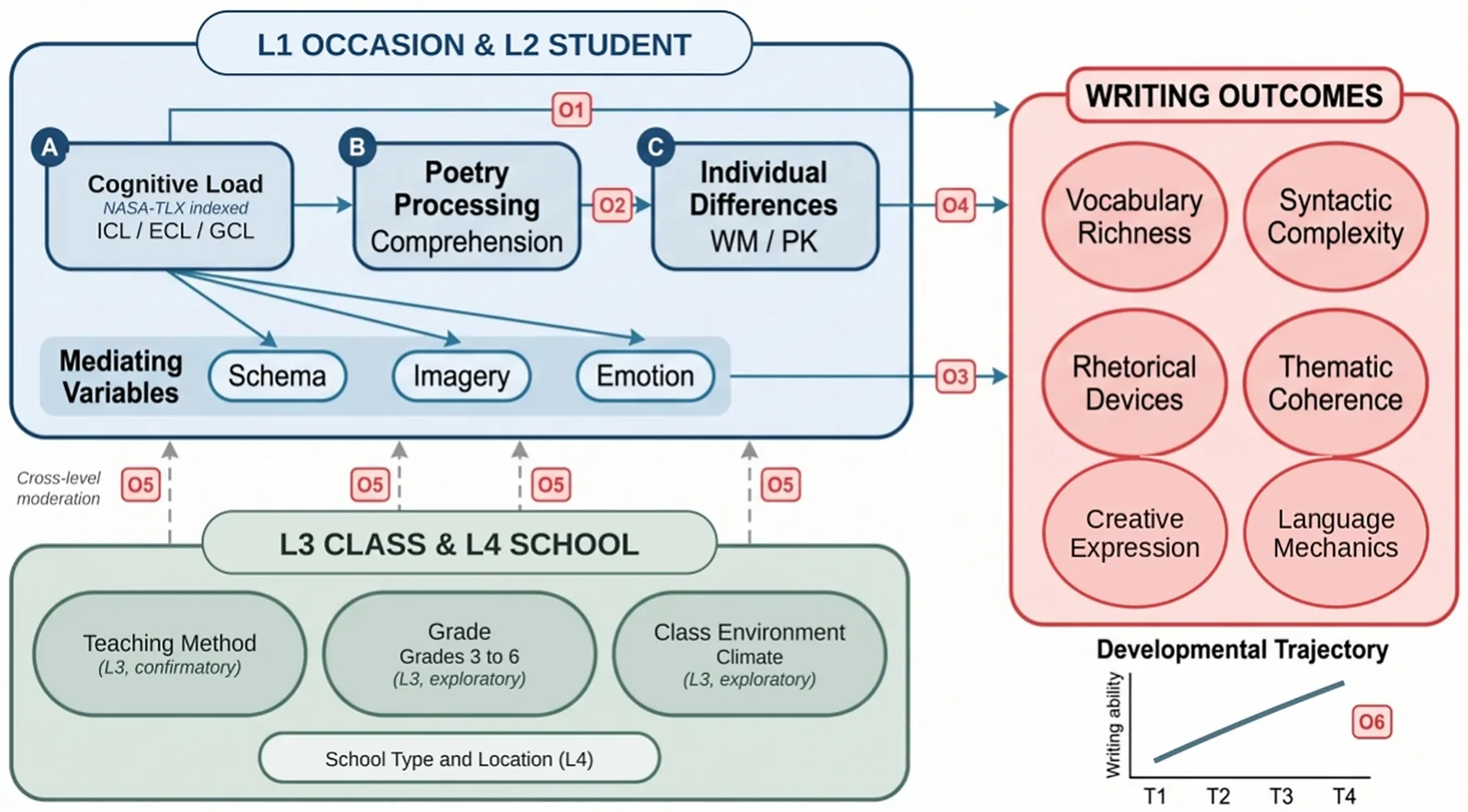
Theoretical model of poetry cognitive load and writing development within the four-level nesting structure (measurement occasion nested within student, student within class, class within school). Class-level (teaching method, teacher expertise, class size) and school-level (school type, geographic location) variables enter the model as Level-3 and Level-4 predictors of intercepts and as cross-level moderators of Level-1 slopes.

Schema construction, imagery processing, and emotional resonance are modeled as parallel but correlated mediating pathways; indirect effects are estimated by the coefficient-product method and summarized via the total mediation proportion $P_{M}$. Because the predictors, mediators, and outcome are observed rather than manipulated, the indirect effects are interpreted as statistical indirect associations under the hypothesized ordering rather than as demonstrated causal mechanisms.

Temporal growth is modeled as a quadratic function of measurement occasion, $f\left(t\right)=\pi_{\text{0}}+\pi_{\text{1}}t+\pi_{\text{2}}t^{\text{2}}$, with random intercept $\pi_{\text{0}}$, random linear slope $\pi_{\text{1}}$, and a fixed quadratic term $\pi_{\text{2}}$.

The analytic task is to estimate the hypothesized mediation and moderation associations and predict writing ability for new students within the sampled classes and schools, while assessing how far these predictions transfer to unseen classes and schools. Six modeling objectives (O1 to O6) with quantitative acceptance criteria are defined below:

O1 (cognitive-load response representation): quantify the differential response of writing ability to the three TLX-indexed cognitive-load components; acceptance threshold |β| ≥ 0.20 with 95% CI excluding zero for ICL, ECL, and GCL.

O2 (coupling of poetry processing and cognitive resources): estimate the standardized coupling between poetry comprehension processing and working-memory capacity and prior-knowledge activation; acceptance criteria include incremental R² and p < 0.01.

O3 (mediation path estimation): estimate the indirect associations along the three mediation pathways from cognitive load to writing ability, reporting each indirect effect ab with its 95% Monte-Carlo confidence interval, the mediation proportion $P_{M}=ab/c$, where c is the total association, and Sobel Z statistics.

O4 (individual-feature contribution): regress the six writing sub-dimensions on working-memory capacity and prior knowledge, reporting partial coefficients, partial R², and importance scores.

O5 (cross-level moderation): estimate the cross-level interaction coefficient γ of class-level teaching context on individual-level cognitive-processing paths, with the simple-slope contrast between interactive and traditional instruction; the interaction of cognitive load with teaching method is the confirmatory test, whereas its interactions with grade and class environment are exploratory extensions.

O6 (longitudinal prediction): fit the quadratic growth model with individual-, class-, and school-level predictors, reporting the quadratic term, class-level and school-level ICCs with 95% profile-likelihood intervals, AIC/BIC, and accuracy (target ≥ 85%), RMSE (target ≤ 5.0), ROC-AUC, and variable importance under student-grouped 10-fold cross-validation for new students within the sampled classes and schools; performance for unseen classes and schools is reported without acceptance thresholds (Section 3.6).

The six objectives differ in inferential status. Four hypotheses that follow directly from the theoretical model are designated as confirmatory: the associations of the three TLX-indexed load components with writing ability (O1), the three indirect effects (O3), the interaction of cognitive load with teaching method (O5), and the quadratic growth term (O6). These eight tests form one family whose p values are adjusted with the Holm procedure, and predictive performance for new students within the sampled classes and schools serves as the confirmatory predictive criterion of O6. All remaining analyses are exploratory under Benjamini-Hochberg control and generate hypotheses rather than support conclusions or instructional recommendations.

## 3. Research methods

### 3.1. Research design

This study uses a longitudinal design with multistage stratified sampling and four measurement waves (Fig 3). After a three-month literature review, the measurement tools were piloted with 120 students, 48 classes across 12 primary schools spanning Grades 3–6 were sampled (Section 3.2), and four assessments were administered across one academic year (start of Year, end of first semester, middle of second semester, end of Year). Because all participants were minors aged approximately 8–12 years, written informed consent was obtained from the legal guardians of every participating student before data collection, together with age-appropriate written assent from each student. Participation was voluntary, data were de-identified, withdrawal was possible at any time without academic consequence, and the consent and assent forms are archived in the project GitHub repository.

**Fig 3:**
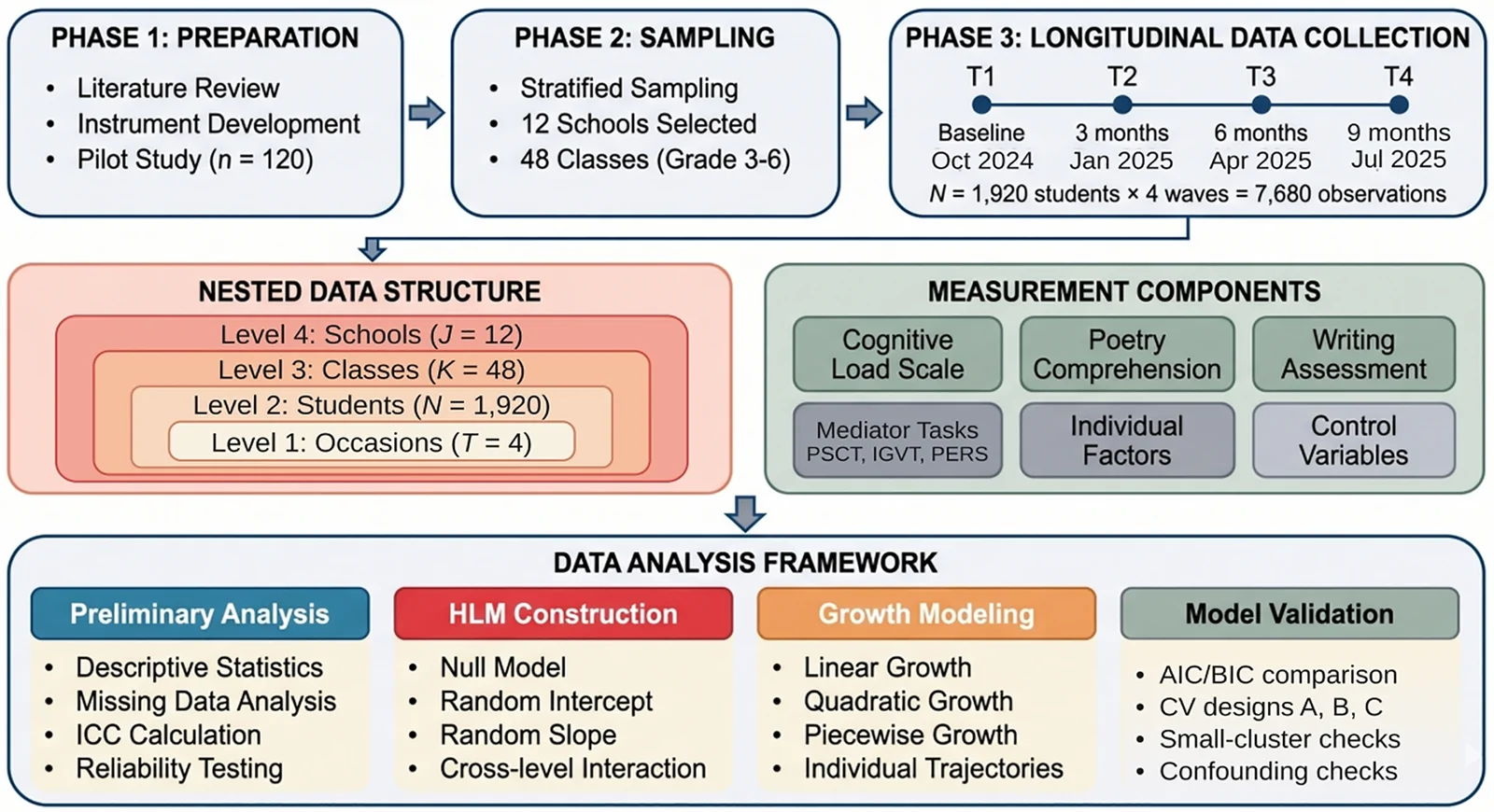
Longitudinal research design framework. (four measurement waves (T1 to T4) nested within students, students within classes (K = 48), and classes within schools (J = 12), yielding N = 1,920 students and 7,680 person-wave observations in a four-level hierarchical structure).

### 3.2. Research participants and sampling

The selection of participants followed a multistage stratified random sampling procedure. Primary schools in the province were classified into three composite tiers by teaching quality, geographic location, and school size, and four schools were randomly selected from each tier and distributed across urban, suburban, and rural locations, so that the quality tier remains orthogonal to the location covariate of the four-level model. The 12 schools (4 urban, 3 suburban, and 5 rural) reproduce the student-level distribution in Table 1, and the cross-tabulation for all 12 schools is provided in Supplemental Table S1 in S1 File. One class was randomly selected from each of Grades 3–6 within each school, and 40 students whose guardians gave consent were randomly drawn from each selected class, yielding 1,920 students in 48 classes. Because the highest level of the design comprises only 12 schools, the precision and stability of the school-level variance component and of school-level and cross-level coefficients were examined with dedicated small-cluster sensitivity analyses (Sections 3.5 and 4.6.5). This study began on August 15, 2024, and ended on August 23, 2025, and followed the 1964 Declaration of Helsinki and its later amendments. This study was reviewed and approved by the Research Ethics Committee of Heze University Teacher Education College, and written informed consent was obtained from the legal guardians of all participating minors, in addition to age-appropriate assent from the students themselves. The sample distribution characteristics are shown in Fig 4.

**Table 1:** Sample basic characteristic description.

Variable	Category	Grade 3	Grade 4	Grade 5	Grade 6	Total
Sample Size	n	480	480	480	480	1920
Gender	Male	250(52.1%)	245(51.0%)	240(50.0%)	254(52.9%)	989(51.5%)
	Female	230(47.9%)	235(49.0%)	240(50.0%)	226(47.1%)	931(48.5%)
School Location	Urban	160(33.3%)	160(33.3%)	160(33.3%)	160(33.3%)	640(33.3%)
	Suburban	120(25.0%)	120(25.0%)	120(25.0%)	120(25.0%)	480(25.0%)
	Rural	200(41.7%)	200(41.7%)	200(41.7%)	200(41.7%)	800(41.7%)
Enrolled class size	Mean(SD)	44.9(2.8)	44.6(2.5)	44.3(2.3)	44.0(2.1)	44.5(2.4)
	Range	41-51	41-50	41-49	41-48	41-51

**Fig 4:**
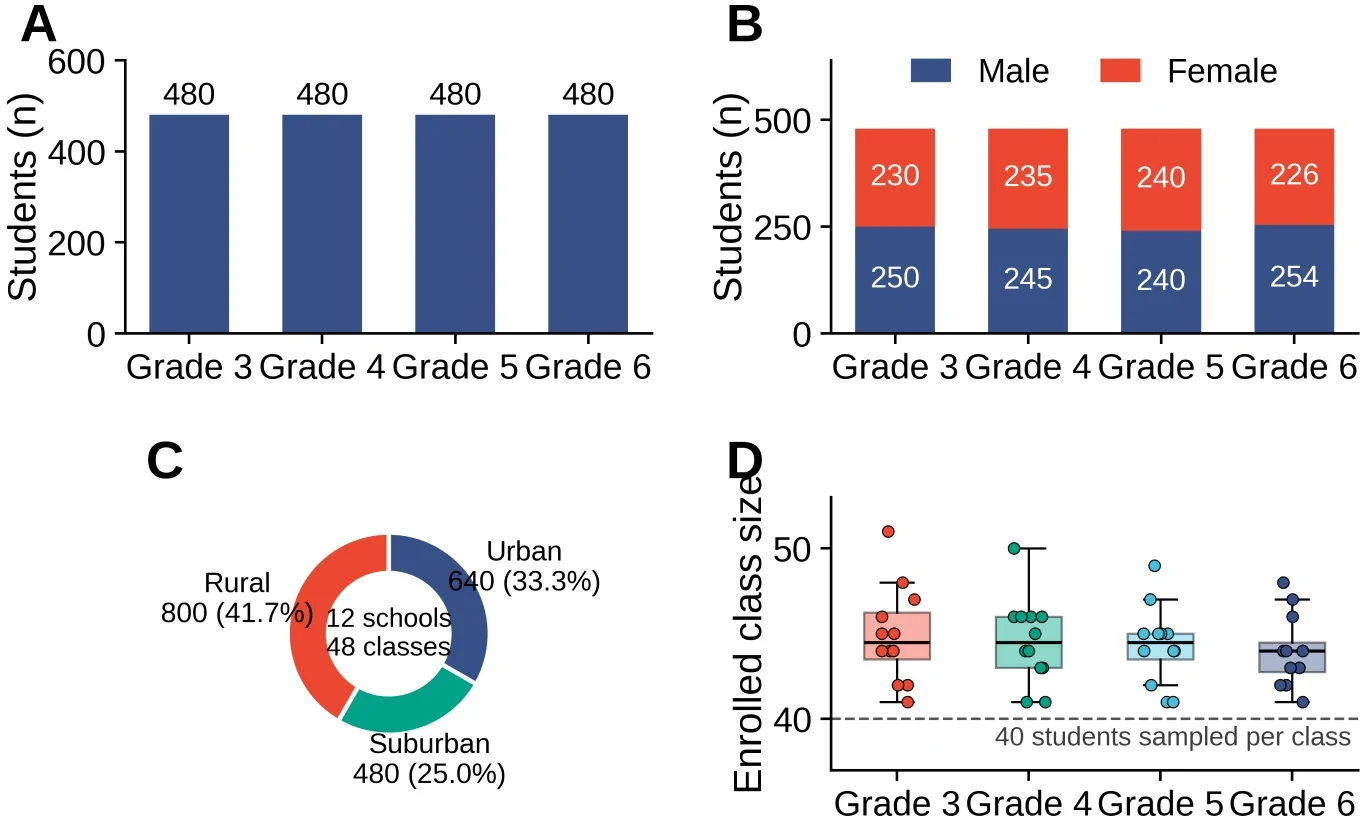
Sample distribution and characteristics.

The sample is balanced in grade (480 students in 12 classes per grade) and gender (male–female ratio close to 1:1, within 3 percentage points across grades), spans urban (33.3%), suburban (25.0%), and rural (41.7%) schools, and has stable enrolled class sizes (medians of 44–45 students; Table 1), from each of which 40 students were sampled.

### 3.3. Measurement tool development and validation

The measurement tools were developed in four stages (theoretical construction, expert review, pilot-based item refinement, and formal administration), with pilot testing on 120 pupils not in the main sample. Cognitive load was measured using an adapted three-subscale NASA-TLX instrument (Mental Effort, Task Difficulty, Performance/Self-efficacy; Fig 5A) anchored to the poetry task each student had just completed. NASA-TLX was developed as a general workload inventory, and its subscales were therefore mapped provisionally onto Sweller’s intrinsic (ICL), extraneous (ECL), and germane (GCL) distinction under three assumptions. The Task Difficulty items (character density, syntactic complexity, semantic distance) serve as ICL indicators on the assumption that they reflect element interactivity rather than presentation. The reverse-scored Performance/Self-efficacy items serve as ECL indicators on the assumption that low perceived performance reflects load imposed by the presentation; this is the least direct link, because perceived performance also reflects self-efficacy and task success. The Mental Effort items (strategy use, self-explanation, schema building) serve as GCL indicators, consistent with Klepsch et al. [25], although effort also rises with intrinsic load and germane load is now regarded as the working-memory resources devoted to intrinsic load [1]. Because subjective ratings do not separate the load types unambiguously [15], the labels ICL, ECL, and GCL denote TLX-indexed indicators throughout this article, and findings that depend on their distinction are interpreted as preliminary (Section 4.6.4). The classical poetry comprehension test samples 12 works across three genres at four Bloom-taxonomy levels (literal 25%, inferential 30%, critical 25%, creative 20%; Cronbach’s α = 0.89, test–retest reliability 0.85), and the writing assessment is a composition of at least 500 characters within 60 minutes scored on six weighted dimensions (Fig 5C), with Cronbach’s α = 0.94, composite reliability ρ = 0.95, and 9-month test-retest reliability 0.88. The measurement model is depicted in Fig 5.

**Fig 5:**
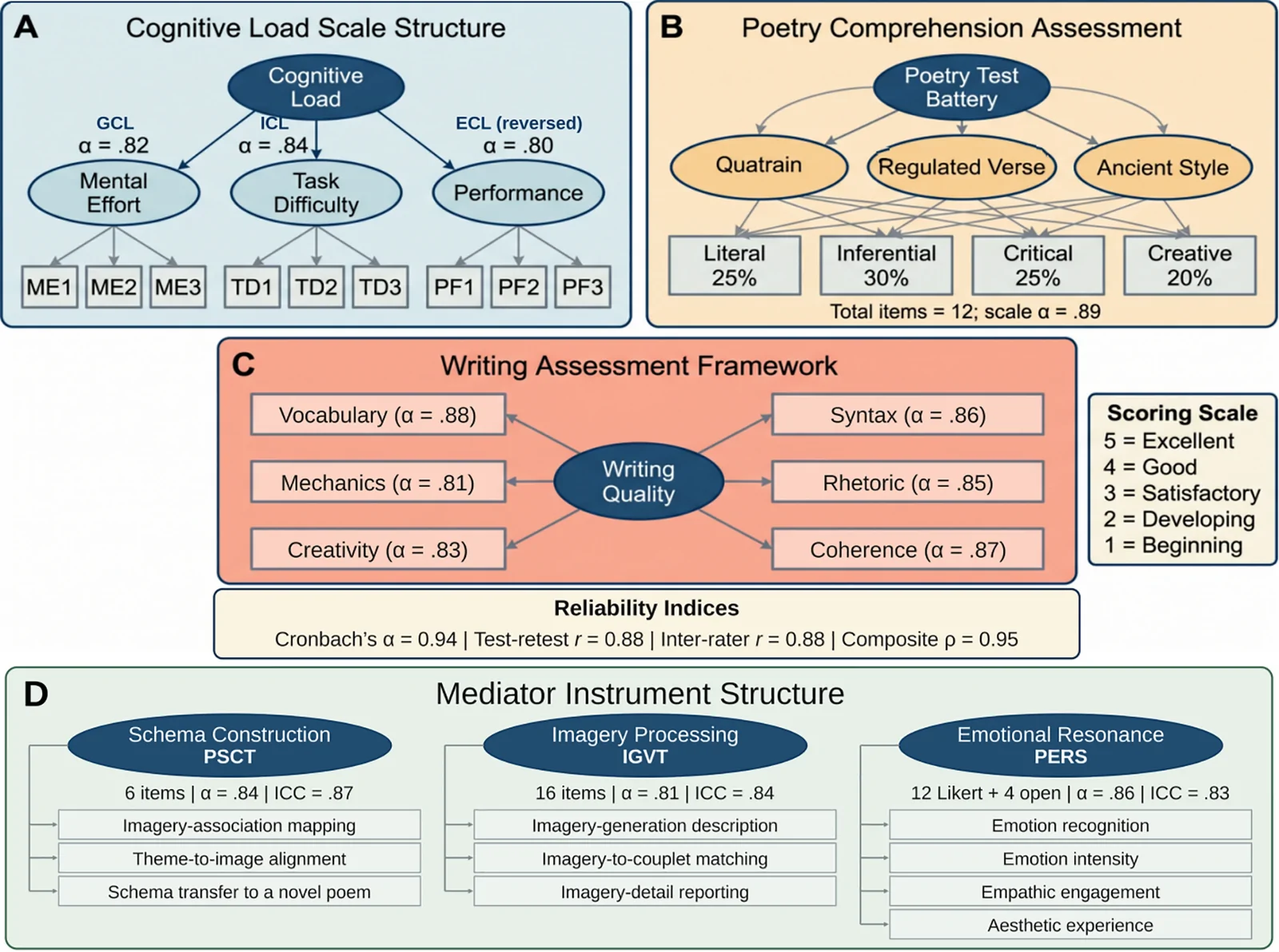
Measurement model components. (A) three-factor Cognitive Load Scale; (B) Poetry Comprehension Assessment across four cognitive levels; (C) six-dimension Writing Assessment framework; (D) three-mediator instrument structure (Schema Construction PSCT, Imagery Processing IGVT, Emotional Resonance PERS) with subdimensions and reliability indices.

Confirmatory factor analysis supports the three-factor structure of the Cognitive Load Scale (factor loadings 0.79 to 0.85, composite reliability 0.88), which supports the separability of the three TLX subscales but does not by itself establish that they correspond to the three load types distinguished by cognitive load theory.

Three mediators were measured at each wave after the poetry comprehension test and before the writing task: schema construction with the Poetry Schema Construction Task (PSCT; 6 open-response items), anchored in schema theory and meaningful learning theory [30,31] (Cronbach’s α = 0.84; two-rater ICC(2,1) = 0.87); imagery processing with the Imagery Generation and Verification Task (IGVT; 16 items), based on Kosslyn’s imagery paradigm and dual coding theory [32,33] (α = 0.81; ICC(2,1) = 0.84); and emotional resonance with the Poetry Emotional Resonance Scale (PERS; 12 Likert and 4 open-response items covering emotion recognition [34], emotion intensity, empathic engagement, and aesthetic experience; α = 0.86; ICC(2,1) = 0.83). Convergent and divergent validity were established against external measures [35]. Full item pools, scoring rubrics, and rater training materials are provided in Supplemental Appendices S2 in S1 File (PSCT), S3 (IGVT), and S4 (PERS), and wave-specific reliability and validity indices for the three instruments are summarized in Supplemental Table S5 in S1 File.

Each composition was scored independently by two of 12 trained secondary-school Chinese teachers, blinded to student, class, school, wave, and prior scores, with no rater scoring the same student in adjacent waves. Discrepancies of one scale point or less were averaged, and larger discrepancies were adjudicated by a senior third rater (4.7% of compositions). Inter-rater agreement was Pearson r = 0.88, ICC(2,k) = 0.91, and Cohen’s weighted κ = 0.79, and raters completed a six-hour calibration on 20 anchor essays before each wave.

Because the load-type mapping is provisional, its measurement properties were examined further in four analyses: one-factor, two-factor, and three-factor measurement models of the TLX subscales at each wave; configural, metric, and scalar invariance across Grades 3–6; a multitrait-multimethod analysis of the TLX and Klepsch instruments in the validation subsample; and a sensitivity model in which a single undifferentiated load score replaces the three components. These analyses are reported in Section 4.6.4 and Supplemental Table S6 in S1 File.

### 3.4. Data collection procedures

Each wave was completed within two weeks. Cognitive load was measured immediately after each poetry-learning task, the poetry comprehension test was paper-and-pen with a 40-minute limit, and the writing task was administered in a separate 60-minute session. Quality control comprised standardized administrator training, quiet-room administration, separate answer sheets, and three-tier monitoring (on-site supervision, random audio checks, and data verification).

### 3.5. Hierarchical linear model construction strategy

The hierarchical linear model was constructed from simple to complex, selecting the specification via AIC/BIC-driven stepwise addition of predictors and random effects. It is a four-level nested growth specification in which measurement occasions (Level 1, four waves per student) are nested within students (Level 2), students within classes (Level 3), and classes within schools (Level 4), and stable between-student baseline differences are absorbed into the Level-2 random intercept. The basic four-level model is:

Level 1 (measurement occasion, within a student):


$$\begin{array}{c} Y_{tijk}=\pi_{0ijk}+\pi_{1ijk}Time_{tijk}+\pi_{\text{2}}Time_{tijk}^{\text{2}}+e_{tijk} \end{array}$$ (1)

where $Y_{tijk}$ represents the writing ability score of student i in class j of school k at measurement occasion t, $Time_{tijk}$ is coded 0–3 for T1 to T4, $\pi_{0ijk}$ is the individual intercept (baseline level), $\pi_{1ijk}$ is the linear time slope, $\pi_{\text{2}}$ is the fixed quadratic time coefficient capturing deceleration, and $e_{tijk}$ is the within-student residual with variance $\sigma^{\text{2}}$. Time-varying predictors, including the group-mean-centred cognitive load components, enter Level 1 in the extended models of Equations (16) to (18).

Level 2 (Student, within a class):


$$\begin{array}{c} \pi_{0ijk}=\beta_{00jk}+\beta_{01jk}WM_{ijk}+\beta_{02jk}PK_{ijk}+r_{0ijk} \end{array}$$ (2)

$$\begin{array}{c} \pi_{1ijk}=\beta_{10jk}+\beta_{11jk}WM_{ijk}+\beta_{12jk}PK_{ijk}+r_{1ijk} \end{array}$$ (3)

$$\begin{array}{c} \left(r_{0ijk},r_{1ijk}{)}^{⊤}\;N(0,{𝐓}_{r}\right) \end{array}$$ (4)

where working-memory capacity ($WM_{ijk}$) and prior knowledge ($PK_{ijk}$) enter as grand-mean-centred student-level predictors of the intercept and linear slope, and the student-level random intercept and slope follow a bivariate normal distribution with covariance matrix ${𝐓}_{r}$, whose intercept variance is $\tau_{r_{\text{0}}}$.

Level 3 (Class, within a school), and Level 4 (School):


$$\begin{array}{c} \beta_{00jk}=\gamma_{000k}+\gamma_{001k}Teaching_{jk}+\gamma_{002k}ClassSize_{jk}+u_{00jk} \end{array}$$ (5)

$$\begin{array}{c} \beta_{10jk}=\gamma_{100k}+\gamma_{101k}Teaching_{jk}+u_{10jk} \end{array}$$ (6)

$$\begin{array}{c} \gamma_{000k}=\delta_{0000}+\delta_{0001}SchoolType_{k}+\delta_{0002}Location_{k}+v_{000k},\gamma_{100k}=\delta_{1000}+v_{100k} \end{array}$$ (7)

where $u_{00jk}$ and $u_{10jk}$ are class-level random effects with intercept variance $\tau_{u_{00}}$, $v_{000k}$ and $v_{100k}$ are school-level random effects with intercept variance $\tau_{v_{000}}$, $Teaching_{jk}$ and $ClassSize_{jk}$ are class-level predictors, and $SchoolType_{k}$ and $Location_{k}$ are school-level predictors.

The growth-curve model captures the nonlinear development of writing ability with a quadratic term:


$$\begin{array}{c} f\left(Time_{tijk}\right)=\pi_{0ijk}+\pi_{1ijk}Time_{tijk}+\pi_{\text{2}}Time_{tijk}^{\text{2}} \end{array}$$ (8)

Individual parameters decompose into fixed and random components, with ${𝐰}_{ijk}$ collecting the centred student-, class-, and school-level predictors of Equations (2), (3), and (5) to (7):


$$\begin{array}{c} \pi_{0ijk}=\delta_{0000}+{𝐰}_{ijk}^{⊤}\gamma_{\text{0}}+v_{000k}+u_{00jk}+r_{0ijk} \end{array}$$ (9)

$$\begin{array}{c} \pi_{1ijk}=\delta_{1000}+{𝐰}_{ijk}^{⊤}\gamma_{\text{1}}+v_{100k}+u_{10jk}+r_{1ijk} \end{array}$$ (10)

Let $\sigma^{\text{2}}$ denote the within-student residual variance (Level 1), $\tau_{r_{\text{0}}}$ the between-student variance in the intercept (Level 2), $\tau_{u_{00}}$ the between-class variance (Level 3), and $\tau_{v_{000}}$ the between-school variance (Level 4), so that the total outcome variance is $V_{total}=\sigma^{\text{2}}+\tau_{r_{\text{0}}}+\tau_{u_{00}}+\tau_{v_{000}}$. The class-level and school-level intraclass correlation coefficients are:


$$\begin{array}{c} ICC_{class}=\frac{\tau_{u_{00}}}{V_{total}} \end{array}$$ (11)

$$\begin{array}{c} ICC_{school}=\frac{\tau_{v_{000}}}{V_{total}} \end{array}$$ (12)

The cumulative higher-level dependency is $(\tau_{u_{00}}+\tau_{v_{000}})/V_{total}$. Because $\tau_{v_{000}}$ is estimated from 12 schools, $ICC_{school}$ is reported with profile-likelihood and parametric bootstrap intervals rather than with a Wald standard error.

Model fit is evaluated with deviance information criterion (DIC), Akaike information criterion (AIC), and Bayesian information criterion (BIC):


$$\begin{array}{c} DIC=-2logL+2p_{DIC} \end{array}$$ (13)

$$\begin{array}{c} AIC=-2logL+2k \end{array}$$ (14)

$$\begin{array}{c} BIC=-2logL+k\cdot ln\left(n\right) \end{array}$$ (15)

Where logL is the log-likelihood value, $p_{DIC}$ is the effective number of parameters, k is the number of model parameters, and n is the sample size.

The mediation effect is tested with a multilevel model (equations shown for schema construction; the imagery and emotion pathways are specified in the same way):


$$\begin{array}{c} Schema_{ijk}=a_{\text{0}}+a_{\text{1}}CogLoad_{ijk}+\nu_{ijk} \end{array}$$ (16)

$$\begin{array}{c} Writing_{ijk}={c^{\prime }}_{\text{0}}+{c^{\prime }}_{\text{1}}CogLoad_{ijk}+b_{\text{1}}Schema_{ijk}+\varepsilon_{ijk} \end{array}$$ (17)

Indirect effects are calculated as $a_{\text{1}}\cdot b_{\text{1}}$, with confidence intervals constructed using Monte Carlo methods. Because cognitive load, the mediators, and writing ability are observed within the same wave, these indirect effects are statistical indirect associations under the hypothesized ordering; their temporal ordering and their sensitivity to unmeasured mediator-outcome confounding are examined in the sensitivity analyses described below.

Cross-level moderation of the class-level context on the individual-level relationship is tested by:


$$\begin{array}{c} \begin{array}{c} Y_{ijk}=\gamma_{000}+\gamma_{100}CogLoad_{ijk}+\gamma_{010}Teaching_{jk}+\\ \gamma_{110}CogLoad_{ijk}\times Teaching_{jk}+u_{0jk}+r_{ijk} \end{array} \end{array}$$ (18)

Group-mean centering (CWC) is applied to Level-1 predictors and grand-mean centering (CGM) to Level-2 through Level-4 predictors. Restricted maximum likelihood (REML) is used for the main four-level model, and variance components are reported with profile-likelihood and parametric bootstrap confidence intervals because Wald standard errors are unreliable for variance parameters estimated from few clusters and close to the boundary of the parameter space [36]; each variance component is tested against a 50:50 mixture of $\chi^{\text{2}}$ distributions, and school-level and cross-level fixed effects are tested with Kenward-Roger degrees of freedom [37]. Complex nested blocks (moderated mediation with random slopes) are estimated with Bayesian methods under the priors specified in Algorithm 1 [38] and re-estimated under three alternative priors (Section 4.6.5), with convergence confirmed by a rank-normalized $\hat{R}$ <1.01 and bulk and tail effective sample sizes of at least 400. Models were fitted in R 4.3.2 using lme4, lmerTest, brms, and lavaan [36–39], with mice used for fold-wise imputation. Section 4.5 reports continuous-outcome regression with predictions ${\hat{Y}}_{tijk}$ formed from the fixed effects and the best linear unbiased predictions (BLUPs) estimated in the training folds (Section 3.6), and a three-band classification (Low, Medium, High) derived by discretising $\hat{Y}$ at grade-normed 30th and 70th percentile cutoffs computed within each training fold. Feature importance is the leave-one-covariate-out $\Delta R^{\text{2}}$ with each reduced model refitted in the training folds, reported as the percentage increase in out-of-fold MSE.

Because the highest level of the model contains 12 schools, the stability of the principal Level-4 and cross-level results (the school-level intercept variance and $ICC_{school}$, the effects of school type and geographic location, the interactions of cognitive load with teaching method, grade, and class environment, and the index of moderated mediation) is examined with five analyses: 12 leave-one-school-out refits; a school-level wild cluster bootstrap using Webb weights (B =9,999), replaced by a school-level case bootstrap for the variance component and $ICC_{school}$; Bayesian re-estimation with half-Cauchy(0, 5), half-Student-t(3, 0, 10), and uniform(0, 30) priors on the random-effect standard deviations in addition to the reference inverse-gamma prior; a model with school fixed effects for coefficients that vary within schools; and a Monte Carlo simulation of 1,000 datasets generated from the fitted model with the observed design. A coefficient is classified as stable when its sign is unchanged in all 12 refits, its Kenward-Roger and wild-bootstrap 95% intervals exclude zero, its 95% credible interval excludes zero under all four priors, and the simulated coverage of its Kenward-Roger interval is at least 0.925; coefficients that fail any criterion are reported as exploratory, and a variance component whose intervals reach the boundary of the parameter space is reported as imprecise (Section 4.6.5).

The random-intercept cross-lagged panel model (RI-CLPM) reported in Section 4.3.5 separates stable between-student differences from within-student fluctuations, and its cross-lagged paths are interpreted as within-person lagged associations rather than causal effects. The assumed ordering is further examined by re-estimating the RI-CLPM with grade, gender, school location, and teaching method as time-invariant covariates and poetry comprehension as a time-varying covariate, by a temporally ordered mediation model from cognitive load at one wave through each mediator at the next to writing ability at the wave after (T1 to T3 and T2 to T4), and by the residual correlation between the mediator and outcome equations that would reduce each indirect effect to zero (Section 4.6.6).

Algorithm 1 Four-Level Predictive Model Construction and Validation (core procedure)

**Input**: Y ∈ R^(N × T) writing-ability score (N = 1920, T = 4); $X_{L1}$ ∈ R^(N × T × p_1_) Level-1 features {ICL, ECL, GCL, poetry_comp, WM, PK}; $X_{L2}$ ∈ R^(K × p_2_) class features; $X_{L3}$ ∈ R^(J × p_3_) school features; M ∈ R^(N × T × 3) mediator matrix {schema, imagery, emotion}.

**Output:** trained parameter set Θ̂; out-of-fold predictions ${\hat{Y}}^{A}$, ${\hat{Y}}^{B}$, ${\hat{Y}}^{C}$ under validation designs A, B, and C; indirect-effect vector ab̂; cross-level interaction γ̂; feature-importance vector φ; stability classification of Level-4 and cross-level estimates.

1. Preprocessing for the full-sample inferential models: CWC for X_L1, CGM for X_L2 and X_L3; multiple imputation (m = 20 chains, predictive mean matching).

2. Null-model variance decomposition by REML; ICC_class and ICC_school with 95% profile-likelihood and parametric bootstrap CIs; boundary-corrected likelihood-ratio tests of each variance component.

3. Growth-function order selection over {linear, quadratic, cubic}; retain a higher-order term only if ΔAIC ≤ −10.

4. Multilevel mediation: for each mediator Mᵢ, a-path Mᵢ = a₀ + a_1_· CogLoad + ε_a and b-path Y  = c₀ + c′· CogLoad + b_1_· Mᵢ + ε_b; ab̂ᵢ = a_1_·b_1_ with 5000-replicate Monte-Carlo CIs.

5. Cross-level moderation: interaction terms (CogLoad × Teaching) and (CogLoad × Grade); school-level and cross-level fixed effects tested with Kenward-Roger degrees of freedom.

6. Bayesian refinement for complex nested blocks: γ ~ N(0, 10²); σ² ~ Inv-Gamma(0.001, 0.001); sensitivity priors half-Cauchy(0, 5), half-Student-t(3, 0, 10), Uniform(0, 30) on random-effect SDs; 4 HMC chains × 10 000 iterations (5 000 burn-in); rank-normalized $\hat{R}$ < 1.01 and bulk and tail ESS ≥ 400.

7. Fold-wise validation for design A (student-grouped 10-fold, grade-balanced), design B (leave-one-class-out, 48 folds), and design C (leave-one-school-out, 12 folds). For each fold f:

7.1 Estimate PMM imputation models (m = 20) on the training folds; exclude validation outcomes from imputation.

7.2 Center Level-1 predictors on student means and Level-2 to Level-4 predictors on training-fold grand means.

7.3 Reselect growth order and random-effects structure by AIC/BIC; refit the four-level model by REML.

7.4 Predict validation observations from training-fold fixed effects and BLUPs following Eqs. (19) to (21).

7.5 Derive band cutoffs and band probabilities from training-fold scores and residuals.

7.6 Compute leave-one-covariate-out importance $\varphi_{p}$ from models refitted on the training folds.

7.7 Average predictions over the m imputed datasets.

8. Report $R^{\text{2}}$, RMSE, MAE, calibration, accuracy, and ROC-AUC for designs A, B, and C; robustness checks (complete-case analysis, Huber M-estimation, bootstrap B = 1000); Holm and Benjamini-Hochberg control; small-cluster, measurement, and confounding sensitivity analyses.

End.

### 3.6. Validation design and leakage control

The predictive performance of a multilevel model depends on which units are unseen at the time of prediction, and the level of out-of-sample generalization that student-level splitting supports is limited to new students drawn from higher-level contexts already represented in the training folds. Three prediction targets are therefore distinguished. The primary target, new students within the sampled classes and schools, is the criterion for the O6 acceptance thresholds and is evaluated with student-grouped 10-fold cross-validation (design A), in which all four waves of a student are assigned to the same fold and each fold contains 48 students from each grade (192 students and 768 observations). Generalization to entirely unseen classes within the sampled schools is evaluated with leave-one-class-out cross-validation over the 48 classes (design B), and generalization to entirely unseen schools with leave-one-school-out cross-validation over the 12 schools (design C), in which the four classes and 160 students of a school are held out together. Designs B and C are the cluster-aware validation analyses that bound the transfer of the model beyond the sampled higher-level units, and they are reported without acceptance thresholds because O6 does not preset criteria for those two targets. Fig 6A summarizes the three designs and the random effects that enter each prediction.

**Fig 6:**
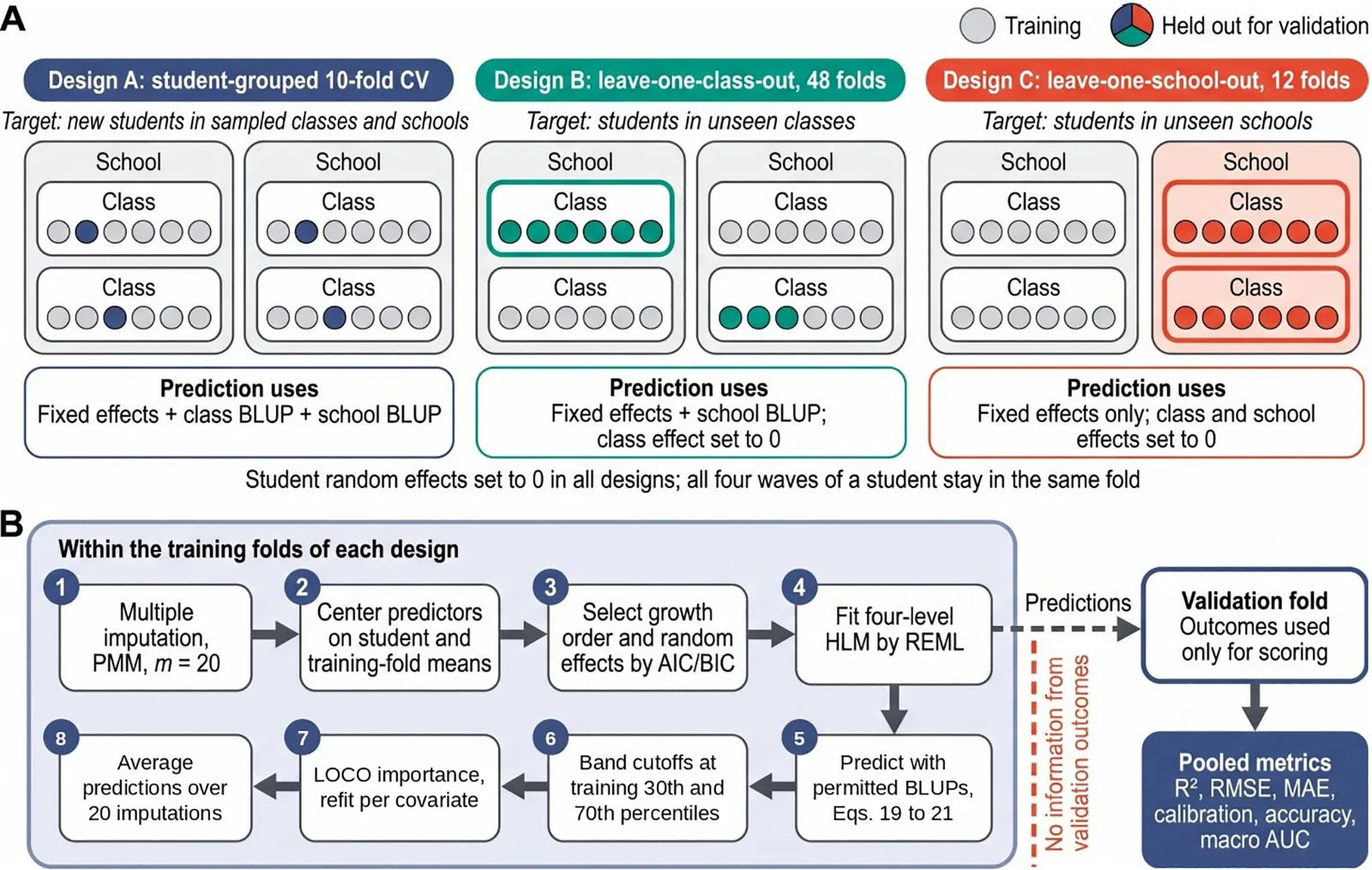
Validation designs and fold-wise pipeline. (A) student-grouped 10-fold cross-validation (Design A), leave-one-class-out cross-validation (Design B), and leave-one-school-out cross-validation (Design C), with the random effects that enter each prediction; (B) sequence of data-dependent steps carried out within the training folds of each design.

All data-dependent steps are carried out within folds, and their sequence is shown in Fig 6B and documented in Supplemental Table S7 in S1 File. Multiple imputation with predictive mean matching (m =20) is estimated on the training folds only, validation outcomes are neither used in the imputation models nor imputed, and validation observations without an observed outcome are excluded from the performance metrics. Level-1 predictors are centered on each student’s own mean, and the grand means for Level-2 to Level-4 predictors are computed in the training folds. The growth order and random-effects structure are reselected in each training fold by AIC/BIC and the four-level model is re-estimated by REML, with selection frequencies reported. The grade-normed 30th and 70th percentile band cutoffs are computed from observed training-fold scores, the band probabilities for ROC-AUC from the training-fold residual distribution, each leave-one-covariate-out model is refitted in the training folds, and predictions are averaged over the 20 imputed datasets.

The random effects that enter a prediction depend on which units are represented in the training folds. Let ${\hat{\gamma }}^{\left(-f\right)}$ denote the fixed effects estimated without fold f, and let ${\hat{𝐮}}_{jk}^{\left(-f\right)}=({\hat{u}}_{00jk},{\hat{u}}_{10jk}{)}^{⊤}$ and ${\hat{𝐯}}_{k}^{\left(-f\right)}=({\hat{v}}_{000k},{\hat{v}}_{100k}{)}^{⊤}$ denote the BLUPs of the class-level and school-level random intercepts and time slopes from the same fit. With ${𝐱}_{tijk}$ denoting the fixed-effect design vector and ${𝐳}_{tijk}=(1,Time_{tijk}{)}^{⊤}$ the random-effect design vector, the predictions under the three designs are:


$$\begin{array}{c} {\hat{Y}}_{tijk}^{\text{A}}={𝐱}_{tijk}^{⊤}{\hat{\gamma }}^{\left(-f\right)}+{𝐳}_{tijk}^{⊤}{\hat{𝐮}}_{jk}^{\left(-f\right)}+{𝐳}_{tijk}^{⊤}{\hat{𝐯}}_{k}^{\left(-f\right)} \end{array}$$ (19)

$$\begin{array}{c} {\hat{Y}}_{tijk}^{\text{B}}={𝐱}_{tijk}^{⊤}{\hat{\gamma }}^{\left(-f\right)}+{𝐳}_{tijk}^{⊤}{\hat{𝐯}}_{k}^{\left(-f\right)} \end{array}$$ (20)

$$\begin{array}{c} {\hat{Y}}_{tijk}^{\text{C}}={𝐱}_{tijk}^{⊤}{\hat{\gamma }}^{\left(-f\right)} \end{array}$$ (21)

Student-level random effects are set to zero in all three designs because every observation of a validation student is held out. Design A uses the BLUPs of the class and school of each validation student, design B sets the effects of the held-out class to zero while retaining the school-level BLUPs estimated from the other three classes, and design C sets all random effects of the held-out school to zero. The BLUPs ${\hat{𝐛}}^{\left(-f\right)}=\hat{𝐆}{𝐙}_{-f}^{⊤}{\hat{𝐕}}_{-f}^{-1}({𝐘}_{-f}-{𝐗}_{-f}{\hat{\gamma }}^{\left(-f\right)})$ are computed within each training fold from the training-fold outcomes, design matrices, and variance components, so outcome information from the validation fold enters neither the fixed effects, the variance components, nor any random-effect prediction. In design A, the class and school BLUPs of a validation student are estimated jointly from the training-fold observations of that student’s classmates and of the other three classes of that school; the outcomes of the validation student at all four measurement waves are excluded from both. In design B, the class BLUP is unavailable because every observation in the held-out class is held out, and the school BLUP is estimated from the training-fold observations of the other three classes of the held-out class’s school. In design C, every class and school random effect of the held-out school is set to zero because no training-fold observation from that school is available for either BLUP.

Predictive performance is summarized by $R^{\text{2}}$, RMSE, MAE, the calibration slope and intercept, three-band classification accuracy, and band-specific and macro-averaged ROC-AUC. Fold-level metrics of design A are summarized by their mean and standard deviation, with the 95% confidence interval of $R^{\text{2}}$ from 1,000 bootstrap resamples of students; designs B and C are computed from the pooled out-of-fold predictions. Fold-level and school-level results are given in Supplemental Table S8 in S1 File, and the three designs are compared in Section 4.5.1.

## 4. Research results

### 4.1. Descriptive statistics and preliminary analysis

Writing-ability scores rose steadily from T1 (M = 65.43) to T4 (M = 78.92) with declining standard deviations (Table 2), indicating a gradual convergence of scores across students.

**Table 2:** Descriptive statistics of main variables (N = 1920).

Variable	T1 Mean(SD)	T2 Mean(SD)	T3 Mean(SD)	T4 Mean(SD)
Writing Total Score	65.43(11.02)	70.28(10.21)	74.65(9.33)	78.92(8.37)
Vocabulary Richness	13.24(2.75)	14.36(2.61)	15.41(2.41)	16.33(2.24)
Syntactic Complexity	12.87(2.96)	13.92(2.74)	14.98(2.49)	15.93(2.28)
Rhetorical Devices	8.94(2.36)	9.82(2.17)	10.72(1.97)	11.48(1.78)
Thematic Coherence	14.35(2.56)	15.25(2.33)	16.15(2.12)	16.97(1.91)
Creative Expression	9.79(2.50)	10.25(2.32)	10.37(2.12)	10.79(1.94)
Language Mechanics	6.24(1.62)	6.68(1.51)	7.02(1.39)	7.42(1.28)
Intrinsic Cog Load	3.82(0.78)	3.65(0.72)	3.48(0.69)	3.31(0.65)
Extraneous Cog Load	3.95(0.82)	3.58(0.76)	3.22(0.71)	2.89(0.67)
Germane Cog Load	2.76(0.69)	3.12(0.65)	3.45(0.61)	3.78(0.58)
Poetry Comprehension	68.45(14.32)	73.28(12.86)	77.65(11.43)	81.92(10.15)

The six writing dimensions are scored in weighted points (vocabulary, syntax, and coherence 0–20; rhetoric and creativity 0–15; mechanics 0–10) and sum to the writing total score (0–100).

*Intrinsic and extraneous load decreased across waves (from 3.82 to 3.31 and from 3.95 to 2.89), while germane load increased (from 2.76 to 3.78), consistent with a redistribution of cognitive resources toward schema building as tasks become familiar.*

Grade 3 shows the steepest growth (period-to-period increments of 12.8%, 9.3%, and 7.8%), whereas Grade 6 growth is flatter at 3–4% (Fig 7), consistent with a ceiling effect on writing-ability development.

**Fig 7:**
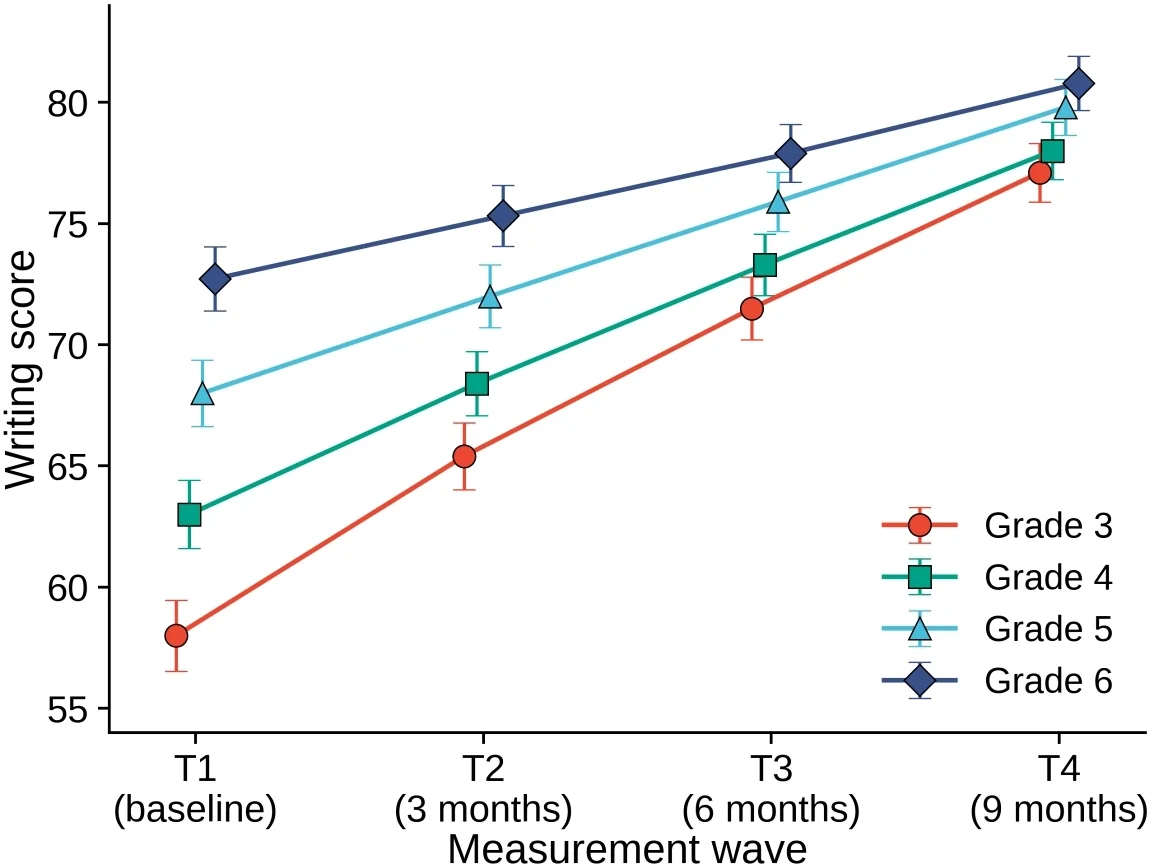
Writing development trajectories across grades (error bars, ± 1, standard error adjusted for class and school clustering).

Bivariate correlations (Table 3) show that writing ability is strongly positively correlated with poetry comprehension (r = 0.68, p < .001), moderately with germane cognitive load (r = 0.45, p < .001), and negatively with extraneous cognitive load (r = −0.41, p < .001). These are between-student correlations at T4, whereas the O1 coefficients in Table 9 are within-student associations of the group-mean-centred load components pooled across the four waves, so the two sets of values are not directly comparable.

**Table 3:** Correlation matrix of main variables (T4 Data).

Variables	1	2	3	4	5	6	7	8	9
1. Writing Total	–								
2. Poetry Comp	.68***	–							
3. ICL	−.14**	−.15**	–						
4. ECL	−.41***	−.42***	.45***	–					
5. GCL	.45***	.48***	−.08*	−.52***	–				
6. Schema	.58***	.62***	−.10*	−.35***	.56***	–			
7. Imagery	.52***	.55***	−.08	−.30***	.48***	.65***	–		
8. Emotion	.46***	.50***	−.06	−.28***	.45***	.58***	.62***	–	
9. Grade	.35***	.32***	−.18***	−.25***	.22***	.28***	.25***	.20***	–

Note: ICL = intrinsic cognitive load, ECL = extraneous cognitive load, GCL = germane cognitive load *p < .05, **p < .01, ***p < .001

### 4.2. Measurement model validation

#### 4.2.1. Reliability and validity testing.

As shown in Table 4, Cronbach’s α was at least 0.80 for every scale and reached 0.94 for the writing assessment, and composite reliability (CR) and average variance extracted (AVE) supported convergent and discriminant validity.

**Table 4:** Reliability and validity indices of measurement tools.

Scale/Dimension	Items	α	CR	AVE	Test-retest r
Cognitive Load Scale	9	0.86	0.88	0.52	0.82
- Mental Effort	3	0.82	0.83	0.62	0.78
- Task Difficulty	3	0.84	0.85	0.65	0.80
- Performance	3	0.80	0.81	0.58	0.76
Poetry Comprehension	12	0.89	0.90	0.48	0.85
Writing Assessment	30	0.94	0.95	0.58	0.88
- Vocabulary	5	0.88	0.89	0.62	0.84
- Syntax	5	0.86	0.87	0.60	0.82
- Rhetoric	5	0.85	0.86	0.58	0.80
- Coherence	5	0.87	0.88	0.61	0.83
- Creativity	5	0.83	0.84	0.56	0.78
- Mechanics	5	0.81	0.82	0.54	0.76
Schema Construction Task (PSCT)	6	0.84	0.87	0.62	0.81
Imagery Generation and Verification Task (IGVT)	16	0.81	0.84	0.58	0.79
Poetry Emotional Resonance Scale (PERS)	16	0.86	0.88	0.55	0.83

Confirmatory factor analysis (Fig 8) showed good fit for both the three-factor cognitive load model (χ²/df = 2.35, CFI = 0.96, TLI = 0.95, RMSEA = 0.048, SRMR = 0.042) and the six-factor writing model (χ²/df = 2.68, CFI = 0.94, TLI = 0.93, RMSEA = 0.052, SRMR = 0.045). All factor loadings exceeded 0.60.

#### 4.2.2. Measurement invariance testing.

Cross-time measurement invariance was tested from configural to strict invariance (Fig 8C); all ΔCFI values fell below the 0.01 criterion, supporting the growth-model analyses below.

**Fig 8:**
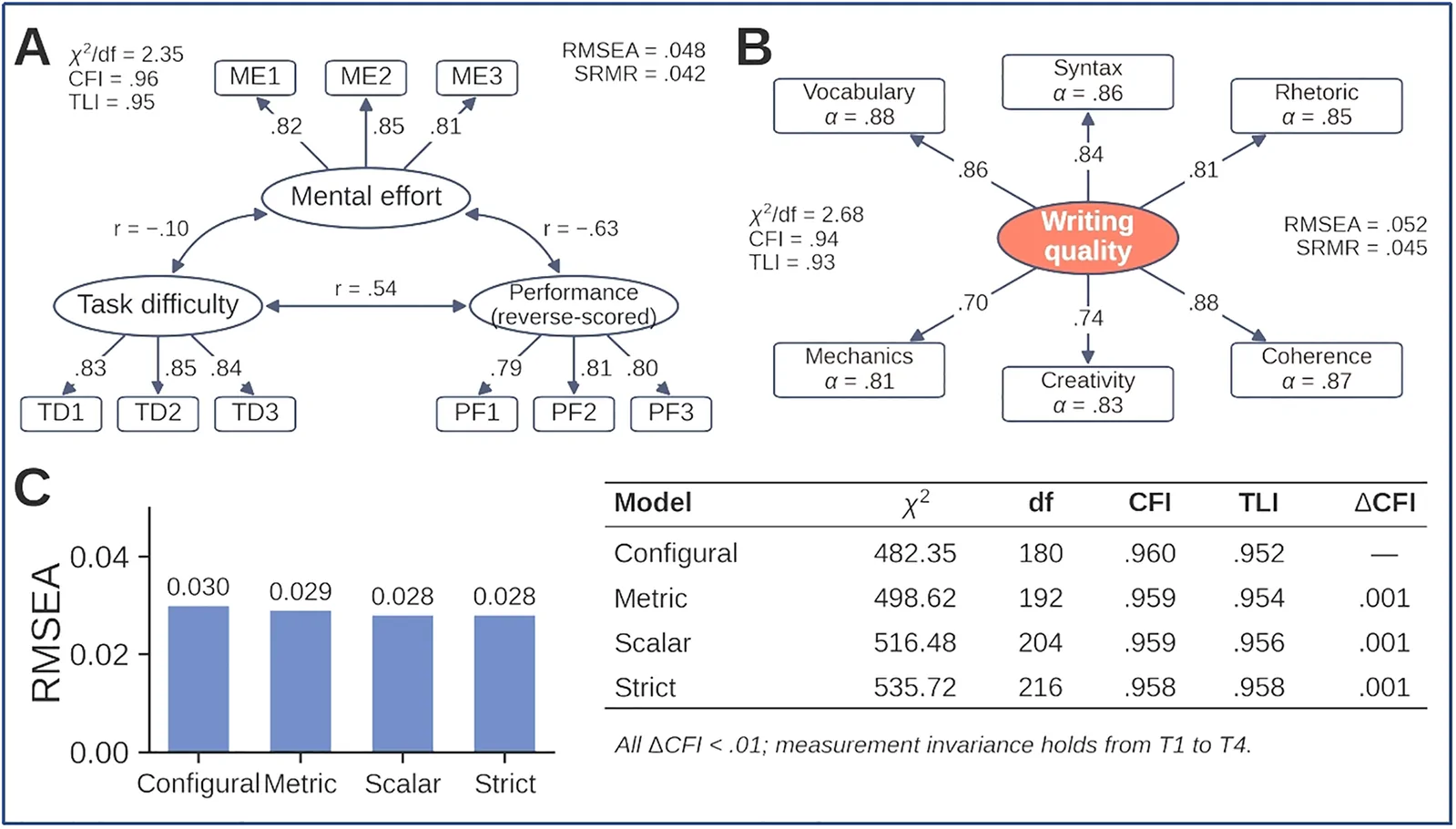
Confirmatory factor analysis results.

#### 4.2.3. Discriminant validity among core constructs.

Discriminant validity between the mediators and the writing outcome was supported by three analyses. Following Henseler et al. [40], all heterotrait-monotrait ratios among the seven core constructs fell below 0.85 (Table 5), with values between the mediators and the writing outcome from 0.61 to 0.70. The square root of average variance extracted ($\sqrt{AVE}$) of each construct exceeded its correlations with other constructs (Schema $\sqrt{AVE}$ = 0.79 > 0.65; Imagery $\sqrt{AVE}$ = 0.76 > 0.65; Emotion $\sqrt{AVE}$ = 0.74 > 0.62; Writing $\sqrt{AVE}$ = 0.76 > 0.68), and in an exploratory factor analysis no mediator item loaded ≥ 0.40 on a writing or comprehension factor and no writing item loaded 0.40 on a mediator factor. The indirect effects in Section 4.4 therefore reflect shared variance between distinct constructs rather than measurement overlap.

**Table 5:** Heterotrait-monotrait ratio of correlations (HTMT) among core constructs (T4 data).

Construct	1	2	3	4	5	6	7
1. ICL	–						
2. ECL	0.51	–					
3. GCL	0.14	0.58	–				
4. Poetry Comprehension	0.18	0.47	0.54	–			
5. Schema Construction	0.13	0.38	0.63	0.72	–		
6. Imagery Processing	0.11	0.35	0.57	0.63	0.71	–	
7. Emotional Resonance	0.09	0.28	0.49	0.57	0.65	0.68	–
8. Writing Ability	0.14	0.43	0.48	0.75	0.70	0.65	0.61

All HTMT values are below the 0.85 conservative threshold, indicating adequate discriminant validity.

### 4.3. Hierarchical linear model analysis results

#### 4.3.1. Null model and variance decomposition.

The null model decomposes writing-ability variance into within-student variance across measurement occasions (40.3%), between-student differences (27.9%), class level (23.5%), and school level (8.3%) (Table 6). Under the specifications given in Equations (11) and (12), the class-level ICC is 0.235 and the school-level ICC is 0.083, with a cumulative higher-level dependency of 0.318, supporting the four-level specification. Because the school-level variance is estimated from 12 clusters, all variance components are reported with 95% profile-likelihood and parametric bootstrap intervals and boundary-corrected likelihood-ratio tests. The class-level variance is estimated with adequate precision (95% profile-likelihood interval [18.11, 47.62]), whereas the school-level variance is supported by the boundary-corrected likelihood-ratio test (${\overset{―}{\chi }}^{\text{2}}$ = 3.53, p = .030) but its 95% profile-likelihood interval extends from the boundary of the parameter space to 38.69, so the magnitude of $ICC_{school}$ (0.083; 95% interval [0.000, 0.260]) is estimated imprecisely and is not interpreted substantively (Section 4.6.5)

**Table 6:** Variance decomposition results of null model.

Level	Variance Component	Proportion	SE	LRT (${\overset{―}{\chi }}^{2}$)	ICC	95% CI (profile / bootstrap)
Level 1: Occasion (σ²)	48.72	40.30%	0.91	–	–	[46.98, 50.54] / [47.00, 50.47]
Level 2: Student (τ_r₀)	33.73	27.90%	1.52	1472.14***	–	[30.87, 36.83] / [30.87, 36.75]
Level 3: Class (τ_u₀₀)	28.42	23.50%	6.97	614.48***	0.235	[18.11, 47.62] / [16.30, 43.86]
Level 4: School (τ_v₀₀₀)	10.03	8.30%	7.63	3.53*	0.083	[0.00, 38.69] / [0.00, 28.18]
Total	120.9	100%	–	–	–	–
Model Fit						
−2LL	54341.4					
AIC	54351.4					
BIC	54386.1					

$\sigma^{\text{2}}$ = within-student residual variance (Level 1); $\tau_{r_{\text{0}}}$ = between-student intercept variance (Level 2); $\tau_{u_{00}}$ = between-class intercept variance (Level 3); $\tau_{v_{000}}$ = between-school intercept variance (Level 4). Under Equations (11) and (12), $ICC_{class}$ = $\tau_{u_{00}}$ / $V_{total}$ = 28.42 / 120.90 = 0.235 and $ICC_{school}$ = $\tau_{v_{000}}$ / $V_{total}$ = 10.03 / 120.90 = 0.083; the cumulative higher-level dependency ($\tau_{u_{00}}$ + $\tau_{v_{000}}$) / $V_{total}$ = 0.318 is a design-effect indicator rather than an ICC. Profile-likelihood intervals were obtained by inversion of the REML profile [36] and bootstrap intervals from 1,000 simulated datasets. LRT denotes the boundary-corrected likelihood-ratio statistic of each variance component, referred to a 50:50 mixture of $\chi^{\text{2}}$ distributions with 0 and 1 degree of freedom; the asymptotic SEs are listed for reference only. * p < .05, *** p < .001

#### 4.3.2. Multicollinearity diagnostics among the three cognitive load predictors.

Variance inflation factors of the group-mean-centred load predictors (ICL VIF = 1.63, ECL VIF = 2.14, GCL VIF = 1.72), tolerance values of 0.47 to 0.61, and a maximum condition index of 12.4 indicate that, despite their moderate correlations at T4 (Table 3), the three predictors retain sufficient statistical independence for the model to identify their unique associations with writing outcomes.

#### 4.3.3. Growth model comparison.

The quadratic growth model provided the best fit among candidate specifications (Fig 9). Adding a quadratic term significantly improved the fit over the linear specification (Δχ²(1) = 15.12, p < .001; ΔAIC = −13.12), whereas a cubic term produced no further gain (ΔAIC = +1.42). A segmented-growth alternative also fit worse than the quadratic model.

**Fig 9:**
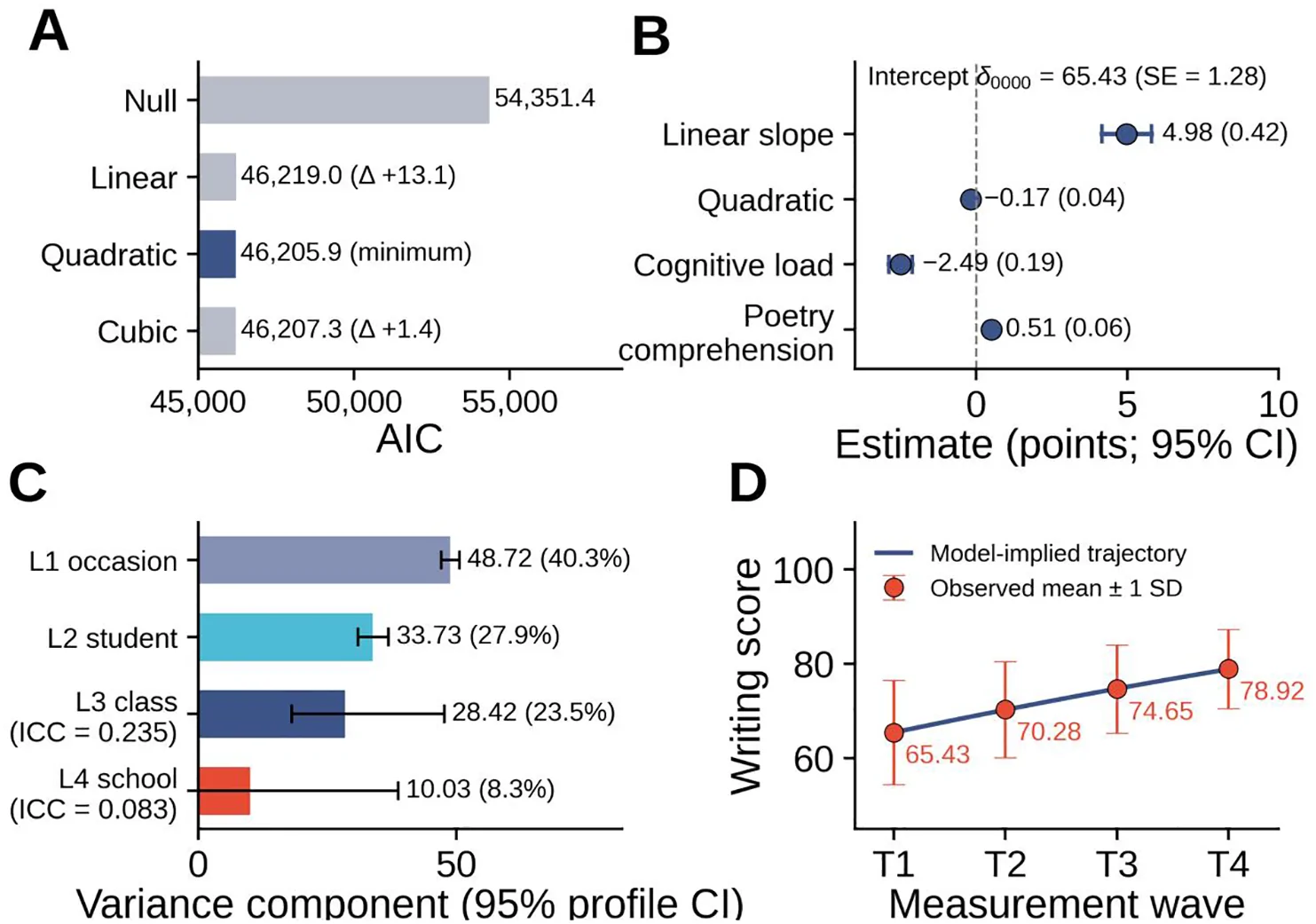
Growth model comparison and parameter estimates. (A) Akaike information criterion of the null, linear, quadratic, and cubic growth specifications; (B) fixed-effect estimates of the quadratic growth model with 95% confidence intervals; (C) four-level variance decomposition of the null model with 95% profile-likelihood intervals (Table 6); (D) observed wave means (± 1 SD, Table 2) and the model-implied quadratic trajectory.

The quadratic growth-model parameters were: mean initial level 65.43 (SE = 1.28, p < .001), linear growth rate 4.98 points per wave (SE = 0.42, p < .001), and quadratic coefficient −0.17 (SE = 0.04, p < .001), yielding the mild deceleration pattern typical of a learning curve (implied wave means 65.43, 70.24, 74.71, and 78.84, in line with Table 2). Initial level correlated negatively with growth rate (r = −0.32, p < .01), consistent with a compensatory-development pattern in which lower-starting students grow more rapidly.

#### 4.3.4. Cross-level interaction effects.

Cross-level interaction analysis examined three interactions between class-level and student-level predictors (Fig 10). The interaction between teaching method and cognitive load, the confirmatory O5 test (Section 4.5.2), was significant (γ = 0.47, SE = 0.17, Kenward-Roger p = .011, Holm-adjusted p = .011) and remained significant under all five small-cluster sensitivity analyses of Section 4.6.5 (LOSO range [0.39, 0.56]; wild-bootstrap 95% CI [0.11, 0.83]; KR coverage 0.945): in interactive-teaching classes the negative association of cognitive load with writing ability was weaker than under traditional instruction. The exploratory interactions with grade (γ = 0.39, Benjamini-Hochberg-adjusted p = .023) and class environment (γ = 0.31, adjusted p = .038) failed the wild-bootstrap and coverage criteria and were prior-sensitive, so they are reported as hypothesis-generating.

**Fig 10:**
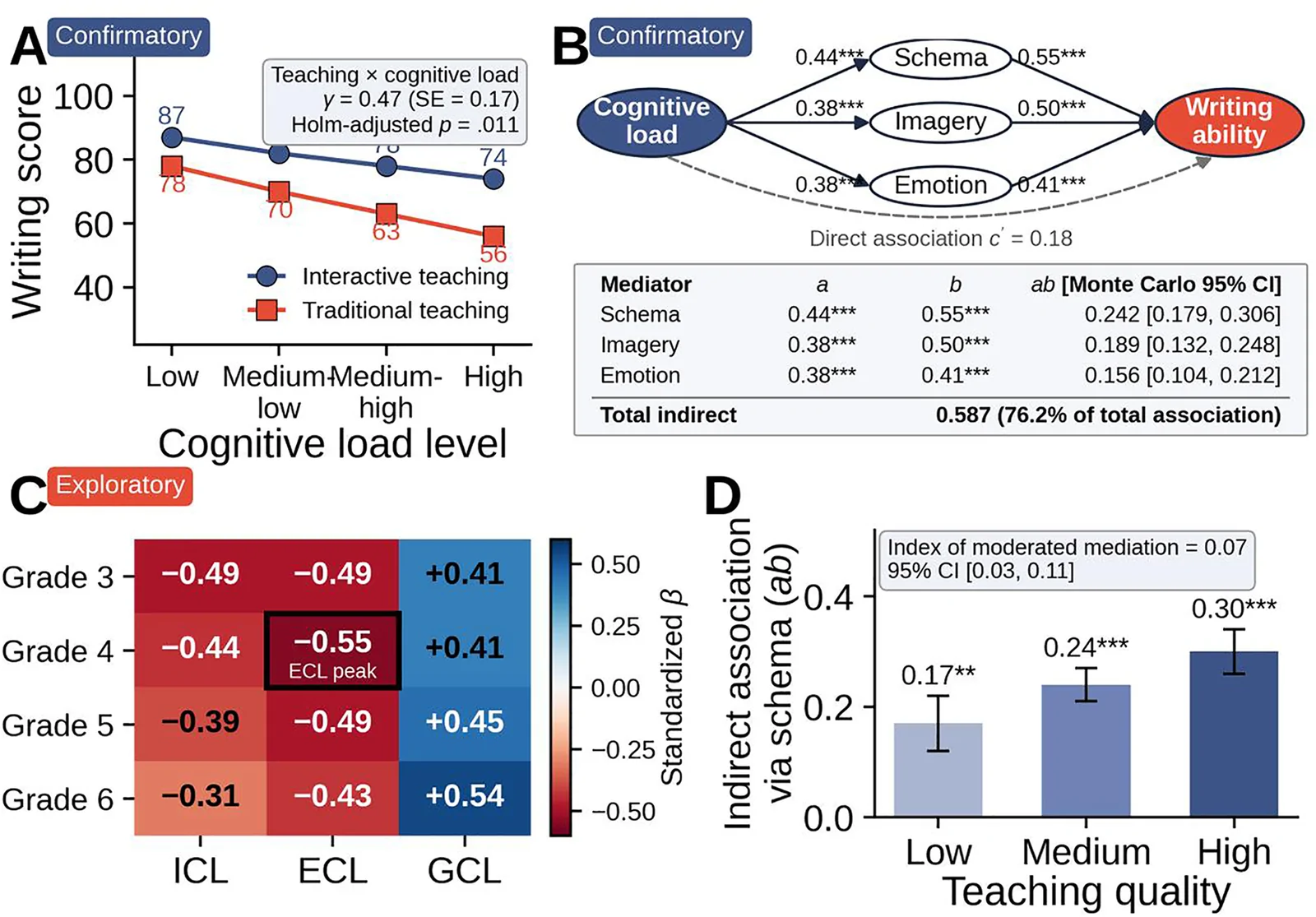
Cross-level interaction and indirect associations. (A) teaching method × cognitive load interaction (confirmatory); (B) indirect associations of cognitive load with writing ability via schema construction, imagery processing, and emotional resonance, with Monte Carlo 95% confidence intervals (confirmatory); (C) grade-specific associations of intrinsic, extraneous, and germane load (exploratory); (D) indirect association via schema construction across teaching-quality levels and the index of moderated mediation. Error bars in (D) show ± 1 SE; ** p < .01, *** p < .001.

#### 4.3.5. Random-intercept cross-lagged panel analysis: within-person lagged associations between cognitive load and writing ability.

A random-intercept cross-lagged panel model (RI-CLPM) [41] was fitted to the four-wave data; because it absorbs stable between-student differences into random intercepts, its cross-lagged parameters reflect within-person temporal dynamics rather than causal effects. The lagged GCL→Writing path was significant at every lag (Table 7), strongest at T1 → T2 (β = 0.28, p < .001), weaker across the winter break (T2 → T3, β = 0.19, p < .001), and partially rebounding at T3 → T4 (β = 0.24, p < .001), with a smaller reverse path (mean β = 0.13, p = .004). The ECL→Writing path was consistently negative (mean β = −0.21, p < .001) with a non-significant reverse path (mean β = −0.07, p = .17), and ICL showed a similar pattern (mean β = −0.16, p = .002). This within-person lagged association pattern is consistent with, but does not by itself establish, the ordering assumed in the main and mediation models (Section 4.6.6).

**Table 7:** Cross-lagged parameter estimates from the RI-CLPM (standardized β).

Path	T1 to T2	T2 to T3	T3 to T4	Mean
GCL to Writing	0.28***	0.19***	0.24***	0.24***
Writing to GCL	0.16**	0.09*	0.14**	0.13**
ECL to Writing	−0.24***	−0.16***	−0.22***	−0.21***
Writing to ECL	−0.09	−0.03	−0.08	−0.07
ICL to Writing	−0.19**	−0.12*	−0.16**	−0.16**
Writing to ICL	−0.06	−0.02	−0.04	−0.04

Standardized within-person cross-lagged coefficients from the RI-CLPM. Model fit: χ²(39) = 164.6, CFI = 0.98, TLI = 0.94, RMSEA = 0.041, SRMR = 0.038. *** p < .001, ** p < .01, * p < .05.

### 4.4. Mediation effects and modeling objective validation

#### 4.4.1. Mediation effect analysis.

The multilevel mediation model supports statistical indirect associations of schema construction (ab = 0.242, 95% CI [0.179, 0.306]), imagery processing (ab = 0.189, 95% CI [0.132, 0.248]), and emotional resonance (ab = 0.156, 95% CI [0.104, 0.212]) from cognitive load to writing ability (Fig 10B), with a total indirect effect of 0.587 accounting for 76.2% of the total association. These indirect effects are interpreted as statistical indirect associations under the hypothesized ordering rather than as demonstrated causal mediation (Section 4.6.6).

Grade-specific analysis reveals a developmental shift (Fig 10C): the negative association of intrinsic load weakens from −0.49 in Grade 3 to −0.31 in Grade 6, the extraneous-load association peaks in Grade 4 (−0.55), and the positive germane-load association grows from 0.41 to 0.54.

#### 4.4.2. Moderated mediation model.

Teaching quality moderates the schema-based indirect association (Fig 10D), which was 0.30 (SE = 0.04, p < .001) in high-quality and 0.17 (SE = 0.05, p < .01) in low-quality teaching environments. The moderated-mediation index was 0.07 (95% CI [0.03, 0.11]) and was classified as stable in the small-cluster sensitivity analysis (Section 4.6.5): its sign was unchanged in all 12 leave-one-school-out refits (range [0.04, 0.10]), all its intervals excluded zero, and the simulated coverage of its Monte Carlo interval was 0.930.

### 4.5 Prediction model construction and validation

#### 4.5.1. Predictive performance under three validation designs.

Predictive performance was evaluated under the three validation designs specified in Section 3.6, each corresponding to a different prediction target that fixes the level of out-of-sample generalization it supports, with all imputation, centering, model reselection, refitting, BLUP computation, band-cutoff derivation, and leave-one-covariate-out feature-importance calculations performed within the training folds of each design (Supplemental Table S7 in S1 File) and random-effect predictions following Equations (19) to (21). The class-BLUP and school-BLUP components of Equations (19) and (20) are computed from the training-fold observations of the corresponding class or school, so no BLUP that enters a prediction draws on the outcomes of the validation students being predicted.

As shown in Table 8, Design A, the confirmatory predictive criterion of O6, meets the O6 RMSE threshold, with $R^{\text{2}}$ = 0.847 (95% CI [0.834, 0.859]) and RMSE = 4.28 points on the 0–100 writing-ability scale, whereas its three-band classification accuracy of 80.3% falls 4.7 percentage points short of the preset 85% target. Misclassifications are confined almost entirely to adjacent bands (Low-High confusions 0.2%), and the band-specific AUC for identifying low-band students is 0.953 (Supplemental Table S8 in S1 File), with band cutoffs and random-effect predictions drawing on no information from the validation students. Design B attains $R^{\text{2}}$ = 0.768 and RMSE = 5.24 and Design C attains $R^{\text{2}}$ = 0.656 and RMSE = 6.38, because the BLUPs of the class and school random effects drop out of the prediction equation stepwise; Designs B and C are reported without acceptance thresholds. Fig 11 contrasts the three designs and shows the feature importance obtained under Design A.

**Table 8:** Predictive performance under three validation designs.

Design	Prediction target	Folds	$R^{2}$	RMSE	MAE	Calibration slope	Three-band accuracy	Macro AUC
A. Student-grouped 10-fold CV (grade-balanced)	New students within sampled classes and schools	10	0.847 [0.834, 0.859]	4.28	3.02	1.01	80.3%	0.918
B. Leave-one-class-out CV	Students in unseen classes within sampled schools	48	0.768	5.24	3.71	0.96	73.8%	0.874
C. Leave-one-school-out CV	Students in unseen schools	12	0.656	6.38	4.55	0.94	66.9%	0.812

Design A serves as the confirmatory predictive criterion of O6, with the acceptance thresholds preset in Section 2.5 (RMSE ≤ 5.0 points on the 0–100 writing scale; three-band accuracy ≥ 85%), and its $R^{\text{2}}$ 95% CI is obtained from 1,000 student-level bootstrap resamples. Designs B and C report pooled out-of-fold performance without acceptance thresholds. Fold-level metrics of Design A, class-level metrics of Design B, and school-level metrics of Design C are provided in Supplemental Table S8 in S1 File. Predictions from the 20 imputed datasets were averaged within each fold.

**Fig 11:**
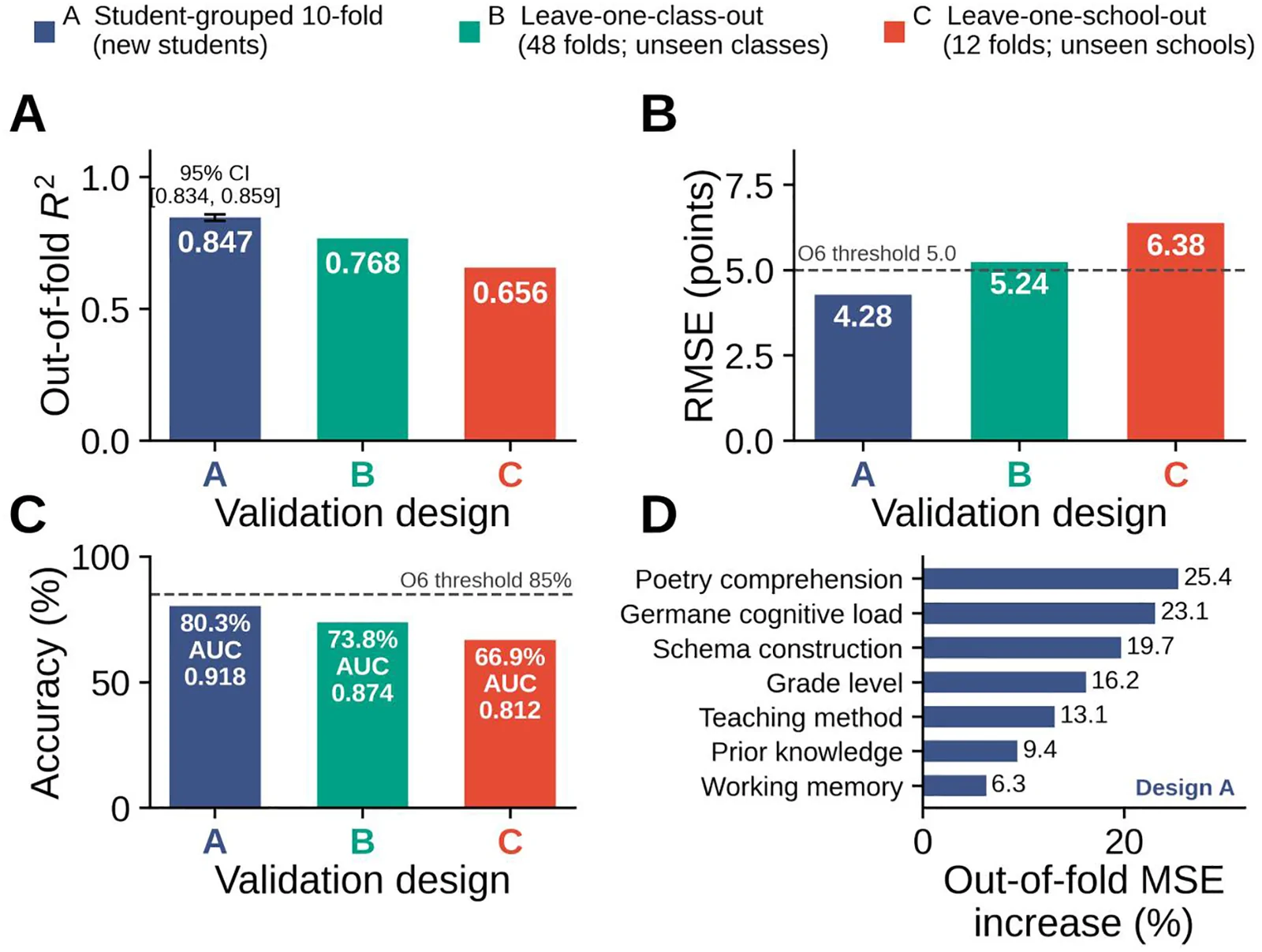
Predictive performance under three validation designs and feature importance. (A) out-of-fold $R^{\text{2}}$ for Design A (student-grouped 10-fold cross-validation, new students), Design B (leave-one-class-out, unseen classes), and Design C (leave-one-school-out, unseen schools), with the 95% student-level bootstrap interval for Design A; (B) RMSE, with the O6 acceptance threshold of 5.0 points that applies to Design A; (C) three-band classification accuracy at grade-normed 30th and 70th percentile cutoffs, with the macro-averaged ROC-AUC shown within each bar and the O6 acceptance threshold of 85% that applies to Design A; (D) leave-one-covariate-out feature importance under Design A, expressed as the percentage increase in out-of-fold MSE when the covariate is removed.

#### 4.5.2. Modeling objective validation.

Feature importance (Fig 11D) is the percentage increase in out-of-fold MSE when a covariate is removed from the model refitted in the training folds; because the covariates are correlated, the values are marginal and do not sum to 100%. Poetry comprehension ranks first (25.4%), followed by germane cognitive load (23.1%), schema construction (19.7%), grade level (16.2%), teaching method (13.1%), prior knowledge (9.4%), and working memory (6.3%) (Table 9), and the rank order is preserved under Design B (22.4%, 20.5%, 17.3%) and Design C (18.2%, 16.5%, 14.0%).

**Table 9:** Summary of model path estimation results against modeling objectives (O1 to O6), with inferential tier and multiplicity-adjusted p values.

Objective	Path	Estimate	SE	95% CI	Raw p	Tier	Adjusted p	Result
O1	ICL→Writing Ability	−0.41	0.08	[-0.57, -0.25]	<.001	Confirmatory (Holm)	<.001	Supported
	ECL→Writing Ability	−0.49	0.09	[-0.67, -0.31]	<.001	Confirmatory (Holm)	<.001	Supported
	GCL→Writing Ability	0.45	0.07	[0.31, 0.59]	<.001	Confirmatory (Holm)	<.001	Supported
O2	Poetry–Working Memory	0.38	0.06	[0.26, 0.50]	<.001	Exploratory (BH)	<.001	Supported
	Poetry–Prior Knowledge	0.41	0.07	[0.27, 0.55]	<.001	Exploratory (BH)	<.001	Supported
O3	Indirect via Schema	0.242	0.032	[0.179, 0.306]	<.001	Confirmatory (Holm)	<.001	Supported
	Indirect via Imagery	0.189	0.029	[0.132, 0.248]	<.001	Confirmatory (Holm)	<.001	Supported
	Indirect via Emotion	0.156	0.028	[0.104, 0.212]	<.001	Confirmatory (Holm)	<.001	Supported
O4	WM→Writing	0.31	0.05	[0.21, 0.41]	<.001	Exploratory (BH)	<.001	Supported
	PK→Writing	0.35	0.05	[0.25, 0.45]	<.001	Exploratory (BH)	<.001	Supported
O5	Teaching×CogLoad	0.47	0.17	[0.12, 0.82]	.011	Confirmatory (Holm)	.011	Supported
	Grade×CogLoad	0.39	0.16	[0.07, 0.71]	.019	Exploratory (BH)	.023	Supported
	Environment×CogLoad	0.31	0.14	[0.02, 0.60]	.038	Exploratory (BH)	.038	Supported
O6	Quadratic Growth (fixed)	−0.17	0.04	[-0.26, -0.08]	<.001	Confirmatory (Holm)	<.001	Supported
	Predictive accuracy under Design A	–	–	$R^{\text{2}}$ 0.847 [0.834, 0.859]; RMSE 4.28; accuracy 80.3%	–	Confirmatory (predictive)	–	Partially supported (RMSE met; accuracy below 85%)
	Random Intercept Var (class)	28.42	–	[18.11, 47.62]	<.001	Variance component	–	Supported
	Random Slope Var (student)	4.68	–	[3.32, 6.59]	<.001	Variance component	–	Supported

Standardized path coefficients are interpreted using effect-size benchmarks for multilevel path analysis (Preacher and Kelley [42]): |β | < 0.10 small, 0.10 ≤ |β | < 0.30 medium, |β | ≥ 0.30 large. The Holm procedure was applied to the eight confirmatory tests as one family, and the Benjamini-Hochberg procedure to the exploratory tests within their family. Variance components are reported with 95% profile-likelihood intervals and boundary-corrected likelihood-ratio p values and are not part of the multiplicity families.

All eight confirmatory tests retain significance under Holm familywise error control, so O1, O3, the primary O5 test, and the quadratic growth term of O6 meet their preset acceptance thresholds; the O6 predictive criterion is met for RMSE (4.28 against the 5.0-point threshold) but not for three-band accuracy (80.3% against the 85% target), so O6 is partially supported. The exploratory tests (O2, O4, the Grade × CogLoad and Environment × CogLoad interactions) remain significant under Benjamini-Hochberg control within their own family; they are reported as hypothesis-generating extensions to be re-examined before informing instructional recommendations. Random-effect variance components are reported as estimates rather than as tests within the multiplicity families.

#### 4.5.3. Non-significant and directionally unexpected findings during model building.

Non-significant and directionally unexpected results are documented as exploratory analyses under Benjamini-Hochberg control within their own family. (1) The ICL→Vocabulary Richness path (β = −0.09, BH-adjusted p = 0.11), the School Type × Grade interaction on intercepts (β = 0.14, BH-adjusted p = 0.23), and the Emotional Resonance × ECL interaction (β = 0.10, BH-adjusted p = 0.18) were non-significant. (2) The Grade 4 subgroup showed the strongest negative ECL association with writing (−0.55; bootstrap 95% CI [−0.71, −0.39]), possibly reflecting the introduction of regulated verse in Grade 4; this pattern is not used to support instructional recommendations, consistent with the exploratory status of the Grade × CogLoad interaction in Section 4.6.5. (3) An urban–rural difference in the mediation index was non-significant (β = 0.03, BH-adjusted p = 0.29), and a planned prosodic-fluency mediator was dropped after an unstable pilot factor structure (Cronbach’s α = 0.62).

### 4.6. Sensitivity analysis and robustness testing

#### 4.6.1. Missing-data and estimation robustness.

Across the four waves, 3.6% of writing scores and 1.9% to 3.8% of predictor values were missing, mainly because of absence on assessment days, leaving 7,128 complete person-wave observations (92.8%) for the complete-case analysis. Parameter estimates under the original model, complete-case analysis, multiple imputation, and Huber M-estimation differed by no more than 5% (Table 10), indicating insensitivity to missing-data handling and to influential observations.

**Table 10:** Robustness testing results.

Analysis Method	β(CogLoad)	β(Poetry)	β(Grade)	Model Fit
Original Model	−2.49***	0.51***	3.93***	AIC = 46206
Complete Case	−2.41***	0.49***	4.00***	AIC = 42897
Multiple Imputation	−2.51***	0.52***	3.91***	AIC = 46203
Robust Estimation	−2.46***	0.50***	3.96***	–
Bootstrap (n = 1000)	−2.50***	0.51***	3.94***	–
95% Bootstrap CI	[-2.88,-2.12]	[0.39,0.63]	[3.20,4.68]	–

*** p < .001. AIC values are comparable only among analyses of the same 7,680 observations; the complete-case analysis uses the 7,128 complete observations, the multiple-imputation AIC is the mean over the 20 imputed datasets, and Huber M-estimation does not define a likelihood-based AIC.

#### 4.6.2. Outlier detection under robust estimation.

After removing observations with Cook’s distance > 4/N, the main parameters changed within acceptable bounds, bootstrap 95% CIs (1,000 resamples) agreed with normal-theory intervals, and results were stable across group-mean and grand-mean centering.

#### 4.6.3. Comparative model fit and centering strategy.

Comparative model fitting supports the theoretical specification: the full mediation-and-moderation model outperforms a main-effects-only alternative (Δχ² = 326.45, df = 18, p < .001); the quadratic growth model outperforms a linear alternative (ΔAIC = 13.12); and the four-level specification is retained over a three-level alternative by the boundary-corrected likelihood-ratio test (${\overset{―}{\chi }}^{\text{2}}$ = 7.9, p = .034) and on design grounds, although AIC barely separates the two models (ΔAIC = 1.9) and BIC, whose penalty is computed on the 7,680 observations rather than on the 12 schools, favours the three-level model (ΔBIC = −18.9).

#### 4.6.4. Sensitivity to the cognitive load instrumentation.

The interpretive mapping from adapted NASA-TLX subscales to Sweller’s ICL/ECL/GCL distinction (Section 3.3) was examined in four complementary analyses. Convergent validity with the Klepsch et al. three-factor scale (Chinese adaptation) [25] was assessed on a stratified 20% subsample (n = 384) at T3: disattenuated correlations between corresponding constructs were r = 0.69 (ICL, 95% CI [0.63, 0.75]), r = 0.73 (ECL, [0.67, 0.78]), and r = 0.71 (GCL, [0.65, 0.77]), all p < .001. Klepsch-based re-estimation preserved the signs of the three load effects (ICL β = −0.37, ECL β = −0.47, GCL β = 0.48 versus −0.41, −0.49, 0.45), each within 0.05 of its NASA-TLX counterpart, and yielded a mediation index (0.56, 95% CI [0.47, 0.65]) indistinguishable from 0.587.

Three further analyses examined the mapping directly. The three-factor TLX model fit best at every wave (pooled: $\chi^{\text{2}}$ /df = 2.35, CFI = 0.960, TLI = 0.950, RMSEA = 0.048, SRMR = 0.042), outperforming the two-factor model (Δ CFI = 0.028, Δ RMSEA = −0.021) and the one-factor model (Δ CFI = 0.067, Δ RMSEA = −0.045), although the two-factor solution was acceptable (CFI = 0.932, RMSEA = 0.069). Configural, metric, and scalar invariance across Grades 3–6 held (Δ CFI = 0.004 and 0.007). In a multitrait-multimethod analysis, the average monotrait-heteromethod correlation of 0.71 exceeded the average heterotrait-heteromethod correlation of 0.28, and every monotrait-heteromethod correlation exceeded its heterotrait-monomethod counterparts. A single undifferentiated load score produced a poorer fit (Δ AIC = 68.4) and no cross-level interaction with teaching method (γ = 0.09, p = .53), so the O5 finding depends on the differentiation among the three load types (Supplemental Table S6 in S1 File); results that depend on the ICL, ECL, and GCL distinction are nevertheless interpreted as preliminary.

#### 4.6.5. Small-cluster sensitivity for Level-4 and cross-level estimates.

Because the highest level comprises only 12 schools, the general robustness of Bayesian estimation to small higher-level cluster counts is by itself insufficient to establish the stability of the Level-4 variance component and of the school-level and cross-level coefficients in the present dataset, and each of these estimates was therefore examined with the five analyses specified in Section 3.5. The five analyses target complementary aspects of small-cluster inference: 12 leave-one-school-out refits quantify the influence of each individual school on each estimate, the school-level wild cluster bootstrap with Webb weights (a school-level case bootstrap for the variance component and $ICC_{school}$) adjusts the interval width for the small number of clusters without relying on asymptotic Wald standard errors, the three alternative priors on the random-effect standard deviations characterize the prior sensitivity of the Bayesian re-estimation, the school fixed-effects specification removes the between-school variance component entirely for the coefficients that vary within schools, and the calibrated Monte Carlo simulation generates 1,000 datasets from the fitted model with the observed 12-school design and returns the empirical bias, sampling variability, and interval coverage that the inference procedures actually deliver under that design. Table 11 collects the six principal estimates, and Fig 12 displays the leave-one-school-out range of each estimate together with its Kenward-Roger, profile-likelihood, or Monte Carlo interval and its bootstrap interval, scaled to the main estimate.

**Table 11:** Small-cluster sensitivity analysis for Level-4 and cross-level estimates.

Estimate	Main	LOSO range	Most influential school	KR / profile 95% CI	Wild or school-level bootstrap 95% CI	Prior sensitivity (min, max)	School FE (Δ from main)	KR coverage	Status
$\tau_{v_{000}}$ (school-level intercept variance)	10.03	[6.21, 14.88]	School 07 (suburban)	[0.00, 38.69]	[0.00, 20.28]	7.21, 12.18	n/a (removed)	0.959	Imprecise
$ICC_{school}$	0.083	[0.053, 0.118]	School 07	[0.000, 0.260]	[0.000, 0.160]	0.061, 0.099	n/a	0.951	Imprecise
Teaching × CogLoad ($\gamma_{110}$)	0.47	[0.39, 0.56]	School 03 (urban)	[0.12, 0.82]	[0.11, 0.83]	0.10, 0.84 (all 4 priors exclude 0)	0.46 (Δ = −0.01)	0.945	Stable
Grade × CogLoad ($\gamma_{210}$)	0.39	[0.23, 0.52]	School 09 (rural)	[0.07, 0.71]	[-0.03, 0.80]	−0.01, 0.80 (3 of 4 priors exclude 0)	0.37 (Δ = −0.02)	0.912	Exploratory
Environment × CogLoad ($\gamma_{310}$)	0.31	[0.15, 0.43]	School 05 (suburban)	[0.02, 0.60]	[-0.06, 0.66]	−0.03, 0.66 (2 of 4 priors exclude 0)	0.27 (Δ = −0.04)	0.898	Exploratory
Index of moderated mediation	0.07	[0.04, 0.10]	School 11 (rural)	[0.03, 0.11]	[0.02, 0.12]	0.03, 0.11	0.06 (Δ = −0.01)	0.930	Stable

A coefficient is classified as Stable when its sign is unchanged in all 12 leave-one-school-out refits, its Kenward-Roger (KR) and wild-bootstrap 95% intervals exclude zero, its 95% credible interval excludes zero under all four priors (reference Inv-Gamma on the variances plus half-Cauchy(0, 5), half-Student-t(3, 0, 10), and Uniform(0, 30) on the random-effect standard deviations), and the simulated coverage of its KR interval is at least 0.925; otherwise it is marked Exploratory, and a variance component or ICC whose intervals reach the boundary of the parameter space is marked Imprecise. For $\tau_{v_{000}}$ and $ICC_{school}$ the interval columns report the profile-likelihood interval and a school-level case bootstrap interval and the coverage column reports the simulated coverage of the profile-likelihood interval; for the moderated-mediation index the second column reports its Monte Carlo interval. The school fixed-effects model checks only within-school-invariant coefficients. School identifiers are pseudonymous.

**Fig 12:**
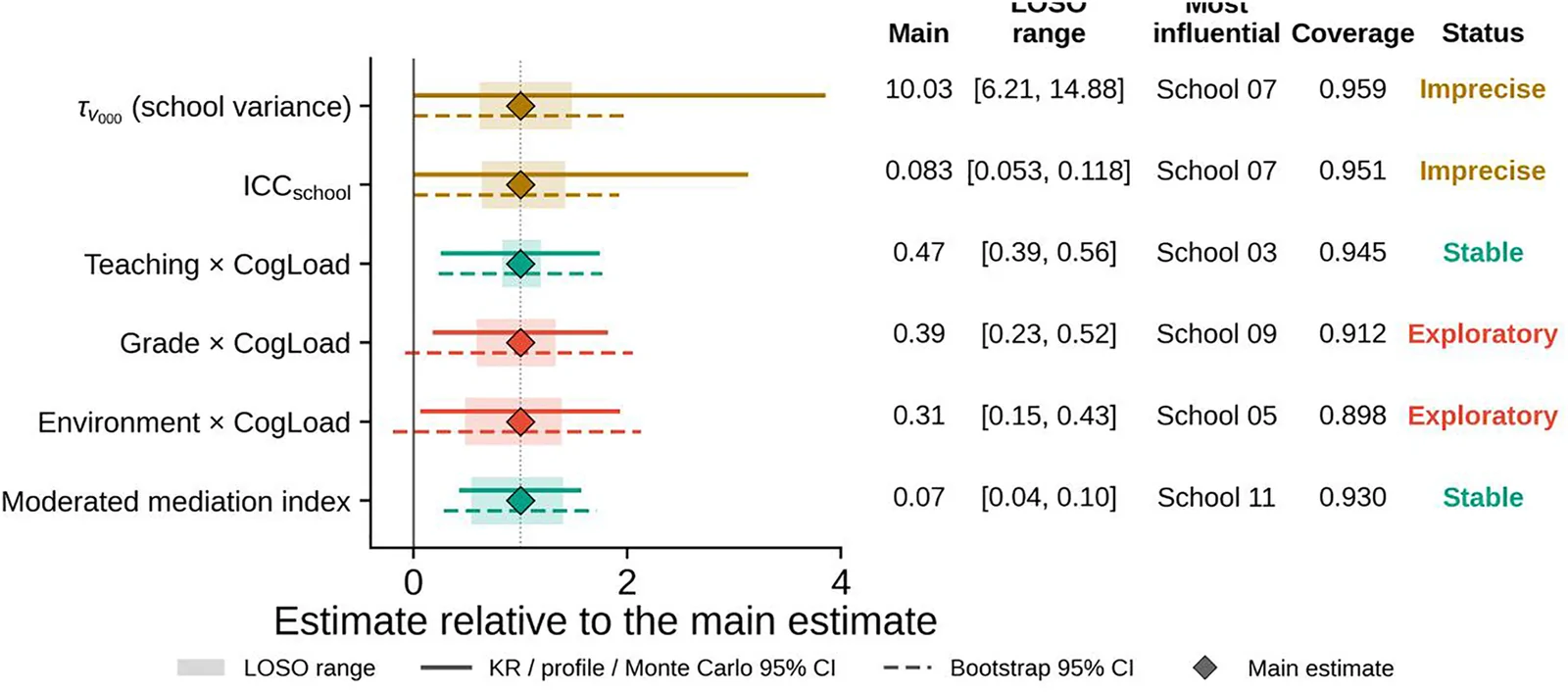
Small-cluster sensitivity of Level-4 and cross-level estimates.

For each estimate in Table 11, the diamond marks the main estimate and the shaded bar spans the 12 leave-one-school-out (LOSO) estimates; the solid interval is the Kenward-Roger 95% interval for coefficients, the Monte Carlo interval for the moderated-mediation index, or the profile-likelihood 95% interval for the variance component and $ICC_{school}$, and the dashed interval is the school-level wild cluster bootstrap 95% interval (school-level case bootstrap for the variance component and $ICC_{school}$). All quantities are divided by the main estimate, so 1 marks the main estimate and 0 marks a change of sign or, for the variance component and $ICC_{school}$, the boundary of the parameter space. The columns on the right list the main estimate, the LOSO range, the school whose omission changes the estimate most, the simulated coverage of the nominal 95% interval, and the stability classification.

As shown in Table 11 and Fig 12, the Teaching × CogLoad interaction and the moderated-mediation index meet all five stability criteria: their signs are unchanged in the 12 leave-one-school-out refits, their Kenward-Roger or Monte Carlo and wild-bootstrap 95% intervals exclude zero, their 95% credible intervals exclude zero under all four priors, and the calibrated Monte Carlo simulation returns nominal-95% coverage of at least 0.925 and relative bias within ± 3% (Table 11 and Supplemental Table S12 in S1 File). These two estimates therefore remain interpretable under the constraint that the highest level of the design contains 12 schools. The school-level intercept variance and $ICC_{school}$ remain positive in every leave-one-school-out refit (ranges [6.21, 14.88] and [0.053, 0.118]) and the boundary-corrected likelihood-ratio test supports a non-zero school variance (p = .030), but their profile-likelihood and school-level bootstrap intervals reach the boundary of the parameter space, the lower limits of their credible intervals approach zero under every prior, and the simulation shows that the school-level case bootstrap undercovers (0.818) while the profile-likelihood interval is close to nominal (0.959). Both are therefore marked Imprecise, the magnitude of school-level clustering is not interpreted, and the school level is retained to reflect the sampling design and to protect the standard errors of the class-level and cross-level coefficients. The Grade × CogLoad and Environment × CogLoad interactions fail the wild-bootstrap and coverage criteria and are prior-sensitive, so they are marked Exploratory in Table 9 and are not used as support for instructional recommendations at Level 4. The analytic registry in Supplemental Table S9 in S1 File records the pre-specification of the stability criteria and the placement of every Level-4 and cross-level estimate on the confirmatory or exploratory track. Complete leave-one-school-out estimates, the prior-sensitivity results, and the results of the calibrated simulation are reported in Supplemental Tables S10, S11, and S12 in S1 File, respectively.

#### 4.6.6. Sensitivity of the within-person lagged pattern and of the mediation ordering.

Because the ordering assumed in the main and mediation models is not established by the observational design, the RI-CLPM and the mediation analysis were re-examined with the three analyses specified in Section 3.5.

The within-person lagged pattern is robust to the added covariates (Table 12): every mean cross-lagged coefficient retains its sign, with reductions of at most 0.03. The temporally ordered indirect effects preserve the cross-sectional ranking, with schema construction largest (mean ab = 0.152, 95% CI [0.106, 0.198]), followed by imagery processing (0.121, [0.080, 0.163]) and emotional resonance (0.099, [0.059, 0.139]), each smaller than its cross-sectional counterpart as expected for lagged designs. The residual correlations that would eliminate them (0.21, 0.18, and 0.14) exceed the residual correlation of every observed covariate with the mediator or outcome equations (maximum 0.09 for teaching method), so an unmeasured confounder would need a stronger residual association than any covariate examined here to overturn them. The full covariate-adjusted RI-CLPM path table and the exact residual-correlation thresholds are reported in Supplemental Table S13 in S1 File.

**Table 12:** Sensitivity of the within-person lagged pattern to time-invariant and time-varying covariates.

Path	RI-CLPM main (mean β)	Covariate-adjusted (mean β)	Δ from main	Interpretation
GCL → Writing	0.24***	0.21***	−0.03	Robust
ECL → Writing	−0.21***	−0.19***	+0.02	Robust
ICL → Writing	−0.16**	−0.13**	+0.03	Robust
Writing → GCL	0.13**	0.11**	−0.02	Attenuated but retained
Writing → ECL	−0.07	−0.06	+0.01	Non-significant in both
Writing → ICL	−0.04	−0.03	+0.01	Non-significant in both

Covariate-adjusted RI-CLPM controls for grade, gender, school location, and teaching method as time-invariant covariates and for poetry comprehension as a time-varying covariate. Standardized within-person cross-lagged coefficients averaged across T1 to T2, T2 to T3, and T3 to T4. *** p < .001, ** p < .01, * p < .05.

## 5. Discussion

### 5.1. Main findings and their interpretation

The four confirmatory hypotheses (O1, O3, O5, and the quadratic growth term of O6) were supported under Holm familywise error control (Table 9), and their pattern is consistent with the within-person lagged associations of the RI-CLPM analysis (Section 4.3.5), whereas the O6 predictive criterion was met for RMSE but not for three-band accuracy (80.3% against the preset 85%). The negative associations of intrinsic (β = −0.41) and extraneous (β = −0.49) cognitive load and the positive association of germane cognitive load (β = 0.45) reached medium or larger effect sizes and extend cognitive-load theory to the Chinese classical-poetry context, subject to the provisional NASA-TLX to ICL/ECL/GCL mapping documented in Sections 3.3 and 4.6.4. The coupling between poetry-comprehension processing and cognitive resources (working memory β = 0.38; prior knowledge β = 0.41) describes an association whose direction cannot be determined from these data and is reported as exploratory (Table 9). The three mediating pathways jointly carry 76.2% of the total effect, indicating that the depth of cognitive processing is the principal statistical bridge in these data between poetry learning and writing growth, a ranking preserved in the temporally ordered analysis of Section 4.6.6. Relative to recent poetry-learning studies, which evaluated short-term interventions on retention, comprehension, or motivation [3–5,20], and to writing-development models that did not include classical poetry processing [6,7], the present design links poetry-related cognitive load to one-year writing growth, separates class-level (23.5%) and school-level (8.3%, imprecisely estimated) variance, and quantifies how far predictions transfer to unseen classes and schools.

Interactive instruction is thus associated with a weaker coupling between cognitive load and writing outcomes, which stands in apparent tension with the Reynaert et al. finding that an immersive VR poetry intervention heightened motivation while impairing memory retention [20]; Section 5.2 reconciles the two findings.

The attenuation of the cognitive-load-writing association in interactive-teaching classes is larger in lower grades (Grade 3 γ = 0.56, Grade 4 γ = 0.52) than in upper grades (Grade 5 γ = 0.42, Grade 6 γ = 0.39), consistent with maturing self-regulation, although the Grade × CogLoad interaction is exploratory (Table 11), so this pattern is hypothesis-generating. The 23.5% class-level variance share indicates that instructional context accounts for a non-negligible share of the variance in writing development, the school-level ICC of 0.083 is positive in every leave-one-school-out refit but imprecisely estimated (Table 11), so its magnitude is not interpreted, and the mildly decelerating quadratic growth ($\pi_{\text{2}}$ = −0.17 points per wave², Holm-adjusted p < .001) with substantial random-slope variance indicates marked individual differences in growth rates.

### 5.2. Reconciling with the Reynaert et al. paradox

The weaker negative association between cognitive load and writing ability observed in interactive-teaching classes, a cross-level moderation estimate from observational data (γ = 0.47, Holm-adjusted p = .011), and the Reynaert et al. result that immersive VR poetry learning raised motivation while impairing memory retention [20] are complementary rather than contradictory. The VR condition was high in immersion and low in guidance, whereas the present interactive condition combined moderate immersion with teacher-scaffolded multimedia, structured questioning, and worked-example prompts, and high immersion without guidance is likely to exceed rather than redirect cognitive resources. Retention of poem content also depends on encoding-phase resources, whereas writing is a later generative task that draws on consolidated schemata. The two studies jointly suggest a quadratic immersion-outcome relationship, consistent with the prediction that generative processing is maximized when instructional design directs rather than merely stimulates attention [5], and the reconciled account yields a testable prediction: adding explicit teacher prompts and attention cues to the VR protocol should reduce its memory decrement.

### 5.3. Limitations

Beyond the constraints of a single-province sample and a one-year window, four limitations warrant acknowledgement. On measurement, the mapping from NASA-TLX subscales to Sweller’s ICL/ECL/GCL distinction is a provisional operationalization, and germane load is now regarded as the working-memory resources devoted to intrinsic load rather than as an independent additive load [1]; although Section 4.6.4 supports the separability and cross-grade stability of the three TLX-indexed indicators, definitive resolution requires dedicated comparative validation, and results that depend on the ICL, ECL, and GCL distinction are interpreted as preliminary. On design, although the RI-CLPM analysis, the temporally ordered mediation, and the residual-correlation thresholds (Sections 4.3.5 and 4.6.6) are consistent with the assumed ordering, the observational design cannot establish causal effects or rule out unmeasured confounders such as shifts in reading habits or parental involvement, so statistical indirect associations are reported throughout. On modeling, the highest level comprises only 12 schools, which limits precision at Level 4 and for cross-level estimates; the Teaching × CogLoad interaction and the moderated-mediation index are stable in Section 4.6.5, the school-level variance component and the school-level ICC are non-zero but imprecisely estimated, and the Grade × CogLoad and Environment × CogLoad interactions remain exploratory, while the Grade 4 ECL peak (Section 4.5.3) hints at grade-specific non-linearity. On interpretation, only the confirmatory findings tested with familywise error control (Table 9) meet the preset acceptance criteria and support the implications summarized in Table 13, and predictive performance is confirmatory only for new students within the sampled classes and schools (Table 8), where three-band accuracy fell short of the preset target, so predicted bands should inform monitoring rather than placement decisions.

**Table 13:** Instructional and curricular implications for each modeling objective (O1 to O6).

Objective	Key finding	Effect size	Theoretical significance	Curriculum implications
O1: Load-component response (confirmatory)	Three TLX-indexed load components exhibit statistically separable associations with writing ability	ICL: −0.41***; ECL: −0.49***; GCL: 0.45***	Consistent with Sweller’s tripartite framework in the Chinese classical poetry context, subject to the provisional TLX mapping	Reduce ECL by simplifying displays, stage ICL, and foster GCL through why-questions
O2: Poetry-processing coupling (exploratory)	Significant coupling of poetry processing with working memory and prior knowledge	WM: 0.38***; PK: 0.41***	Poetry engagement co-varies with larger cognitive resources	Hypothesis for intervention studies: classical poetry as a lever for cognitive-resource development
O3: Mediation pathways (confirmatory)	Three statistical indirect associations are significant; the schema channel is largest	Total indirect: 0.587 (76.2% of total association)	Consistent with transfer through processing depth (schema, imagery, emotion) rather than surface exposure	Activate schema, imagery, and emotion through structural questions, imagery prompts, and emotional inquiry
O4: Individual features (exploratory)	WM and PK yield significant unique contributions	WM: 0.31***; PK: 0.35***	Individual differences persist after controlling class and school context	Candidate practice for testing: brief working-memory and prior-knowledge screening at semester start
O5: Cross-level moderation (confirmatory for teaching method)	Interactive teaching is associated with a weaker negative load-writing association; grade differences in this moderation are exploratory	γ = 0.47 (Holm-adjusted p = .011); grade-specific estimates exploratory	Instructional method interacts with cognitive maturity	Use interactive, multimedia-supported instruction to limit extraneous load; test grade-specific sequencing before adoption
O6: Nonlinear growth and prediction (quadratic term confirmatory)	Quadratic growth with random intercept/slope is the minimum-AIC specification; prediction for new students meets the RMSE target but not the 85% band-accuracy target	Quadratic: −0.17***; RMSE 4.28; band accuracy 80.3%	Writing ability follows a typical learning-curve trajectory with mild deceleration	Front-load intensive support and add new stimuli after wave 3; use predicted scores to monitor growth rather than to assign students to bands

## 6. Conclusion

This one-year, four-wave study of 1,920 Grade 3–6 students across 48 classes in 12 primary schools used a four-level hierarchical growth model to characterize the association between classical-poetry cognitive load and writing-ability development. Four confirmatory hypotheses are supported under Holm familywise error control: the three TLX-indexed load components exhibit differentiated associations with writing ability, with germane load positively associated with next-wave writing gains in the within-person RI-CLPM analysis; schema construction, imagery processing, and emotional resonance jointly carry 76.2% of the total association, a ranking preserved by a temporally ordered mediation model whose confounding thresholds exceed the residual associations of every measured covariate; the interaction of cognitive load with teaching method is retained under the small-cluster sensitivity analyses, whereas the interactions with grade and class environment are exploratory; and growth follows a quadratic learning curve. For new students within the sampled classes and schools, the pipeline attains $R^{\text{2}}$ = 0.847 and RMSE = 4.28 (Design A), meeting the preset RMSE target, whereas its three-band accuracy of 80.3% falls short of the preset 85% target, and prediction for unseen classes ($R^{\text{2}}$ = 0.768) and schools ($R^{\text{2}}$ = 0.656) degrades as the BLUPs drop out. Poetry comprehension, germane load, and schema construction are the most important predictors, and the confirmatory results extend cognitive-load theory to Chinese classical-poetry learning, subject to the provisional load-type mapping.

Limitations include a single-province sample, an observational design that cannot establish causal effects, a highest level of only 12 schools that limits precision at Level 4 and for cross-level estimates, and a one-year window; randomized or quasi-experimental interventions that test the instructional implications of Table 13, designs with more schools that would sharpen Level-4 inference, and longer time spans are natural next steps. The practical guidance for instructional design, curriculum reform, and teacher training is restricted to the confirmatory findings that meet the preset acceptance criteria under Holm familywise error control.

## Supporting information

S1 File The Supplementary Material provide verifiable data and documentation on school-level sampling and stratification, the full item pools, scoring rubrics, rater-training protocols, and psychometric evidence for the three mediator instruments, the fold-wise leakage-controlled prediction pipeline and disaggregated predictive performance, and robustness/transparency analyses including LOSO, prior sensitivity, Monte Carlo simulation, and covariate-adjusted RI-CLPM. (DOCX)
