# Supplementary material for: Construction of a hierarchical linear regression prediction model for classical Chinese poetry writing ability development
Source: PLoS One. 2026 Oct 30;21(10):e0361497. doi: 10.1371/journal.pone.0361497 (PMC13722217; doi:10.1371/journal.pone.0361497)
Supplement: Supplementary file 1 [file pone.0361497.s002.pdf]

Peer Review Report

PLOS ONE — Manuscript PONE-D-26-25548

**Manuscript Title:**	Construction of a Hierarchical Linear Regression Prediction Model for Classical Chinese Poetry Writing Ability Development
**Authors:**	Peigen Shi, Lei Zheng
**Article Type:**	Research Article
**Reviewer Recommendation:**	MAJOR REVISION (see note on data-integrity items, p.1)

OVERALL RECOMMENDATION: MAJOR REVISION

The manuscript addresses a genuinely under-studied question — the cognitive-load mechanisms linking classical poetry instruction to writing-skill development — with an ambitious multilevel longitudinal design. The review below is organized in two parts. Section 2 covers issues of basic verifiability (documentation, citations, data availability) that I would encourage the editor to weigh independently of the authors' scientific response. Section 3 covers substantive concerns about measurement, analytic design, and interpretation that bear on whether the paper's central claims are adequately supported as written.

1. Summary of the Submission

The authors report a one-year, four-wave longitudinal study of 1,920 students (grades 3–6, 48 classes, 12 schools) examining how three components of cognitive load (intrinsic, extraneous, germane) predict classical-poetry-linked writing ability, using three-level hierarchical linear models (student/class/school). The paper reports differentiated effects of the three load types, mediation via schema construction, imagery processing and emotional resonance (total indirect effect = 0.537), a cross-level moderation by teaching method, a quadratic growth trajectory for writing ability, and a final "prediction model" said to achieve 88.6% accuracy. All six pre-registered modeling objectives (O1–O6) are reported as supported.

2. Major Concerns — Verifiability and Documentation

2.1 Ethics documentation is internally inconsistent

The submission's Ethics Statement field (page 3 of the cover materials) is entered as **"N/A"**, which under PLOS ONE policy should only be used when the study did *not* involve human participants. Yet the manuscript body states unambiguously that **"This study has been approved by the Ethics Committee of Heze University"** and references written informed consent. This is a direct contradiction that the editorial office will need resolved regardless of the scientific review: the authors must supply the IRB/ethics committee name, an approval number, and clarify the consent procedure used for minors (parental/guardian consent should be specified, not just student assent, given participants are grade 3–6 children).

2.2 Widespread mismatch between in-text citation numbers and the reference list

The reference list (Section "References") is alphabetized by first-author surname, but the in-text bracketed citation numbers do not appear to have been updated to match — the numbers instead follow what looks like an original by-order-of-appearance numbering. This is not a one-off typo; the pattern is consistent across at least twelve checked citations. A sample of the mismatches:

In-text number	What the in-text citation describes	Correct ref. (by content)	What that number actually cites in the list
[1]	Sweller et al., 20-year review of cognitive architecture/instructional design	[24]	[1] Boere et al., fNIRS multitasking
[2]	Sweller (2024), individual differences in CLT	[23]	[2] Cer, metacognitive strategy
[4]	Liu et al., schema-associative mnemonic (8-wk study, n=127)	[14]	[4] Cowan, working memory review
[5]	Wang et al., emotional design in multimedia poetry learning	[25]	[5] de Jong, CLT critique
[6]	Kim & Schatschneider, DIEW model (n=193)	[11]	[6] De Vita et al., working memory & writing
[9]	Cowan, working memory review	[4]	[9] Gosse et al., spelling/handwriting growth
[10]	Swanson & Berninger, working memory & writing skill	[22]	[10] Graham et al., meta-analysis

This needs a full, careful renumbering pass (or restoration of consistent citation-manager linking) before the reference list can be trusted; as it stands, essentially every in-text citation points to the wrong source, which undermines the traceability of every claim attributed to prior literature.

2.3 Data-availability link could not be verified

The Data Availability statement points to a GitHub repository (github.com/a18907052542-lang/Chinese-Poetry-Writing-Ability-Development). I was unable to locate this repository or its owner through search, and direct access was blocked. This may simply reflect a private, unindexed, or very recently created repository — but given PLOS ONE's requirement that the minimal dataset be openly and fully available at submission, I'd ask the editor/authors to confirm the repository is public, resolves correctly, and actually contains the raw or minimal dataset (not just code) underlying every reported analysis, including item-level scale responses, before this claim is accepted at face value.

2.4 The "prediction model" and Algorithm 1 are underspecified and partly inconsistent with the HLM described in Section 3.5

Section 3.5 specifies REML estimation for the core multilevel models, and Bayesian/MCMC estimation "for complex models." Algorithm 1 then introduces a full machine-learning-style pipeline (10-fold stratified cross-validation, multiple imputation with 20 chains, 4 HMC chains × 10,000 iterations, leave-one-covariate-out feature importance) that is not clearly reconciled with the earlier description. Several concrete questions the authors should answer:

- What specific algorithm generates the point predictions in Fig. 10A (linear mixed-model fixed effects + BLUPs, or a separate learner such as random forest/gradient boosting)? The manuscript's prose suggests the former, but the ROC/AUC classification analysis in Fig. 10C implies a categorical classifier, and the two are never formally connected.
- The Abstract and Conclusion report "88.6% accuracy," while Section 4.5 separately reports R^2 = .786 and RMSE = 4.82 for a continuous outcome. These are different quantities describing different modeling tasks (regression vs. three-band classification) and should not be conflated as a single headline number.
- No modeling software is named anywhere (R/lme4/nlme, Mplus, HLM7, SPSS, Python/statsmodels, etc.). This is necessary for reproducibility and for judging whether the described REML, Bayesian/MCMC, and cross-validation steps were actually run in the same pipeline.

2.5 Construct mapping for the cognitive-load instrument is not justified

Cognitive load is measured with "an adapted NASA-TLX scale" whose three subscales are labeled Mental Effort, Task Difficulty, and Performance/self-efficacy (the manuscript uses both labels inconsistently — compare Section 3.3 text to Fig. 5A). The NASA-TLX was designed as a general workload inventory, not as an operational measure of Sweller's tripartite intrinsic/extraneous/germane distinction, and "Performance" is conceptually an outcome rather than a load. The authors should justify, ideally with a citation or their own validation evidence beyond the reported CFA fit indices, why these three subscales should be interpreted as ICL, ECL, and GCL respectively, rather than as three general facets of perceived workload.

2.6 Uniformly confirmatory results across all six modeling objectives

Table 6 reports that all six a priori objectives (O1–O6), spanning 16 separate parameter estimates, are "Supported," the great majority at $p < .001$. This is an unusually clean pattern for an exploratory, multi-path multilevel model with this many free parameters, and it is worth the authors explicitly addressing: were any hypothesized paths, subgroup contrasts, or robustness checks non-significant or in an unexpected direction at any stage of model-building? A brief account of what did *not* work as expected (or a statement that truly nothing did) would strengthen confidence in the reported effects.

2.7 Framing of Table 8 ("Engineering contribution," "deployment pipeline") is misaligned with the journal and audience

Table 8 and the surrounding text describe findings using software/ML-engineering vocabulary — "fixed-effect priors reusable in downstream models," "regression anchors," "deployment pipeline," "grade-conditional gain table for adaptive model deployment." For a PLOS ONE education/psychology readership, this framing obscures rather than clarifies the pedagogical implications and reads as inconsistent with the rest of the paper's register. I'd recommend rewriting this table and the surrounding discussion in terms of instructional and curricular implications (e.g., what a classroom teacher or curriculum designer should change), which is presumably the intended contribution.

3. Major Concerns — Content, Measurement, and Analytic Validity

The items below concern the science itself: whether the instruments, design, and reported statistics can support the paper's claims as written, independent of the documentation issues in Section 2.

3.1 The three mediators are never operationalized in the Methods

Section 3.3 describes instruments for exactly three constructs: the Cognitive Load Scale, the Poetry Comprehension Test, and the Writing Assessment. Section 4.4 then reports full mediation results — including specific indirect effects (0.218, 0.175, 0.144) and Monte Carlo confidence intervals — for three additional constructs: schema construction, imagery processing, and emotional resonance. No scale, item count, reliability coefficient, or scoring procedure is given anywhere for these three mediators. This needs to be corrected before the mediation analysis (arguably the paper's central contribution, at 74.8% of total effect) can be evaluated at all.

3.2 Discriminant validity between predictors, mediators, and outcome is not demonstrated

Writing ability is scored on six dimensions including thematic coherence and creative expression (Fig. 5C); the mediators are schema construction, imagery, and emotion; and the poetry comprehension test spans literal/inferential/critical/creative levels (Fig. 5B). These instruments plausibly share conceptual and item content, which would mechanically inflate both the Table 3 correlations and the reported 74.8%-of-total-effect mediation — an unusually complete mediation for a psychological process. I'd ask the authors to report discriminant validity (e.g., HTMT ratios, or at minimum the inter-construct correlations from the measurement model) between the mediators, the poetry comprehension test, and the writing outcome, rather than the CFA fit indices for cognitive load and writing alone.

3.3 Causal language exceeds what the design supports

The models are concurrent/longitudinal mean-level associations, not cross-lagged panel models. That supports describing how cognitive load and writing ability co-develop over the year, but not the causal language used throughout ("cognitive load ... predicted writing ability," "GCL positively predicted it"). A plausible reverse pathway — weaker writers experiencing more load, or stronger poetry processors reporting less load — is never raised or ruled out. I'd recommend either a cross-lagged analysis establishing temporal precedence, or systematically softening the causal language to "was associated with" / "forecast."

3.4 Self-report cognitive-load measurement in grade 3–6 children

The adapted NASA-TLX is an introspective self-report instrument, collected here from children as young as ~8 years old. Whether children this age can reliably introspect on and differentiate intrinsic, extraneous, and germane load — a distinction that is subtle even for adult learners — is a construct-validity question the manuscript does not address, and it compounds the NASA-TLX-mapping concern raised in Section 2.5.

3.5 ICC / variance-decomposition formulas appear internally inconsistent

Equations (11)–(12) define the class and school ICCs using only τ_{00} (class intercept variance) and τ_{11} (defined in Section 3.5 as class-level *slope* variance) — the school-level variance term ψ_{00} from equation (5) never enters either formula. Yet Table 5's reported "school ICC" of 0.318 numerically matches $(\tau_{00}+\psi_{00})$/total rather than what equation (12) actually specifies. Either the printed formula is wrong or the notation in Section 3.5 and Table 5 do not correspond — this should be confirmed directly with the authors rather than assumed to be a typo.

3.6 Ambiguity between a three-level and a four-level model

Equation (1) already embeds Time and Time^2 at "Level 1 (student)," collapsing occasions into the student level, while the growth-curve section (eq. 8–10) treats time as effectively its own level. With four waves nested in students nested in classes nested in schools, this could reasonably be framed as a four-level model. It matters for interpretation: does the 68.2% "student-level" variance in Table 5 include within-student change over the year, or only stable between-student differences? The manuscript is not consistent enough on this point to tell.

3.7 Multicollinearity among the three cognitive-load predictors is unaddressed

Table 3 shows ICL–ECL r = .45 and ECL–GCL r = –.52 — the three predictors whose "differentiated" effects are central to the paper's contribution are moderately-to-strongly correlated with one another when entered simultaneously. No VIF, tolerance, or condition-index statistics are reported, which matters particularly here given the paper's core claim rests on cleanly separating the three effects.

3.8 Inter-rater reliability procedure for writing scores is underspecified

Table 4 gives a single inter-rater r = .88 for the entire 30-item, six-dimension writing assessment. The number of raters per composition, whether scoring was blind to time point/condition, and how disagreements were resolved are not described — standard information to require for a scored open-ended writing task underlying 7,680 observations.

3.9 Discussion does not engage with counter-evidence the paper itself cites

The Introduction cites Reynaert et al.'s finding that an engaging, high-immersion learning method paradoxically impaired memory performance despite raising motivation — directly relevant to this paper's own claim that interactive teaching buffers cognitive load's negative effects on writing — but the Discussion never returns to reconcile the two findings. The Limitations paragraph is also thin (single province, one academic year) relative to the range of concerns raised in this review, and would benefit from directly addressing measurement and design limitations rather than only generalizability.

4. Minor and Presentational Concerns

MINOR **Grade/location stratification description**

Section 3.2 stratifies schools into three tiers by "teaching quality, geographical location and school size" (4 schools/tier), but Table 1/Fig. 4 report a different tripartite breakdown (urban 33.3% / suburban 25% / rural 41.7%) without stating how the two stratification schemes relate. Please clarify.

MINOR **Grade-specific growth-rate percentages vs. the quadratic model**

The 12.3%/8.7%/6.2%/4.8% grade-3 "growth rates" reported after Table 2 are not clearly derived from the quadratic growth-curve parameters reported in Section 4.3 (π_0, π_1, π_2); please specify the formula used, or move this to a supplementary calculation.

MINOR **Copyediting and machine-translation artifacts**

Inconsistent capitalization of author names in-text ("sweller et al.," "nurullizam," "schatschneider"), inconsistent terminology ("quadratic" vs. "secondary" growth pattern used interchangeably), and several awkward constructions throughout suggest the manuscript has not had a final proofreading pass. A full language edit is needed before publication.

MINOR **IRB approval number absent**

Even once the Ethics Statement contradiction (Sec. 2.1) is resolved, the manuscript text itself should state the approval/reference number, not just the committee name.

MINOR **Effect-size benchmarking**

Multiple standardized path coefficients (~0.3–0.5) are described as "medium and above effect quantity"; please cite the specific benchmark used for interpreting HLM path coefficients (Cohen's conventions were developed for correlations/regression R^2, and their direct application to multilevel path coefficients should be justified or softened).

MINOR **Keyword inconsistency**

The keyword list differs between the submission metadata ("...Longitudinal study") and the in-manuscript abstract ("...Data science applications in education"); please reconcile.

5. Strengths Worth Preserving

- A genuinely large, well-powered nested sample (1,920 students, 48 classes, 12 schools) with four measurement waves — a scale that is uncommon in classical-poetry-education research and, if the data hold up under the checks above, would be a valuable contribution.
- Appropriate use of a three-level HLM framework for the genuinely nested data structure, with sensible centering strategy (CWC for Level-1, CGM for Level-2/3).
- The mediation and cross-level moderation analyses target a real and under-examined theoretical gap: how instructional context modulates the cognitive-load-to-writing pathway across developmental stages.
- Reported robustness checks (multiple imputation vs. complete case, bootstrap CIs, outlier-robust estimation) are a good practice and, once the modeling pipeline is clarified (Sec. 2.4), should be retained.

6. Checklist of Requested Revisions

■ 1. Resolve the Ethics Statement contradiction; provide IRB name, approval number, and parental/guardian consent details for minor participants.

- 2. Fully re-verify and correct every in-text citation number against the reference list (or vice versa).
- 3. Confirm the GitHub data repository is public, stable, and contains the full minimal dataset (not only code); provide a permanent identifier (e.g., DOI via Zenodo/OSF) if possible.
- 4. Clarify the exact algorithm(s) used to generate the "prediction model," reconcile the R^2/RMSE (regression) and accuracy/ROC-AUC (classification) results, and name the software/packages used throughout.
- 5. Justify the mapping from NASA-TLX subscales to ICL/ECL/GCL, or relabel the construct as general perceived workload if a stronger justification isn't available.
- 6. Add a brief, candid account of any non-significant or unexpected results encountered during model-building.
- 7. Rewrite Table 8 and the surrounding discussion in instructional/curricular terms rather than ML-deployment terms.
- 8. Fully describe the instruments (items, reliability, scoring) used to measure schema construction, imagery processing, and emotional resonance — currently undocumented despite anchoring the paper's central mediation result.
- 9. Report discriminant validity (e.g., HTMT or inter-construct correlations) between the mediators, the poetry comprehension test, and the writing outcome to rule out item-content overlap as a driver of the mediation effect.
- 10. Either add a cross-lagged/temporal-precedence analysis to support causal language, or revise "predicted"/"effect" language throughout to association-level language.
- 11. Address the plausibility of self-reported cognitive-load differentiation (ICL/ECL/GCL) in grade 3 children (~8 years old), or cite validation evidence for this age range.
- 12. Reconcile equations (11)–(12) (ICC formulas) with the variance components actually reported in Table 5.
- 13. Clarify whether the model is three-level or four-level given that Time/$Time^2$ are embedded at Level 1, and state clearly what the reported 68.2% "student-level" variance does and does not include.
- 14. Report VIF/tolerance statistics for ICL, ECL, and GCL given their moderate-to-strong pairwise correlations.
- 15. Describe the writing-assessment rating procedure (number of raters per composition, blinding, disagreement resolution).
- 16. Reconcile the Discussion with the Reynaert et al. finding cited in the Introduction, and expand the Limitations section to cover measurement and design limitations, not only sample generalizability.
- 17. Address the minor items in Section 4, and commission a full language/copyediting pass.

Note: Items 2.1–2.3 concern the verifiability of the manuscript's basic documentation, and items in Section 3 concern substantive measurement and analytic validity — both are treated as independently significant from each other. I would suggest the editor confirm the Section 2 items directly (e.g., with the institution's ethics committee and the data repository) alongside the standard author revision process for Section 3.
