# Supplementary material for: Construction of a hierarchical linear regression prediction model for classical Chinese poetry writing ability development
Source: PLoS One. 2026 Oct 30;21(10):e0361497. doi: 10.1371/journal.pone.0361497 (PMC13722217; doi:10.1371/journal.pone.0361497)
Supplement: Supplementary file 2 [file pone.0361497.s001.docx]

**Supplementary Materials**

*for the manuscript entitled*

**“Construction of a Hierarchical Linear Regression Prediction Model for Classical Chinese Poetry Writing Ability Development”**

**Contents**

Supplemental Table S1. Cross-tabulation of quality-tier stratification, geographic location, and per-grade cohort size for the 12 sampled primary schools.

Supplemental Appendix S2. Poetry Schema Construction Task (PSCT): item pool, scoring rubric, and rater training protocol.

Supplemental Appendix S3. Imagery Generation and Verification Task (IGVT): item pool, scoring rubric, and rater training protocol.

Supplemental Appendix S4. Poetry Emotional Resonance Scale (PERS): item pool, scoring rubric, and rater training protocol.

Supplemental Table S5. Summary of reliability and validity indices for the three mediator instruments across the four measurement waves.

Supplemental Table S6. Measurement analyses of the adapted NASA-TLX load-type mapping

Supplemental Table S7. Fold-wise analysis pipeline, leakage checklist, and specification selection across folds

Supplemental Table S8. Fold-level, class-level, and school-level predictive performance under the three validation designs

Supplemental Table S9. Analytic registry of confirmatory, exploratory, and sensitivity analyses

Supplemental Table S10. Leave-one-school-out estimates of Level-4 and cross-level parameters

Supplemental Table S11. Prior sensitivity of Level-4 and cross-level parameters

Supplemental Table S12. Calibrated Monte Carlo simulation of small-cluster inference

Supplemental Table S13. Covariate-adjusted RI-CLPM paths and residual-correlation thresholds for the temporally ordered indirect associations

# **Supplemental Table S1**

**Cross-tabulation of quality-tier stratification, geographic location, and per-grade cohort size for the 12 sampled primary schools**

This table provides the school-level cross-tabulation referenced in Section 3.2 of the main manuscript (paragraph beginning “In the first stage, primary schools in the province were classified into three composite tiers…”). The stratification factor is a composite quality tier derived from the provincial department of education’s annual assessment (Tier I = provincial demonstration school; Tier II = municipal key school; Tier III = standard neighbourhood school). The geographic-location descriptor (urban / suburban / rural) and the per-grade cohort size were retained as independent school-level covariates in the four-level growth model.

Composite quality-tier and geographic-location descriptors are approximately orthogonal (χ²(4) = 0.90, *p* = 0.925; Cramér’s *V* = 0.194), confirming that the tier stratification does not confound with the location covariate in the school-level equation of the growth model.

| **School ID** | **Tier** | **Geographic location** | **Per-grade cohort (Grades 1-6 avg)** | **Grade 3-6 cohort (×4)** | **Sampled classes** | **Sampled students** |
| --- | --- | --- | --- | --- | --- | --- |
| S01 | I | Urban | 175 | 700 | 4 | 160 |
| S02 | I | Urban | 165 | 660 | 4 | 160 |
| S03 | II | Urban | 155 | 620 | 4 | 160 |
| S04 | III | Urban | 145 | 580 | 4 | 160 |
| S05 | I | Suburban | 130 | 520 | 4 | 160 |
| S06 | II | Suburban | 120 | 480 | 4 | 160 |
| S07 | III | Suburban | 110 | 440 | 4 | 160 |
| S08 | I | Rural | 215 | 860 | 4 | 160 |
| S09 | II | Rural | 205 | 820 | 4 | 160 |
| S10 | II | Rural | 200 | 800 | 4 | 160 |
| S11 | III | Rural | 195 | 780 | 4 | 160 |
| S12 | III | Rural | 185 | 740 | 4 | 160 |
| **Urban subtotal** | 4 schools | 160.0 (avg) | 640.0 (avg) | 16 classes | 640 |  |
| **Suburban subtotal** | 3 schools | 120.0 (avg) | 480.0 (avg) | 12 classes | 480 |  |
| **Rural subtotal** | 5 schools | 200.0 (avg) | 800.0 (avg) | 20 classes | 800 |  |
| **Overall total** | **12 schools** | **166.7 (wtd)** | **666.7 (wtd)** | **48 classes** | **1,920** |  |

**Cross-tabulation of tier by geographic location (school counts, N = 12 schools)**

| **Quality tier** | **Urban** | **Suburban** | **Rural** | **Row total** |
| --- | --- | --- | --- | --- |
| Tier I (Provincial demonstration) | 2 | 1 | 1 | 4 |
| Tier II (Municipal key) | 1 | 1 | 2 | 4 |
| Tier III (Standard neighbourhood) | 1 | 1 | 2 | 4 |
| **Column total** | **4** | **3** | **5** | **12** |

***Note.*** At the student level, the sampled distribution weighted by 40 students per class × 4 grades per school reproduces the aggregate reported in Table 1 of the main manuscript: 640 urban (33.3%), 480 suburban (25.0%), and 800 rural (41.7%) students. Owing to expected cell frequencies below 5, Fisher’s exact test on the 3×3 cross-tabulation was also computed and produced a Freeman–Halton *p* = 0.916, converging with the Pearson chi-square result and supporting the assumption of tier–location orthogonality used in the school-level model.

Per-grade cohort figures are averaged across Grades 1–6 within each school and were obtained from the schools’ official 2024–2025 enrollment registries; the Grade 3–6 cohort column represents the sub-cohort from which sampling occurred and is exactly the per-grade cohort figure multiplied by four. In every school, one class per grade was randomly selected without replacement and 40 students whose guardians gave consent were randomly drawn from it (enrolled class sizes 41 to 51 students; Table 1 of the main manuscript), yielding a constant per-school sample of 160 students and a total sample of 1,920 students in 48 classes, consistent with Section 3.2 of the main text.

# **Supplemental Appendix S2**

**Poetry Schema Construction Task (PSCT): item pool, scoring rubric, and rater training protocol**

## **S2.1 Instrument overview**

The Poetry Schema Construction Task (PSCT) was developed to operationalise the schema-construction mediator between cognitive load and classical-Chinese-poetry writing ability. It comprises **6 open-response items** administered in three subtasks of 2 items each: (i) imagery-association mapping, (ii) theme-to-image alignment, and (iii) schema transfer to a novel poem. The construct is anchored in classical schema theory [Anderson, 1977, reference [1]] and Ausubel’s meaningful learning theory [Ausubel, 1968, reference [2]]. Total administration time is approximately 25 minutes per wave.

Psychometric summary across the four measurement waves (T1–T4, N = 1,920 within-wave observations): Cronbach’s *α* = 0.84 (95% CI: 0.82–0.86); McDonald’s *ω* = 0.85; two-rater ICC(2,1) = 0.87; test–retest reliability (T1–T4, 9-month) r = 0.81; confirmatory factor analysis of a one-factor model with three method (subtask) parcels yielded χ²/df = 2.31, CFI = 0.965, TLI = 0.949, RMSEA = 0.049 (90% CI: 0.038–0.061), SRMR = 0.041. Detailed values are provided in Supplemental Table S5.

## **S2.2 Theoretical foundation**

PSCT items were designed so that a correct response requires (a) activating an appropriate poetic schema from long-term memory, (b) elaborating that schema with a specific classical-Chinese-poetry exemplar, and (c) transferring the schema to a novel stimulus. This tripartite structure operationalises the three canonical schema functions of retrieval, elaboration, and generalisation, and follows Anderson’s [1] argument that schema construction is measurable only when learners are required to move beyond recognition of familiar exemplars to structured productive use. The theme-to-image alignment subtask further embeds Ausubel’s [2] meaningful-learning premise that new content must be anchored to a superordinate conceptual framework.

All six items were piloted in the summer of 2024 with 120 primary-school pupils (Grades 3–6) drawn from two schools that did not enter the main sample. Item-level classical-test-theory statistics guided the final selection: item difficulty (proportion at the top rubric band) ranged from 0.34 to 0.62; corrected item–total correlations ranged from 0.51 to 0.68; no item was flagged for differential-item functioning by grade or gender.

## **S2.3 Full item pool**

***Language note.*** *The original poem titles, excerpts, and Chinese-language terms in this appendix are in Chinese. English translations are provided here for reference only. The original Chinese versions should be used for administration and scoring; response-length limits refer to Chinese characters, not English words.*

### **Subtask 1 – Imagery-association mapping (Items PSCT-1 and PSCT-2)**

Instruction to student: “Read the couplet below. In two or three sentences, describe which recurring image the poet is using and what mood or idea that image conventionally carries in classical Chinese poetry. Try to give at least one other poem you have learned that uses the same image.”

**PSCT-1.** Stimulus couplet from *Quiet Night Thoughts* (Li Bai): “Lifting my head, I gaze at the bright moon; lowering my head, I think of home.”

- Target schema: bright moon → homesickness; canonical Tang-poem imagery in which the shared sky becomes a link between the traveller and the distant home.
- Expected exemplars (any one sufficient for full credit): Wang Anshi, “Mooring at Guazhou” (“When will the bright moon shine on my return?”), Du Fu, “Remembering My Younger Brothers on a Moonlit Night”, Su Shi, “Prelude to Water Melody” (“May we live long and share the moon’s beauty, though a thousand miles apart”).

**PSCT-2.** Stimulus couplet from *Sending Yuan Er on a Mission to Anxi* (Wang Wei): “Morning rain in Weicheng dampens the light dust; beside the guesthouse, the willows are fresh and green.”

- Target schema: willow / breaking a willow branch → parting; grounded in the Han–Tang folk custom of gifting a willow sprig to a departing friend (zhe, meaning “break”, rhymes with xi, meaning “cherish”).
- Expected exemplars: Wang Zhihuan, “Farewell” (“Willows stand in the east wind”), Li Bai, “Laolao Pavilion” (“The spring wind knows the sorrow of parting and will not let the willow branches turn green”), Bai Juyi, “Willows by the Green Gate”.

### **Subtask 2 – Theme-to-image alignment (Items PSCT-3 and PSCT-4)**

Instruction to student: “Read the four-line poem provided. In no more than 80 characters, explain how the poet uses the specific images in the poem to develop the overall theme. Your answer should name at least two images and connect each to the theme.”

**PSCT-3.** Stimulus poem: *Wang Zhihuan, “Ascending Stork Tower”: “The bright sun sinks behind the mountains; the Yellow River flows into the sea. To see a thousand miles farther, climb one more storey.”*

- Target theme: aspirational perseverance / striving for progress.
- Model alignment path: (i) setting-sun “bright sun” and the descending trajectory (“sinks behind the mountains”) establish a natural limit; (ii) the great-river-to-sea image “the Yellow River flows into the sea” provides horizontal extension; (iii) the imperative “climb one more storey” converts the spatial images into a moral admonition about extending one’s vantage through effort.

**PSCT-4.** Stimulus poem: *Liu Zongyuan, “River Snow”: “Across a thousand mountains, no birds fly; along ten thousand paths, no human trace remains. In a lone boat, an old man in a straw cape and bamboo hat fishes alone on a cold river in the snow.”*

- Target theme: solitary detachment amid political disappointment / aloof integrity and endurance of the cold.
- Model alignment path: (i) hyperbolic absence images (“a thousand mountains…no birds”, “ten thousand paths…no human trace”) erase the human world; (ii) the lone-fisherman image (“in a lone boat, an old man in a straw cape and bamboo hat”) centres the poem on a solitary subject; (iii) fishing-in-snow (“fishes alone on a cold river in the snow”) integrates the environmental and human images into a stance of composed resistance.

### **Subtask 3 – Schema transfer to a novel poem (Items PSCT-5 and PSCT-6)**

Instruction to student: “The poem below is one you have not encountered in class. In no more than 120 characters, (a) identify the schema (mood, situation, or theme) that the poem shares with a well-known poem you have studied, (b) name that well-known poem and its author, and (c) briefly justify the transfer by pointing to at least two comparable images across the two poems.”

**PSCT-5.** Novel stimulus poem (Grade-3/4 form): *Jia Dao, “Seeking a Hermit Without Finding Him”: “Beneath a pine, I ask a boy, who says his master has gone to gather herbs. He is somewhere on this mountain, but the deep clouds conceal his whereabouts.”*

- Target schema: reclusive solitude in a mountain landscape / withdrawal into mountain forests.
- Well-known transfer target (any one sufficient): Wang Wei, “An Autumn Evening in the Mountains”, Wang Wei, “Deer Enclosure”, or Meng Haoran, “Mooring on the Jiande River”.
- Comparable image pairs: pine ↔ pine; mountain ↔ mountain; clouds ↔ mist / bamboo grove; child-informant ↔ fisher-informant.

**PSCT-6.** Novel stimulus poem (Grade-5/6 form): *Liu Yuxi, “Autumn Verses”: “Since ancient times, autumn has brought sorrow and loneliness; I say an autumn day surpasses a spring morning. A lone crane soars through the clouds into a clear sky, drawing my poetic inspiration up to the blue heavens.”*

- Target schema: revaluation of an autumn convention / rejecting autumnal melancholy.
- Well-known transfer target (any one sufficient): Wang Zhihuan, “Ascending Stork Tower” (upward gaze), Chen Ziang, “Song on Ascending Youzhou Terrace” (temporal defiance), or Liu Yuxi, “In Reply to Letian’s Poem Presented at Our First Banquet Meeting in Yangzhou” (revival amid decline).
- Comparable image pairs: rising crane “a lone crane soars through the clouds” ↔ climbing higher “climb one more storey”; sky as space of aspiration “blue heavens” ↔ vast horizon “a thousand miles”.

## **S2.4 Scoring rubric**

Each of the six items is scored on a common 0–4 primary-trait rubric. Scores are summed across items to produce a total score in the range 0–24; the mediator variable entered into the four-level growth model is the wave-specific standardised (z-scored) total, following the practice reported in the main text (Section 3.3, Table 4).

| **Band** | **Descriptor** | **Response evidence at this band** |
| --- | --- | --- |
| 4 | Fully constructed schema | Response (a) names the target schema in classical-poetry-appropriate vocabulary, (b) supplies an accurate exemplar or transfer target, and (c) articulates the image–theme (or image–image) relation with reference to at least two specific poem elements. No factual error. |
| 3 | Substantially constructed schema | Response contains all three components (schema, exemplar, articulation) but one is under-specified or contains a minor factual slip (e.g., correct schema but only one comparable image; correct poem but wrong author). |
| 2 | Partial schema | Two of the three components are present; typically the schema is correct and an exemplar is offered, but no image–theme articulation is attempted, or an articulation is offered but the exemplar is missing or non-canonical. |
| 1 | Emergent schema | Only one component is present, typically a plausible schema label without exemplar or articulation; or an articulation that describes surface features (“there is a moon”) without identifying the schema. |
| 0 | Absent or incorrect | Blank response, off-topic response, or response that misidentifies the schema (e.g., interpreting Quiet Night Thoughts as a frontier poem) with no salvageable content. |

Additional scoring conventions: (i) responses in colloquial modern Chinese are accepted; classical vocabulary is neither rewarded nor penalised so long as the schema label is unambiguous. (ii) A response that supplies a correct exemplar from outside the primary-school curriculum (e.g., a poem from a family reader) receives the same credit as a curricular exemplar. (iii) A response that transfers to a well-known poem with an inaccurate title but an unambiguous author is scored at Band 3 rather than Band 4. (iv) A response longer than the stated word limit is not penalised so long as the required components are present in the first 120 characters (Subtask 3) or 80 characters (Subtask 2).

## **S2.5 Rater training protocol**

Twelve secondary-school teachers of Chinese with five or more years of writing-instruction experience were recruited as raters (six pairs, one pair per grade cluster). Rater training consisted of a fixed six-hour protocol delivered across two consecutive sessions in the week preceding each measurement wave:

- Session 1 (3.0 h): (a) 45-minute conceptual review of schema theory and the primary-trait rubric; (b) 60-minute walk-through of the six anchor scripts (one script per rubric band per subtask, drawn from the pilot corpus); (c) 60-minute paired scoring of 12 held-out pilot scripts followed by structured disagreement resolution; (d) 15-minute review of the calibration decision rules.
- Session 2 (3.0 h): (a) 90-minute independent scoring of a calibration set of 30 pilot scripts; (b) 60-minute cross-pair reconciliation aiming for exact-band agreement > 0.75 and within-one-band agreement > 0.95; (c) 30-minute review of edge cases (mixed genres, blended imagery, non-canonical exemplars).
- A pair proceeded to live scoring only after achieving the calibration thresholds. Ratings on the same response were compared item by item: discrepancies of one band or less were averaged; discrepancies of two bands or more were adjudicated by a senior third rater (university-level Chinese-literature instructor). Across all four waves, adjudication was required for 4.7% of PSCT responses (Supplemental Table S5).

# **Supplemental Appendix S3**

**Imagery Generation and Verification Task (IGVT): item pool, scoring rubric, and rater training protocol**

## **S3.1 Instrument overview**

The Imagery Generation and Verification Task (IGVT) operationalises the imagery-processing mediator. It comprises **16 items** administered in three subtasks: (i) imagery-generation description (6 items), (ii) imagery-to-couplet matching (5 items), and (iii) imagery-detail reporting (5 items). The task is adapted from Kosslyn’s [19] mental-imagery generation-and-inspection paradigm and Sadoski and Paivio’s [33] dual-coding framework, so that a response requires both a verbal and a non-verbal representation of the poem stimulus. Total administration time is approximately 30 minutes per wave.

Psychometric summary across the four measurement waves: Cronbach’s *α* = 0.81 (95% CI: 0.79–0.83); McDonald’s *ω* = 0.82; two-rater ICC(2,1) = 0.84; test–retest reliability (T1–T4, 9-month) r = 0.79; three-factor CFA (subtasks 1–3 as method factors) yielded χ²/df = 2.48, CFI = 0.951, TLI = 0.938, RMSEA = 0.053 (90% CI: 0.043–0.064), SRMR = 0.045.

## **S3.2 Theoretical foundation**

Kosslyn [19] argues that mental imagery has three separable components: image generation from long-term memory, image inspection for detail, and image transformation. The IGVT subtasks map onto these components: Subtask 1 (generation-description) requires generation; Subtask 3 (detail reporting) requires inspection; Subtask 2 (matching) requires transformation-and-verification (the participant must transform a verbal description into a candidate image and verify that image against four couplet alternatives). The Sadoski–Paivio dual-coding framework predicts that classical Chinese poetry, which is highly concrete and imagistic, elicits especially strong non-verbal codes; items were therefore drafted to maximise cross-code activation.

## **S3.3 Full item pool**

***Language note.*** *The original poem titles, stimulus lines, answer options, and Chinese answer terms in this item pool are in Chinese. English translations are provided here for reference only; the original Chinese materials should be used for administration and scoring.*

### **Subtask 1 – Imagery-generation description (Items IGVT-1 to IGVT-6)**

Instruction: “Read each couplet aloud once. Close your eyes for ten seconds and picture the scene. Then, in three to five sentences, describe (a) what you see (objects, colours, positions), (b) what you hear or feel if anything, and (c) the overall mood the image evokes.”

| **Item ID** | **Source poem** | **Stimulus couplet (English translation)** | **Target image elements & mood** |
| --- | --- | --- | --- |
| IGVT-1 | Wang Wei, “On a Mission to the Frontier” | A lone plume of smoke rises straight over the vast desert; the setting sun is round above the long river. | single vertical smoke plume, round setting sun, horizontal river, arid ochre desert; heroic/lonely mood. |
| IGVT-2 | Wang Wei, “An Autumn Evening in the Mountains” | The bright moon shines through the pines; a clear spring flows over the stones. | moonlight filtering through pines, silver-white; clear stream over pale stones; sound of trickling water; serene mood. |
| IGVT-3 | Du Fu, “Quatrain” | Two yellow orioles sing in the green willows; a line of white egrets rises into the blue sky. | two yellow orioles perched on green willows in the foreground, one line of white egrets ascending against blue sky; balanced colour palette (yellow-green-white-blue); joyful mood. |
| IGVT-4 | Du Fu, “Two Quatrains” | Swallows fly over the softening mud; mandarin ducks sleep on the warm sand. | wet dark mud, swallows in flight; warm pale sand-bank, mandarin ducks sleeping; tactile warmth-and-cool contrast; peaceful spring mood. |
| IGVT-5 | Liu Zongyuan, “River Snow” | Across a thousand mountains, no birds fly; along ten thousand paths, no human trace remains. | layered snow-covered mountains extending into the distance; absent birds; blanked paths; whiteness saturating the frame; still, muted mood. |
| IGVT-6 | Su Shi, “Charm of a Maiden: Reminiscences at Red Cliff” | The great river flows east; its waves sweep away the outstanding figures of ages past. | wide dark river surging eastward; large white-crested waves; distant historical figures dissolving into the current; grand, elegiac mood. |

### **Subtask 2 – Imagery-to-couplet matching (Items IGVT-7 to IGVT-11)**

Instruction: “For each prose description below, choose the couplet that best matches the scene. Only one option is correct.”

**IGVT-7.** A silent snowbound scene stretches to the horizon; not a single bird crosses the sky, not a single traveller marks the paths; only an old man in a straw cape sits alone in a small boat and fishes on a river of snow.

- A) Sleeping in spring, I fail to notice dawn; everywhere I hear birds singing
- B) Across a thousand mountains, no birds fly; along ten thousand paths, no human trace remains. In a lone boat, an old man in a straw cape and bamboo hat fishes alone on a cold river in the snow (correct)
- C) The bright sun sinks behind the mountains; the Yellow River flows into the sea
- D) Two yellow orioles sing in the green willows; a line of white egrets rises into the blue sky
- *Answer key: B.*

**IGVT-8.** A quiet moonlit night in a room where a wanderer notices the pale light on the floor by his bed, briefly mistakes it for autumn frost, then looks up at the moon and looks down while thinking of home.

- A) Lifting my head, I gaze at the bright moon; lowering my head, I think of home (correct)
- B) Suddenly, as though a spring breeze had come overnight, thousands upon thousands of pear trees burst into bloom
- C) The children return early from school and hurry to fly their kites in the east wind
- D) Peach Blossom Pool is a thousand feet deep, yet not as deep as Wang Lun’s affection in seeing me off
- *Answer key: A.*

**IGVT-9.** A red sun sinks behind mountains at the western edge of the frame; a great river surges east and drains into the sea; a viewer, wanting to see farther, climbs one more storey of a tower.

- A) The spring river’s tide rises level with the sea; the bright moon rises over the sea with the tide
- B) The bright sun sinks behind the mountains; the Yellow River flows into the sea. To see a thousand miles farther, climb one more storey (correct)
- C) The evening glow and a lone wild duck fly together; the autumn waters and the vast sky share one colour
- D) A lone plume of smoke rises straight over the vast desert; the setting sun is round above the long river
- *Answer key: B.*

**IGVT-10.** A sleeper wakes in spring to birdsong on every side and remembers hearing wind and rain overnight; his first thought is to wonder how many petals have been shaken down.

- A) Sleeping in spring, I fail to notice dawn; everywhere I hear birds singing. Last night came the sound of wind and rain; who knows how many blossoms have fallen? (correct)
- B) I ask where a tavern may be found; a shepherd boy points to Apricot Blossom Village in the distance
- C) I stop my carriage, charmed by the maple woods at dusk; their frost-reddened leaves are redder than the flowers of the second month
- D) From afar I know it is not snow, for a faint fragrance drifts towards me
- *Answer key: A.*

**IGVT-11.** Purple mist rising from a sunlit incense-shaped peak; a distant waterfall hangs like a curtain over the river; the falling water rushes down for what feels like three thousand feet, resembling the Milky Way tipped from the sky.

- A) Sunlight on Incense Burner Peak raises purple mist; from afar, the waterfall hangs above the river. It plunges straight down three thousand feet, as though the Milky Way were falling from the ninth heaven (correct)
- B) No one is seen in the empty mountains, yet human voices echo
- C) Alone in a foreign land, I am a stranger; at every festival, I miss my family all the more
- D) The spring wind greens the southern bank of the Yangtze again; when will the bright moon shine on my return?
- *Answer key: A.*

### **Subtask 3 – Imagery-detail reporting (Items IGVT-12 to IGVT-16)**

Instruction: “For each couplet below, answer the specific detail questions. Give the shortest answer possible.”

**IGVT-12.** Stimulus couplet: *Two yellow orioles sing in the green willows; a line of white egrets rises into the blue sky*

| **Question** | **Correct short answer** |
| --- | --- |
| How many yellow orioles? | 2 |
| On what are they perched? | green willow(s) |
| In what spatial formation do the egrets fly? | a single line / one row |
| In what direction do the egrets go? | upward, toward the sky |
| Name the four colours implied. | yellow, green, white, blue |

**IGVT-13.** Stimulus couplet: *A lone plume of smoke rises straight over the vast desert; the setting sun is round above the long river*

| **Question** | **Correct short answer** |
| --- | --- |
| Landscape type? | desert (vast desert) |
| Number of smoke plumes? | one (a lone plume of smoke) |
| Shape of the smoke? | straight vertical |
| Shape of the sun? | round |
| Position of the sun relative to the river? | setting on / into the long river |

**IGVT-14.** Stimulus couplet: *Bright moonlight lies before the bed; I wonder whether it is frost on the ground*

| **Question** | **Correct short answer** |
| --- | --- |
| Where is the viewer standing/lying? | in front of / by the bed |
| What is the light source? | the bright moon |
| What is the moonlight momentarily mistaken for? | frost |
| Where does the frost illusion appear? | on the ground |

**IGVT-15.** Stimulus couplet: *The young lotus has just shown its pointed tip; already a dragonfly stands upon it*

| **Question** | **Correct short answer** |
| --- | --- |
| Plant depicted? | young lotus / lotus bud |
| State of the lotus? | just emerging, pointed tip |
| What insect appears? | dragonfly |
| Where is the dragonfly? | standing on top of the lotus tip |

**IGVT-16.** Stimulus couplet: *Last night came the sound of wind and rain; who knows how many blossoms have fallen?*

| **Question** | **Correct short answer** |
| --- | --- |
| Time of the remembered event? | night (last night) |
| Weather during the night? | wind and rain |
| What is the poet now uncertain about? | how many petals have fallen |
| Is the closing line a question or a statement? | a rhetorical/reflective question |

## **S3.4 Scoring rubric**

Item scores are aggregated as follows: Subtask 1 (imagery-generation description) is scored on a 0–5 primary-trait rubric per item (6 items × 5 = 30 points). Subtask 2 (matching) is scored dichotomously (5 items × 2 = 10 points). Subtask 3 (imagery-detail reporting) is scored 1 point per correctly answered sub-question, summed within item and capped at 5 per item (5 items × 5 = 25 points). Wave-level totals are standardised (z-scored) before entry into the four-level growth model.

| **Band** | **Descriptor** | **Response evidence at this band** |
| --- | --- | --- |
| 5 | Vivid, complete, mood-accurate | All target image elements are named with appropriate qualifiers (colour, motion, spatial arrangement, sensory register); the mood label matches the target within one adjacent affective category; non-verbal cross-modal detail (sound, temperature, texture) is offered where the couplet permits. |
| 4 | Vivid, mostly complete | At least 80% of target image elements are named with correct qualifiers; mood is identified; one cross-modal detail is present but under-developed, or one non-critical element is missing. |
| 3 | Adequate | At least 60% of target image elements are named; mood label is present but generic (“calm”, “sad”) rather than differentiated; no explicit cross-modal detail. |
| 2 | Sparse | Fewer than half of target image elements are named; mood label is missing or inaccurate; response reads as literal paraphrase. |
| 1 | Emergent | One or two image tokens are named without spatial or relational qualifiers; no coherent scene is described. |
| 0 | Absent or incorrect | Blank; off-topic; or a description of a different poem. |

Conventions: (i) for Subtask 3, spelling variants and Chinese–English mixed answers are accepted so long as the target concept is unambiguous. (ii) A partial detail (e.g., “two birds” instead of “two yellow orioles”) earns 0.5 point rounded up. (iii) Rater pairs re-score any Subtask-1 response whose two independent scores differ by two bands or more; adjudication procedure is identical to that of the PSCT (Section S2.5).

## **S3.5 Rater training protocol**

Rater training used the same six-hour, two-session structure as the PSCT (Section S2.5), with the following adaptations. (a) The anchor-script set contained one imagery-generation script per rubric band and five worked examples of Subtask-3 sub-question scoring. (b) Session-2 calibration required a Cohen’s κ of at least 0.70 on Subtask 1 (weighted κ over adjacent bands) before proceeding to live scoring. (c) The senior third rater held a Master’s degree in Classical Chinese Literature and adjudicated all discrepancies of two bands or more. Across the four waves, adjudication was required for 5.1% of Subtask-1 responses and less than 1% of Subtask-3 responses; matching (Subtask 2) is dichotomous and requires no adjudication.

# **Supplemental Appendix S4**

**Poetry Emotional Resonance Scale (PERS): item pool, scoring rubric, and rater training protocol**

## **S4.1 Instrument overview**

The Poetry Emotional Resonance Scale (PERS) operationalises the emotional-resonance mediator. It contains **12 Likert items and 4 open-response items** organised into four subdimensions: emotion recognition, emotion intensity, empathic engagement, and aesthetic experience. The instrument is a classical-Chinese-poetry-adapted version of Kuiken, Miall, and Sikora’s [21] Literary Response Questionnaire (LRQ); the four subdimensions correspond to LRQ subscales 1, 2, 3, and 5, respectively, with subscale 4 (“concretising imagery”) omitted here because it overlaps conceptually with the IGVT and would inflate common-method variance in the mediation model.

Psychometric summary across the four measurement waves: overall Cronbach’s *α* = 0.86 (95% CI: 0.84–0.88); McDonald’s *ω* = 0.88; subdimension α: emotion recognition 0.79, emotion intensity 0.83, empathic engagement 0.81, aesthetic experience 0.82; two-rater ICC(2,1) = 0.83 for the open-response items; test–retest (T1–T4, 9-month) r = 0.83; four-factor CFA yielded χ²/df = 2.09, CFI = 0.971, TLI = 0.960, RMSEA = 0.046 (90% CI: 0.035–0.058), SRMR = 0.038.

## **S4.2 Administration procedure**

At each of the four measurement waves, the PERS was administered immediately after the poetry-comprehension test and before the writing task. Each wave used one grade-appropriate target poem selected from the corresponding grade’s curriculum syllabus and rotated by wave to control for stimulus-specific effects. The target poem was read aloud once by the classroom administrator and once silently by the student before the PERS was completed. Administration time was 15–20 minutes per wave.

***Language note.*** *The original target poems listed below are in Chinese. Their titles are translated into English for reference only; the original Chinese poems should be used for administration.*

| **Grade** | **Target poem** | **Rationale** |
| --- | --- | --- |
| Grade 3 | Li Bai, “Quiet Night Thoughts” | Concrete imagery, single emotion (homesickness), curriculum-standard poem allowing valid emotion-recognition responses in Wave 1. |
| Grade 4 | Li Bai, “Viewing the Waterfall at Mount Lu” | Hyperbolic imagery affording strong aesthetic-experience responses; single-emotion target (awe / admiration). |
| Grade 5 | Du Fu, “Delight in Rain on a Spring Night” | Layered temporal-emotional structure (anticipation → quiet joy → morning fullness); supports differentiated intensity and empathic responses. |
| Grade 6 | Wang Wei, “An Autumn Evening in the Mountains” | Reflective mood with multi-image aesthetic layering; supports the full four-subdimension response profile. |

## **S4.3 Full item pool**

The 12 Likert items are rated on a 5-point scale (1 = strongly disagree, 2 = disagree, 3 = neither agree nor disagree, 4 = agree, 5 = strongly agree). All items are phrased with reference to the target poem for the current wave and are administered in a fixed within-subdimension order (recognition → intensity → empathy → aesthetic). No items are reverse-scored, following pilot evidence that reverse-scored items in this age range produce inflated random error.

| **Item ID** | **Subdimension** | **Item wording (as read to student)** |
| --- | --- | --- |
| PERS-L1 | Emotion recognition | I can clearly identify the emotions expressed in this poem. |
| PERS-L2 | Emotion recognition | When I read this poem I understand which feelings the poet is trying to convey. |
| PERS-L3 | Emotion recognition | I can name specific feelings in this poem (for example, sadness, joy, longing, admiration). |
| PERS-L4 | Emotion intensity | The emotions in this poem feel strong to me. |
| PERS-L5 | Emotion intensity | This poem stirs deep feelings in me. |
| PERS-L6 | Emotion intensity | While reading, I felt the feelings vividly, not only understood them. |
| PERS-L7 | Empathic engagement | I can picture myself in the situation the poem describes. |
| PERS-L8 | Empathic engagement | I feel a connection to the speaker of the poem. |
| PERS-L9 | Empathic engagement | I share the feelings that the poet is expressing. |
| PERS-L10 | Aesthetic experience | I find the language of this poem beautiful. |
| PERS-L11 | Aesthetic experience | The poem creates a memorable image or mood for me. |
| PERS-L12 | Aesthetic experience | Reading this poem gives me a sense of aesthetic enjoyment. |

The four open-response items ask students to elaborate on each subdimension in their own words. A single open-response item is administered per subdimension (four items total).

| **Item ID** | **Subdimension** | **Open-response prompt** |
| --- | --- | --- |
| PERS-O1 | Emotion recognition | “In the poem you just read, what feelings did you notice? Describe them in one or two sentences, in your own words.” |
| PERS-O2 | Emotion intensity | “Which lines or images in the poem made you feel the most? Why do you think those parts had the strongest effect on you?” |
| PERS-O3 | Empathic engagement | “Have you ever had an experience or feeling similar to the one this poem describes? If yes, briefly describe it. If no, explain why the poet’s feeling nonetheless seems understandable to you.” |
| PERS-O4 | Aesthetic experience | “What aspects of the poem’s language, sound, or images do you find most beautiful, and why?” |

## **S4.4 Scoring**

Likert composite: for each subdimension the mean of the three Likert items is computed (range 1–5); the overall Likert score is the mean of the four subdimension means. Open-response composite: each of the four open items is scored independently by two trained raters on a 0–5 primary-trait rubric (see below); the open-response composite is the sum across four items (range 0–20). The wave-level PERS score entered as a mediator in the four-level growth model is the standardised (z-scored) sum of the mean-centred Likert composite and the mean-centred open-response composite, weighted 0.60 / 0.40 as calibrated during pilot testing to give approximately equal contribution to construct variance.

| **Band** | **Descriptor** | **Response evidence at this band** |
| --- | --- | --- |
| 5 | Insightful, elaborated, poem-anchored | Response names one or more specific feelings; anchors those feelings to one or more concrete words, phrases, or images in the target poem; and offers a personally meaningful justification or comparison. At least three sentences. |
| 4 | Elaborated, poem-anchored | Response names one or more feelings and anchors them to a specific line or image; justification present but shorter or less differentiated. |
| 3 | Adequate | Response names a feeling and offers a general justification without direct anchoring to specific lines or images. |
| 2 | Partial | Response names a feeling without justification, or offers a justification without naming the feeling. |
| 1 | Minimal | Response contains only a generic affective statement (e.g., “it is sad”) with no differentiation and no reference to the poem. |
| 0 | Absent | Blank, off-topic, or explicitly refuses the prompt (“don’t know”). |

## **S4.5 Rater training and administration notes**

Rater training for the four open items followed the same six-hour two-session protocol described in Section S2.5. Because the PERS emphasises subjective response, anchor scripts included examples of the same feeling expressed in age-typical language across bands (a Grade-3 Band-5 response is not required to use the vocabulary of a Grade-6 Band-5 response). To reduce social-desirability bias, students were told at the start of each PERS administration that “there are no right or wrong feelings” and that the response would not be scored on how the student felt, only on how completely and clearly the response described that feeling. The Likert portion was self-administered with adult read-aloud support available on request; the open-response portion was written.

# **Supplemental Table S5**

**Reliability and validity indices for the three mediator instruments across the four measurement waves**

This table consolidates the psychometric evidence for the PSCT, IGVT, and PERS across the four longitudinal measurement waves (T1: October 2024, T2: January 2025, T3: April 2025, T4: July 2025). Values in the “Overall” column pool the four waves and are the values cited in Section 3.3 and Table 4 of the main manuscript.

| **Instrument** | **Index** | **T1** | **T2** | **T3** | **T4** | **Overall (95% CI)** |
| --- | --- | --- | --- | --- | --- | --- |
| PSCT | Cronbach's α | 0.82 | 0.83 | 0.85 | 0.86 | 0.84 (0.82–0.86) |
| PSCT | McDonald's ω | 0.83 | 0.84 | 0.86 | 0.87 | 0.85 (0.83–0.87) |
| PSCT | ICC(2,1) | 0.85 | 0.86 | 0.88 | 0.89 | 0.87 (0.85–0.89) |
| PSCT | CFA CFI | 0.961 | 0.968 | 0.963 | 0.969 | 0.965 |
| PSCT | CFA RMSEA | 0.051 | 0.047 | 0.050 | 0.048 | 0.049 |
| PSCT | Rater adjudication % | 5.1 | 4.8 | 4.5 | 4.4 | 4.7 |
| IGVT | Cronbach's α | 0.79 | 0.80 | 0.82 | 0.83 | 0.81 (0.79–0.83) |
| IGVT | McDonald's ω | 0.80 | 0.81 | 0.83 | 0.84 | 0.82 (0.80–0.84) |
| IGVT | ICC(2,1) | 0.82 | 0.83 | 0.85 | 0.86 | 0.84 (0.82–0.86) |
| IGVT | CFA CFI | 0.966 | 0.955 | 0.949 | 0.953 | 0.951 |
| IGVT | CFA RMSEA | 0.055 | 0.051 | 0.054 | 0.052 | 0.053 |
| IGVT | Rater adjudication % | 5.5 | 5.2 | 4.9 | 4.8 | 5.1 |
| PERS | Cronbach's α | 0.84 | 0.85 | 0.87 | 0.88 | 0.86 (0.84–0.88) |
| PERS | McDonald's ω | 0.86 | 0.87 | 0.89 | 0.90 | 0.88 (0.86–0.90) |
| PERS | ICC(2,1) (open items) | 0.81 | 0.82 | 0.84 | 0.85 | 0.83 (0.81–0.85) |
| PERS | CFA CFI | 0.968 | 0.974 | 0.969 | 0.973 | 0.971 |
| PERS | CFA RMSEA | 0.048 | 0.044 | 0.047 | 0.045 | 0.046 |
| PERS | Rater adjudication % | 5.3 | 5.0 | 4.7 | 4.6 | 4.9 |

***Note.*** All within-wave *N* = 1,920 students (48 classes across 12 schools). The mild upward drift in reliability across waves reflects the combined effect of familiarisation with the instruments and the deepening differentiation of poetic competence between students, both of which reduce measurement error variance relative to construct variance. Test–retest reliability (T1–T4, 9-month interval): PSCT r = 0.81 (95% CI: 0.78–0.84); IGVT r = 0.79 (0.76–0.82); PERS r = 0.83 (0.80–0.86). Convergent-validity correlations with theoretically related external measures (Section 3.3 of the main text): PSCT with a Chinese adaptation of the Reading Comprehension Schema Survey, r = 0.68; IGVT with the Vividness of Visual Imagery Questionnaire (child form), r = 0.62; PERS with a Chinese short form of the LRQ, r = 0.71. Divergent-validity correlations with unrelated measures (arithmetic-fluency subtest of the Wechsler Intelligence Scale for Children): all |r| ≤ 0.19. CFA CFI and RMSEA refer to the measurement models described in Sections S2.1, S3.1, and S4.1, and the Overall column gives the pooled estimates.

# **Supplemental Table S6**

Measurement analyses of the adapted NASA-TLX load-type mapping

This table documents the four analyses of the interpretive mapping from the adapted NASA-TLX subscales to intrinsic (ICL), extraneous (ECL), and germane (GCL) cognitive load that are summarized in Sections 3.3 and 4.6.4 of the main manuscript: competing measurement models, measurement invariance across grades, a multitrait-multimethod comparison with the Klepsch et al. instrument [18], a re-estimation with Klepsch-based scores, and a sensitivity model with an undifferentiated load score. Factor models were estimated in lavaan [32].

Panel A. Competing measurement models of the TLX subscales (pooled across T1 to T4)

| **Model** | $\chi^{2}$**/df** | **CFI** | **TLI** | **RMSEA** | **SRMR** | **Best fit at waves** |
| --- | --- | --- | --- | --- | --- | --- |
| One-factor (general load) | 4.51 | 0.893 | 0.861 | 0.093 | 0.074 | 0 of 4 |
| Two-factor (task-oriented versus effort-oriented) | 3.21 | 0.932 | 0.906 | 0.069 | 0.057 | 0 of 4 |
| Three-factor (ICL, ECL, GCL) | 2.35 | 0.960 | 0.950 | 0.048 | 0.042 | 4 of 4 |

Panel B. Measurement invariance of the three-factor model across Grades 3 to 6

| **Step** | $\chi^{2}$**/df** | **CFI** | **RMSEA** | $\Delta$**CFI** | $\Delta$**RMSEA** | **Decision** |
| --- | --- | --- | --- | --- | --- | --- |
| Configural | 2.28 | 0.961 | 0.047 | - | - | Retained |
| Metric (equal loadings) | 2.31 | 0.957 | 0.048 | 0.004 | 0.001 | Retained |
| Scalar (equal loadings and intercepts) | 2.39 | 0.950 | 0.050 | 0.007 | 0.002 | Retained |

Panel C. Multitrait-multimethod summary, NASA-TLX and Klepsch et al. instrument ($n$ = 384, T3)

| **Correlation type** | **ICL** | **ECL** | **GCL** | **Mean** |
| --- | --- | --- | --- | --- |
| Monotrait-heteromethod (convergent, disattenuated) | 0.69 | 0.73 | 0.71 | 0.71 |
| Heterotrait-heteromethod | 0.24 | 0.31 | 0.29 | 0.28 |
| Heterotrait-monomethod, NASA-TLX | 0.36 | 0.42 | 0.39 | 0.39 |
| Heterotrait-monomethod, Klepsch et al. | 0.33 | 0.38 | 0.35 | 0.35 |

Panel D. Re-estimation of the O1 paths and the mediation index with Klepsch-based load scores ($n$ = 384)

| **Estimate** | **NASA-TLX (main analysis)** | **Klepsch et al.** | **Absolute difference** |
| --- | --- | --- | --- |
| ICL $\to$ Writing ability | -0.41 | -0.37 | 0.04 |
| ECL $\to$ Writing ability | -0.49 | -0.47 | 0.02 |
| GCL $\to$ Writing ability | 0.45 | 0.48 | 0.03 |
| Mediation index | 0.587 | 0.56 [0.47, 0.65] | 0.03 |

Panel E. Sensitivity model with an undifferentiated load score

| **Model** | **AIC difference** | **Teaching** $\times$ **load interaction (**$\gamma$**)** | **p** |
| --- | --- | --- | --- |
| Three load components (main model) | 0 (reference) | 0.47 | .011 |
| Single undifferentiated load score | +68.4 | 0.09 | .53 |

*Note. ICL, ECL, and GCL denote the adapted NASA-TLX indices of intrinsic, extraneous, and germane cognitive load. In Panel B,* $\Delta$*CFI is the decrease and* $\Delta$*RMSEA the increase relative to the preceding step, and a CFI decrease below 0.01 was the invariance criterion. In Panel C, heterotrait entries are averages over the correlations that involve the trait in the column, and every monotrait-heteromethod correlation exceeds the corresponding heterotrait-monomethod correlations. Panel D uses the stratified 20% subsample described in Section 4.6.4.*

# **Supplemental Table S7**

Fold-wise analysis pipeline, leakage checklist, and specification selection across folds

This table documents the sequence of data-dependent steps described in Section 3.6, Algorithm 1 (step 7), and Figure 6B of the main manuscript. Every step is estimated on the training folds of the current design and applied unchanged to the validation fold, and validation outcomes enter only the performance metrics.

Panel A. Data-dependent steps and their handling in each fold

| **Step** | **Operation** | **Estimated on** | **Applied to the validation fold** | **Validation outcomes used** |
| --- | --- | --- | --- | --- |
| 1 | Multiple imputation of missing predictors (predictive mean matching, $m$ = 20) | Training folds | Missing validation predictors imputed from the training-fold models | No |
| 2 | Centering of Level-1 predictors | Each student's own predictor values | Same rule | No |
| 3 | Centering of Level-2 to Level-4 predictors | Training-fold grand means | Training-fold grand means | No |
| 4 | Selection of growth order and random-effects structure (AIC/BIC) | Training folds | Selected specification | No |
| 5 | Estimation of the four-level model (REML) | Training folds | Fixed effects | No |
| 6 | Class-level and school-level BLUPs | Training folds | Design A: both; Design B: school BLUP only; Design C: none | No |
| 7 | Grade-normed 30th and 70th percentile band cutoffs | Observed training-fold scores | Observed and predicted validation scores | No |
| 8 | Band probabilities for ROC-AUC | Empirical distribution of training-fold residuals | Validation predictions | No |
| 9 | Leave-one-covariate-out importance | Reduced models refitted on the training folds | Validation-fold MSE | Scoring only |
| 10 | Averaging over the 20 imputed datasets | Fold-wise predictions | Validation predictions | No |
| 11 | Performance metrics ($R^{2}$, RMSE, MAE, calibration, accuracy, macro AUC) | Out-of-fold predictions | Validation outcomes | Scoring only |
| 12 | Bootstrap interval of $R^{2}$ for Design A | 1,000 student-level resamples | Out-of-fold predictions | Scoring only |

Panel B. Specification selected across folds

| **Specification** | **Design A (10 folds)** | **Design B (48 folds)** | **Design C (12 folds)** |
| --- | --- | --- | --- |
| Quadratic growth order | 10 of 10 | 48 of 48 | 12 of 12 |
| Cubic growth order | 0 of 10 | 0 of 48 | 0 of 12 |
| Random intercepts and linear time slopes at Levels 2 to 4 | 10 of 10 | 48 of 48 | 12 of 12 |

*Note. Student random effects are set to zero in all three designs (Section 3.6). Validation observations without an observed outcome are excluded from the performance metrics and are not imputed. The frequencies in Panel B refer to the specification with the lowest AIC and BIC in each training fold.*

# **Supplemental Table S8**

Fold-level, class-level, and school-level predictive performance under the three validation designs

This table reports the disaggregated performance behind Table 8 of the main manuscript: the ten student-grouped folds of Design A, the calibration and band-specific ROC-AUC of the three designs, the 48 held-out classes of Design B, and the 12 held-out schools of Design C. Pooled values are those reported in Table 8.

Panel A. Design A, student-grouped 10-fold cross-validation (fold level)

| **Fold** | **Students** | $R^{2}$ | **RMSE** | **MAE** | **Calibration slope** | **Calibration intercept** | **Accuracy (%)** | **Macro AUC** |
| --- | --- | --- | --- | --- | --- | --- | --- | --- |
| 1 | 192 | 0.852 | 4.21 | 2.97 | 1.03 | -2.08 | 80.7 | 0.921 |
| 2 | 192 | 0.841 | 4.37 | 3.10 | 0.98 | 1.52 | 79.8 | 0.915 |
| 3 | 192 | 0.857 | 4.12 | 2.93 | 1.04 | -2.97 | 82.1 | 0.924 |
| 4 | 192 | 0.834 | 4.43 | 3.12 | 0.97 | 2.25 | 78.6 | 0.911 |
| 5 | 192 | 0.848 | 4.26 | 3.00 | 1.01 | -0.81 | 80.2 | 0.917 |
| 6 | 192 | 0.862 | 4.08 | 2.89 | 1.05 | -3.49 | 82.5 | 0.926 |
| 7 | 192 | 0.839 | 4.39 | 3.08 | 0.99 | 0.63 | 79.5 | 0.916 |
| 8 | 192 | 0.850 | 4.24 | 3.01 | 1.02 | -1.51 | 81.3 | 0.921 |
| 9 | 192 | 0.830 | 4.49 | 3.15 | 0.96 | 2.96 | 77.8 | 0.908 |
| 10 | 192 | 0.853 | 4.23 | 2.95 | 1.05 | -3.70 | 80.4 | 0.921 |
| Mean (SD) | 192 | 0.847 (0.010) | 4.28 (0.13) | 3.02 (0.09) | 1.01 (0.03) | -0.72 (2.43) | 80.3 (1.5) | 0.918 (0.006) |
| Pooled (Table 8) | 1,920 | 0.847 | 4.28 | 3.02 | 1.01 | -0.68 | 80.3 | 0.918 |

Panel B. Calibration and band-specific ROC-AUC of the pooled out-of-fold predictions

| **Design** | **Calibration slope** | **Calibration intercept** | **AUC, Low band** | **AUC, Medium band** | **AUC, High band** | **Macro AUC** |
| --- | --- | --- | --- | --- | --- | --- |
| A. Student-grouped 10-fold CV | 1.01 | -0.68 | 0.953 | 0.838 | 0.963 | 0.918 |
| B. Leave-one-class-out CV | 0.96 | 2.89 | 0.919 | 0.771 | 0.932 | 0.874 |
| C. Leave-one-school-out CV | 0.94 | 4.43 | 0.865 | 0.702 | 0.869 | 0.812 |

Panel C. Design B, leave-one-class-out cross-validation (48 held-out classes)

| **Class** | **Location** | **Grade** | **Students** | **RMSE** | **MAE** | **Mean bias** | **Accuracy (%)** |
| --- | --- | --- | --- | --- | --- | --- | --- |
| S01-G3 | Urban | Grade 3 | 40 | 5.95 | 4.79 | 3.7 | 70.4 |
| S01-G4 | Urban | Grade 4 | 40 | 5.97 | 4.21 | -3.2 | 70.4 |
| S01-G5 | Urban | Grade 5 | 40 | 4.34 | 3.03 | -1.3 | 72.3 |
| S01-G6 | Urban | Grade 6 | 40 | 4.68 | 3.18 | -1.9 | 71.6 |
| S02-G3 | Urban | Grade 3 | 40 | 6.74 | 5.49 | -4.2 | 77.2 |
| S02-G4 | Urban | Grade 4 | 40 | 4.62 | 3.03 | 1.4 | 76.2 |
| S02-G5 | Urban | Grade 5 | 40 | 4.99 | 3.12 | -1.5 | 74.4 |
| S02-G6 | Urban | Grade 6 | 40 | 4.58 | 3.01 | 1.8 | 74.7 |
| S03-G3 | Urban | Grade 3 | 40 | 3.79 | 2.75 | 0.6 | 75.3 |
| S03-G4 | Urban | Grade 4 | 40 | 5.03 | 3.42 | -1.5 | 76.4 |
| S03-G5 | Urban | Grade 5 | 40 | 6.33 | 5.10 | 3.9 | 68.4 |
| S03-G6 | Urban | Grade 6 | 40 | 4.39 | 2.96 | -0.6 | 74.4 |
| S04-G3 | Urban | Grade 3 | 40 | 4.50 | 3.30 | 2.2 | 77.2 |
| S04-G4 | Urban | Grade 4 | 40 | 5.60 | 4.02 | -3.0 | 75.6 |
| S04-G5 | Urban | Grade 5 | 40 | 4.55 | 3.07 | 1.0 | 76.4 |
| S04-G6 | Urban | Grade 6 | 40 | 4.55 | 2.98 | 0.6 | 73.8 |
| S05-G3 | Suburban | Grade 3 | 40 | 4.50 | 3.04 | -0.7 | 76.0 |
| S05-G4 | Suburban | Grade 4 | 40 | 4.68 | 3.17 | 2.2 | 75.4 |
| S05-G5 | Suburban | Grade 5 | 40 | 6.19 | 4.70 | -3.6 | 69.0 |
| S05-G6 | Suburban | Grade 6 | 40 | 5.80 | 4.12 | 3.1 | 70.5 |
| S06-G3 | Suburban | Grade 3 | 40 | 7.44 | 6.57 | -5.1 | 66.2 |
| S06-G4 | Suburban | Grade 4 | 40 | 4.31 | 3.43 | -1.4 | 75.0 |
| S06-G5 | Suburban | Grade 5 | 40 | 5.83 | 4.03 | 3.0 | 74.4 |
| S06-G6 | Suburban | Grade 6 | 40 | 8.11 | 7.06 | -5.5 | 63.9 |
| S07-G3 | Suburban | Grade 3 | 40 | 4.68 | 3.17 | -1.8 | 75.6 |
| S07-G4 | Suburban | Grade 4 | 40 | 5.60 | 3.87 | 2.9 | 73.2 |
| S07-G5 | Suburban | Grade 5 | 40 | 5.17 | 3.25 | -2.1 | 71.0 |
| S07-G6 | Suburban | Grade 6 | 40 | 5.01 | 3.41 | -1.3 | 75.0 |
| S08-G3 | Rural | Grade 3 | 40 | 4.31 | 3.17 | 0.2 | 76.4 |
| S08-G4 | Rural | Grade 4 | 40 | 4.50 | 3.31 | -0.6 | 72.6 |
| S08-G5 | Rural | Grade 5 | 40 | 5.02 | 3.43 | 2.0 | 75.1 |
| S08-G6 | Rural | Grade 6 | 40 | 4.68 | 3.17 | -0.7 | 76.1 |
| S09-G3 | Rural | Grade 3 | 40 | 4.55 | 3.33 | -0.3 | 75.0 |
| S09-G4 | Rural | Grade 4 | 40 | 5.23 | 3.82 | 2.9 | 69.5 |
| S09-G5 | Rural | Grade 5 | 40 | 4.76 | 3.22 | 0.5 | 75.6 |
| S09-G6 | Rural | Grade 6 | 40 | 4.95 | 3.09 | 0.6 | 76.1 |
| S10-G3 | Rural | Grade 3 | 40 | 4.44 | 3.09 | -0.4 | 77.2 |
| S10-G4 | Rural | Grade 4 | 40 | 4.69 | 3.17 | -0.0 | 77.1 |
| S10-G5 | Rural | Grade 5 | 40 | 5.62 | 3.87 | -2.6 | 73.2 |
| S10-G6 | Rural | Grade 6 | 40 | 3.97 | 2.68 | -0.6 | 74.4 |
| S11-G3 | Rural | Grade 3 | 40 | 4.58 | 3.01 | -1.3 | 75.0 |
| S11-G4 | Rural | Grade 4 | 40 | 4.78 | 3.16 | -1.7 | 74.8 |
| S11-G5 | Rural | Grade 5 | 40 | 4.77 | 2.97 | 0.7 | 74.7 |
| S11-G6 | Rural | Grade 6 | 40 | 6.48 | 5.19 | 4.0 | 73.2 |
| S12-G3 | Rural | Grade 3 | 40 | 3.32 | 2.63 | 1.0 | 76.3 |
| S12-G4 | Rural | Grade 4 | 40 | 6.22 | 4.41 | 3.4 | 68.2 |
| S12-G5 | Rural | Grade 5 | 40 | 4.48 | 3.12 | 0.2 | 77.2 |
| S12-G6 | Rural | Grade 6 | 40 | 7.76 | 6.96 | 5.4 | 74.8 |
| Mean (SD) | - | - | 40 | 5.15 (1.00) | 3.71 (1.06) | 0.0 (2.5) | 73.8 (3.1) |
| Pooled (Table 8) | - | - | 1,920 | 5.24 | 3.71 | - | 73.8 |

Panel D. Design C, leave-one-school-out cross-validation (12 held-out schools)

| **School** | **Location** | **Students** | **RMSE** | **MAE** | **Mean bias** | **Accuracy (%)** | **Macro AUC** |
| --- | --- | --- | --- | --- | --- | --- | --- |
| S01 | Urban | 160 | 6.97 | 4.80 | 4.7 | 63.7 | 0.814 |
| S02 | Urban | 160 | 6.28 | 4.29 | 3.3 | 64.8 | 0.816 |
| S03 | Urban | 160 | 7.23 | 5.41 | -5.2 | 62.9 | 0.783 |
| S04 | Urban | 160 | 5.45 | 4.01 | -1.6 | 75.4 | 0.819 |
| S05 | Suburban | 160 | 5.73 | 3.63 | -2.3 | 66.2 | 0.822 |
| S06 | Suburban | 160 | 6.37 | 4.72 | 3.9 | 67.8 | 0.817 |
| S07 | Suburban | 160 | 5.44 | 4.00 | -0.5 | 77.5 | 0.813 |
| S08 | Rural | 160 | 5.99 | 4.39 | 2.1 | 69.2 | 0.818 |
| S09 | Rural | 160 | 6.66 | 4.94 | -4.3 | 62.7 | 0.803 |
| S10 | Rural | 160 | 7.45 | 5.60 | 5.6 | 62.3 | 0.800 |
| S11 | Rural | 160 | 6.44 | 4.30 | -3.6 | 64.1 | 0.829 |
| S12 | Rural | 160 | 6.19 | 4.56 | -3.1 | 66.2 | 0.810 |
| Mean (SD) | - | 160 | 6.35 (0.65) | 4.55 (0.58) | -0.1 (3.8) | 66.9 (4.9) | 0.812 (0.012) |
| Pooled (Table 8) | - | 1,920 | 6.38 | 4.55 | - | 66.9 | 0.812 |

*Note. Mean bias is the mean of predicted minus observed writing scores in the held-out unit, and the calibration intercept is expressed on the 0 to 100 writing scale. Class-level and school-level RMSE values are computed within the held-out unit; the pooled RMSE in Table 8 is the square root of the mean squared error over all held-out observations, which the class-level and school-level values reproduce as their root mean square.* $R^{2}$ *and calibration are reported for the pooled predictions of Designs B and C because a single class or school spans a restricted range of writing scores. Classes are identified by school and grade; school identifiers and locations follow Supplemental Table S1.*

# **Supplemental Table S9**

Analytic registry of confirmatory, exploratory, and sensitivity analyses

The registry lists every inferential test reported in the main manuscript with its tier, multiplicity family, pre-specification status, and raw and adjusted p values. The confirmatory tests form one Holm family ($k$ = 8), the exploratory tests in Table 9 form one Benjamini-Hochberg family ($k$ = 6), and the exploratory tests documented in Section 4.5.3 form a second Benjamini-Hochberg family ($k$ = 4). Sensitivity analyses carry no separate inferential claims.

| **ID** | **Objective** | **Test** | **Tier** | **Family** | **Pre-specified** | **Estimate** | **Raw** $p$ | **Adjusted** $p$ | **Reported in** |
| --- | --- | --- | --- | --- | --- | --- | --- | --- | --- |
| C1 | O1 | ICL $\to$ Writing ability | Confirmatory | Holm ($k$ = 8) | Yes | -0.41 | <.001 | <.001 | Table 9 |
| C2 | O1 | ECL $\to$ Writing ability | Confirmatory | Holm ($k$ = 8) | Yes | -0.49 | <.001 | <.001 | Table 9 |
| C3 | O1 | GCL $\to$ Writing ability | Confirmatory | Holm ($k$ = 8) | Yes | 0.45 | <.001 | <.001 | Table 9 |
| C4 | O3 | Indirect via schema construction | Confirmatory | Holm ($k$ = 8) | Yes | 0.242 | <.001 | <.001 | Table 9; Figure 10B |
| C5 | O3 | Indirect via imagery processing | Confirmatory | Holm ($k$ = 8) | Yes | 0.189 | <.001 | <.001 | Table 9; Figure 10B |
| C6 | O3 | Indirect via emotional resonance | Confirmatory | Holm ($k$ = 8) | Yes | 0.156 | <.001 | <.001 | Table 9; Figure 10B |
| C7 | O5 | Teaching $\times$ CogLoad | Confirmatory | Holm ($k$ = 8) | Yes | 0.47 | .011 | .011 | Table 9; Figure 10A; Table 11 |
| C8 | O6 | Quadratic growth (fixed) | Confirmatory | Holm ($k$ = 8) | Yes | -0.17 | <.001 | <.001 | Table 9; Figure 9 |
| E1 | O2 | Poetry – Working memory | Exploratory | BH 1 ($k$ = 6) | Yes | 0.38 | <.001 | <.001 | Table 9 |
| E2 | O2 | Poetry – Prior knowledge | Exploratory | BH 1 ($k$ = 6) | Yes | 0.41 | <.001 | <.001 | Table 9 |
| E3 | O4 | Working memory $\to$ Writing | Exploratory | BH 1 ($k$ = 6) | Yes | 0.31 | <.001 | <.001 | Table 9 |
| E4 | O4 | Prior knowledge $\to$ Writing | Exploratory | BH 1 ($k$ = 6) | Yes | 0.35 | <.001 | <.001 | Table 9 |
| E5 | O5 | Grade $\times$ CogLoad | Exploratory | BH 1 ($k$ = 6) | Yes | 0.39 | .019 | .023 | Table 9; Table 11 |
| E6 | O5 | Environment $\times$ CogLoad | Exploratory | BH 1 ($k$ = 6) | Yes | 0.31 | .038 | .038 | Table 9; Table 11 |
| E7 | - | ICL $\to$ Vocabulary richness | Exploratory | BH 2 ($k$ = 4) | No | -0.09 | .028 | .11 | Section 4.5.3 |
| E8 | - | School type $\times$ Grade on intercepts | Exploratory | BH 2 ($k$ = 4) | No | 0.14 | .173 | .23 | Section 4.5.3 |
| E9 | - | Emotional resonance $\times$ ECL | Exploratory | BH 2 ($k$ = 4) | No | 0.10 | .090 | .18 | Section 4.5.3 |
| E10 | - | Urban-rural difference in the mediation index | Exploratory | BH 2 ($k$ = 4) | No | 0.03 | .290 | .29 | Section 4.5.3 |
| R1 | - | RI-CLPM within-person lagged paths | Sensitivity | - | No | - | - | - | Section 4.3.5; Table 7 |
| R2 | - | Robustness across estimation methods | Sensitivity | - | No | - | - | - | Section 4.6.1; Table 10 |
| R3 | - | Cognitive-load instrumentation and mapping | Sensitivity | - | No | - | - | - | Section 4.6.4; Table S6 |
| R4 | - | Small-cluster analyses at Level 4 | Sensitivity | - | No | - | - | - | Section 4.6.5; Table 11; Tables S10 to S12 |
| R5 | - | Covariate adjustment and confounding thresholds | Sensitivity | - | No | - | - | - | Section 4.6.6; Table 12; Table S13 |

Panel B. Stability criteria for the Level-4 and cross-level estimates, specified before the small-cluster analyses were run

| **Criterion** | **Analysis** | **Requirement for Stable** |
| --- | --- | --- |
| 1 | Leave-one-school-out refits (12) | Sign unchanged in all 12 refits |
| 2 | Kenward-Roger (coefficients) or profile-likelihood (variance components) 95% interval | Interval excludes zero |
| 3 | School-level wild cluster bootstrap, Webb weights, $B$ = 9,999 (school-level case bootstrap for the variance component and $ICC_{school}$) | 95% interval excludes zero |
| 4 | Bayesian re-estimation under four priors | 95% credible interval excludes zero under every prior |
| 5 | Monte Carlo simulation of 1,000 datasets with the observed 12-school design | Coverage of the Kenward-Roger or profile interval of at least 0.925 |

Panel C. Track of every Level-4 and cross-level estimate

| **Estimate** | **Level** | **Track** | **Family** | **Status in Table 11** | **Use in the discussion** |
| --- | --- | --- | --- | --- | --- |
| $\tau_{v_{000}}$ | Level 4 | Estimation (interval only) | - | Imprecise | Not interpreted in magnitude |
| $ICC_{school}$ | Level 4 | Estimation (interval only) | - | Imprecise | Not interpreted in magnitude |
| Teaching $\times$ CogLoad | Cross-level | Confirmatory (C7) | Holm ($k$ = 8) | Stable | Substantive |
| Grade $\times$ CogLoad | Cross-level | Exploratory (E5) | BH 1 ($k$ = 6) | Exploratory | Hypothesis-generating |
| Environment $\times$ CogLoad | Cross-level | Exploratory (E6) | BH 1 ($k$ = 6) | Exploratory | Hypothesis-generating |
| Moderated-mediation index | Cross-level | Estimation (interval only) | - | Stable | Substantive |

*Note. Adjusted p values were computed from unrounded raw p values; for the quadratic growth term (C8) the raw p value of .0001 gives a Holm-adjusted value of .0002. The stability classification of the Level-4 and cross-level estimates in Table 11 is recorded under R4 and does not alter the tier of C7, E5, or E6; a variance component or ICC whose profile-likelihood and bootstrap intervals reach the boundary of the parameter space is classified as Imprecise, and its magnitude is not interpreted.*

# **Supplemental Table S10**

Leave-one-school-out estimates of Level-4 and cross-level parameters

Each row gives the estimates obtained after omitting one school and refitting the four-level model on the remaining 11 schools (Section 4.6.5 of the main manuscript). The LOSO ranges and the most influential schools summarized in Table 11 are taken from this table. Models were fitted with lme4 and lmerTest [3, 22].

| **Omitted school** | **Location** | $\tau_{v_{000}}$ | $ICC_{school}$ | **Teaching** $\times$ **CogLoad** | **Grade** $\times$ **CogLoad** | **Environment** $\times$ **CogLoad** | **Moderated-mediation index** |
| --- | --- | --- | --- | --- | --- | --- | --- |
| None (main model) | - | 10.03 | 0.083 | 0.47 | 0.39 | 0.31 | 0.070 |
| S01 | Urban | 7.71 | 0.065 | 0.48 | 0.41 | 0.33 | 0.071 |
| S02 | Urban | 9.64 | 0.080 | 0.45 | 0.52 | 0.30 | 0.068 |
| S03 | Urban | 6.99 | 0.059 | 0.56 | 0.37 | 0.35 | 0.076 |
| S04 | Urban | 10.97 | 0.090 | 0.49 | 0.40 | 0.31 | 0.098 |
| S05 | Suburban | 10.38 | 0.086 | 0.44 | 0.35 | 0.15 | 0.063 |
| S06 | Suburban | 9.11 | 0.076 | 0.46 | 0.42 | 0.34 | 0.072 |
| S07 | Suburban | 14.88 | 0.118 | 0.48 | 0.38 | 0.28 | 0.069 |
| S08 | Rural | 10.44 | 0.086 | 0.39 | 0.44 | 0.37 | 0.074 |
| S09 | Rural | 8.47 | 0.071 | 0.50 | 0.23 | 0.29 | 0.067 |
| S10 | Rural | 6.21 | 0.053 | 0.47 | 0.40 | 0.32 | 0.073 |
| S11 | Rural | 9.43 | 0.078 | 0.43 | 0.33 | 0.26 | 0.038 |
| S12 | Rural | 9.73 | 0.081 | 0.49 | 0.43 | 0.43 | 0.081 |

*Note.* $\tau_{v_{000}}$ *= school-level intercept variance;* $ICC_{school}$ *= school-level intraclass correlation. Cross-level interactions are unstandardized coefficients (*$\gamma$*). Each refit kept the specification of the main model and was estimated by REML. The sign of every estimate is unchanged across the 12 refits, and the school whose omission changes an estimate most is reported as the most influential school in Table 11. School identifiers and locations follow Supplemental Table S1.*

# **Supplemental Table S11**

Prior sensitivity of Level-4 and cross-level parameters

Posterior medians and 95% credible intervals from the Bayesian re-estimation of the four-level model under the reference prior and three alternative priors on the random-effect standard deviations (Section 4.6.5 of the main manuscript). Models were estimated with brms [5] using four chains of 10,000 iterations, of which 5,000 were warm-up iterations.

| **Parameter** | **Inv-Gamma(0.001, 0.001) on variances (reference)** | **Half-Cauchy(0, 5)** | **Half-Student-t(3, 0, 10)** | **Uniform(0, 30)** | **Intervals excluding 0** |
| --- | --- | --- | --- | --- | --- |
| $\tau_{v_{000}}$ | 7.21 [0.38, 33.26] | 9.64 [1.12, 35.48] | 10.42 [1.46, 38.07] | 12.18 [2.03, 44.95] | 4 of 4 |
| $ICC_{school}$ | 0.061 [0.003, 0.231] | 0.080 [0.010, 0.244] | 0.086 [0.013, 0.258] | 0.099 [0.018, 0.290] | 4 of 4 |
| Teaching $\times$ CogLoad | 0.46 [0.12, 0.80] | 0.47 [0.11, 0.82] | 0.47 [0.10, 0.84] | 0.48 [0.13, 0.83] | 4 of 4 |
| Grade $\times$ CogLoad | 0.38 [0.06, 0.70] | 0.39 [0.04, 0.74] | 0.39 [0.02, 0.77] | 0.40 [-0.01, 0.80] | 3 of 4 |
| Environment $\times$ CogLoad | 0.31 [0.05, 0.57] | 0.32 [0.02, 0.61] | 0.32 [-0.01, 0.64] | 0.33 [-0.03, 0.66] | 2 of 4 |
| Moderated-mediation index | 0.07 [0.03, 0.11] | 0.07 [0.03, 0.11] | 0.07 [0.03, 0.11] | 0.07 [0.04, 0.11] | 4 of 4 |

*Note. The alternative priors are placed on the random-effect standard deviations. For* $\tau_{v_{000}}$ *and* $ICC_{school}$*, Table 11 of the main manuscript reports the smallest and largest posterior medians across the four priors; for the coefficients it reports the lowest lower bound and the highest upper bound of the 95% credible intervals. All models reached* $\hat{R}$ *< 1.01 with bulk and tail effective sample sizes of at least 400.*

# **Supplemental Table S12**

Calibrated Monte Carlo simulation of small-cluster inference

One thousand datasets were generated from the fitted four-level model with the observed design of 12 schools, 48 classes, 1,920 students, and four waves, and each dataset was analyzed with the inference procedures described in Sections 3.5 and 4.6.5 of the main manuscript.

| **Parameter** | **True value** | **Mean estimate** | **Relative bias (%)** | **Empirical SD** | **Coverage, KR or profile 95% CI** | **Coverage, wild or school-level bootstrap 95% interval** | **Power** |
| --- | --- | --- | --- | --- | --- | --- | --- |
| $\tau_{v_{000}}$ | 10.03 | 10.12 | +0.9 | 7.49 | 0.959 | 0.818 | 0.57 |
| $ICC_{school}$ | 0.083 | 0.082 | -1.2 | 0.057 | 0.951 | 0.806 | 0.57 |
| Teaching $\times$ CogLoad | 0.47 | 0.468 | -0.4 | 0.181 | 0.945 | 0.936 | 0.72 |
| Grade $\times$ CogLoad | 0.39 | 0.387 | -0.8 | 0.158 | 0.912 | 0.903 | 0.68 |
| Environment $\times$ CogLoad | 0.31 | 0.306 | -1.3 | 0.146 | 0.898 | 0.919 | 0.55 |
| Moderated-mediation index | 0.07 | 0.068 | -2.9 | 0.021 | 0.930 | 0.925 | 0.85 |

*Note. Relative bias = (mean estimate - true value) / true value* $\times$ *100. Coverage is the proportion of the 1,000 intervals that contain the true value; the Kenward-Roger (KR) interval applies to the coefficients and the profile-likelihood interval to the variance component and the ICC. The KR coverage values are those reported in Table 11 of the main manuscript, where a coverage of at least 0.925 was one of the stability criteria. The bootstrap coverage refers to the school-level wild cluster bootstrap for the coefficients and to the school-level case bootstrap for the variance component and the ICC. Power is the proportion of datasets in which the KR 95% interval excluded zero; for the variance component and the ICC it is the proportion of datasets in which the boundary-corrected likelihood-ratio test rejected at* $\alpha$ *= .05.*

# **Supplemental Table S13**

Covariate-adjusted RI-CLPM paths and residual-correlation thresholds for the temporally ordered indirect associations

Panel A reports the within-person cross-lagged coefficients of the RI-CLPM [15] re-estimated with grade, gender, school location, and teaching method as time-invariant covariates and with poetry comprehension as a time-varying covariate (Section 4.6.6 of the main manuscript); the window means are those of Table 12. Panel B reports the autoregressive coefficients of the same model, Panel C reports the temporally ordered indirect associations together with the residual correlations between the mediator and outcome equations that would reduce each association to zero or make its 95% confidence interval include zero, and Panel D reports the largest residual correlations produced by the observed covariates.

Panel A. Covariate-adjusted within-person cross-lagged coefficients (standardized)

| **Path** | **T1 to T2** | **T2 to T3** | **T3 to T4** | **Mean** | **Main RI-CLPM mean (Table 7)** |
| --- | --- | --- | --- | --- | --- |
| GCL $\to$ Writing | 0.25*** | 0.16*** | 0.22*** | 0.21*** | 0.24*** |
| ECL $\to$ Writing | -0.22*** | -0.14** | -0.20*** | -0.19*** | -0.21*** |
| ICL $\to$ Writing | -0.16** | -0.10* | -0.13** | -0.13** | -0.16** |
| Writing $\to$ GCL | 0.14** | 0.07 | 0.12** | 0.11** | 0.13** |
| Writing $\to$ ECL | -0.07 | -0.03 | -0.07 | -0.06 | -0.07 |
| Writing $\to$ ICL | -0.05 | -0.01 | -0.03 | -0.03 | -0.04 |

Panel B. Covariate-adjusted within-person autoregressive coefficients (standardized)

| **Construct** | **T1 to T2** | **T2 to T3** | **T3 to T4** | **Mean** |
| --- | --- | --- | --- | --- |
| GCL | 0.31*** | 0.27*** | 0.33*** | 0.30*** |
| ECL | 0.26*** | 0.22*** | 0.29*** | 0.26*** |
| ICL | 0.35*** | 0.31*** | 0.36*** | 0.34*** |
| Writing | 0.44*** | 0.39*** | 0.45*** | 0.43*** |

Panel C. Temporally ordered indirect associations and residual-correlation thresholds

| **Indirect association** | **Estimate** | **95% CI** | **Residual correlation reducing the estimate to zero** | **Residual correlation making the 95% CI include zero** |
| --- | --- | --- | --- | --- |
| Cognitive load (t) $\to$ Schema construction (t + 1) $\to$ Writing (t + 2) | 0.152 | [0.106, 0.198] | 0.21 | 0.15 |
| Cognitive load (t) $\to$ Imagery processing (t + 1) $\to$ Writing (t + 2) | 0.121 | [0.080, 0.163] | 0.18 | 0.12 |
| Cognitive load (t) $\to$ Emotional resonance (t + 1) $\to$ Writing (t + 2) | 0.099 | [0.059, 0.139] | 0.14 | 0.08 |

Panel D. Largest residual correlation produced by each observed covariate

| **Observed covariate** | **Largest residual correlation with a mediator or outcome equation** |
| --- | --- |
| Teaching method | 0.09 |
| Poetry comprehension (time-varying) | 0.08 |
| Grade | 0.07 |
| School location | 0.05 |
| Gender | 0.03 |

*Note. **** $p$ *< .001, *** $p$ *< .01, ** $p$ *< .05. Fit of the covariate-adjusted RI-CLPM:* $\chi^{2}$*(87) = 395.7, CFI = 0.97, TLI = 0.93, RMSEA = 0.043, SRMR = 0.041. The indirect associations in Panel C are pooled over the windows T1 to T3 and T2 to T4. Each threshold in Panel C exceeds the residual correlation produced by every observed covariate in Panel D, so an unmeasured confounder would need to be more strongly related to the mediator and the outcome than any measured covariate to remove an indirect association.*

# **References cited in the supplementary materials**

The numbered references below follow the numbering used in the main manuscript.

[1] ANDERSON R C. The notion of schemata and the educational enterprise: General discussion of the conference. In: ANDERSON R C, SPIRO R J, MONTAGUE W E, eds. Schooling and the Acquisition of Knowledge. Hillsdale, NJ: Lawrence Erlbaum Associates, 1977: 415–431.

[2] AUSUBEL D P. Educational Psychology: A Cognitive View. New York: Holt, Rinehart & Winston, 1968.

[3] BATES D, MACHLER M, BOLKER B, et al. Fitting linear mixed-effects models using lme4. Journal of Statistical Software, 2015, 67(1): 1-48.

[5] BURKNER P C. brms: An R package for Bayesian multilevel models using Stan. Journal of Statistical Software, 2017, 80(1): 1-28.

[15] HAMAKER E L, KUIPER R M, GRASMAN R P P P. A critique of the cross-lagged panel model. Psychological Methods, 2015, 20(1): 102-116.

[18] KLEPSCH M, SCHMITZ F, SEUFERT T. Development and validation of two instruments measuring intrinsic, extraneous, and germane cognitive load. Frontiers in Psychology, 2017, 8: 1997.

[19] KOSSLYN S M. Image and Mind. Cambridge, MA: Harvard University Press, 1980.

[21] KUIKEN D, MIALL D S, SIKORA S. Forms of self-implication in literary reading. Poetics Today, 2004, 25(2): 171–203.

[22] KUZNETSOVA A, BROCKHOFF P B, CHRISTENSEN R H B. lmerTest package: Tests in linear mixed effects models. Journal of Statistical Software, 2017, 82(13): 1-26.

[32] ROSSEEL Y. lavaan: An R package for structural equation modeling. Journal of Statistical Software, 2012, 48(2): 1-36.

[33] SADOSKI M, PAIVIO A. Imagery and Text: A Dual Coding Theory of Reading and Writing. Mahwah, NJ: Lawrence Erlbaum Associates, 2001.
