# Supplementary material for: Construction of a hierarchical linear regression prediction model for classical Chinese poetry writing ability development
Source: PLoS One. 2026 Oct 30;21(10):e0361497. doi: 10.1371/journal.pone.0361497 (PMC13722217; doi:10.1371/journal.pone.0361497)
Supplement: Supplementary file 4 [file pone.0361497.s004.doc]

Reviewer #2:The authors have made substantial efforts to address the comments raised in the previous round of review. The revised manuscript is considerably clearer in its methodological description, including the specification of the four-level longitudinal structure, operationalization and psychometric evaluation of the study measures, documentation of the writing assessment and inter-rater reliability procedures, clarification of the prediction outcomes, and additional sensitivity analyses. These revisions have strengthened the manuscript considerably. However, two methodological issues raised in my previous review remain insufficiently resolved and prevent me from considering the manuscript fully technically and statistically sound at this stage.

1. Cross-validation and the hierarchical structure of the data.

My previous review raised concern regarding whether the cross-validation procedure adequately reflects the hierarchical structure of the data. The revised manuscript clarifies that the prediction model is evaluated using “10-fold cross-validation stratified by student ID and grade” and that predictions incorporate BLUP random-effect terms from the four-level model. However, it remains unclear what level of out-of-sample generalization the reported performance is intended to represent. Student-level splitting can retain classes and schools across training and validation folds and therefore evaluates prediction for students drawn from already represented higher-level contexts rather than prediction to entirely unseen classes or schools. Please clarify the intended prediction target. If the reported performance is intended to support generalization beyond the sampled classes and schools, an additional cluster-aware validation analysis, such as leave-class-out and/or leave-school-out validation, would be more appropriate. The authors should also clarify how BLUPs were obtained within each validation fold and confirm that outcome information from validation observations was not used in estimating the corresponding random effects.

Response:

We thank the reviewer for this comment. Three prediction targets are now distinguished, each evaluated with its own cross-validation design, and the fold-wise computation of the BLUPs and the exclusion of validation outcomes from every random-effect prediction are stated explicitly.

(1) Intended prediction target and cluster-aware validation designs. Section 3.6 states that the predictive performance of a multilevel model depends on which units are unseen at the time of prediction, and that student-level splitting supports generalization only to new students drawn from higher-level contexts already represented in the training folds. Three prediction targets are therefore distinguished. Design A (student-grouped 10-fold cross-validation) evaluates new students within the sampled classes and schools and is the confirmatory predictive criterion of O6; Design B (leave-one-class-out cross-validation over all 48 classes) evaluates students in entirely unseen classes within the sampled schools; and Design C (leave-one-school-out cross-validation over all 12 schools, holding out the four classes and 160 students of a school together) evaluates students in entirely unseen schools. Designs B and C are the leave-class-out and leave-school-out validation analyses suggested in the comment, and they are reported without acceptance thresholds because O6 does not preset criteria for those two targets; Figure 6A summarizes the three designs and the random effects that enter each prediction. Table 8 (Section 4.5.1) reports R² = 0.847 (95% CI [0.834, 0.859]), RMSE = 4.28, and 80.3% three-band accuracy under Design A (the preset RMSE target is met, whereas the 85% accuracy target is not), R² = 0.768 and RMSE = 5.24 under Design B, and R² = 0.656 and RMSE = 6.38 under Design C. Fold-level, class-level, and school-level metrics for the three designs are provided in Supplemental Table S8.

(2) How BLUPs are obtained within each validation fold. Section 3.6 formalizes the BLUP computation within each fold and states, for each of the three designs, from which training-fold observations the class-level and school-level BLUPs are estimated. In Design A, the class and school BLUPs of a validation student are estimated jointly from the training-fold observations of that student’s classmates and of the other three classes of that school, and the four measurement waves of the validation student are excluded from both. In Design B, the class BLUP is unavailable because every observation in the held-out class is held out, and the school BLUP is estimated from the training-fold observations of the other three classes of that school. In Design C, every class and school random effect of the held-out school is set to zero because no training-fold observation from that school is available for either BLUP. Equations (19) to (21) give the prediction expression of each design, and Section 4.5.1 restates this rule before the predictive results are reported.

(3) Exclusion of validation outcome information from BLUP estimation. The BLUP expression b̂^(−f) = Ĝ Z′_(−f) V̂_(−f)^(−1) (Y_(−f) − X_(−f) γ̂^(−f)) in Section 3.6 makes explicit that the outcomes, fixed effects, and variance components that enter every BLUP are computed within each training fold from the training-fold outcomes, design matrices, and variance components alone, so outcome information from the validation fold enters neither the fixed effects, the variance components, nor any random-effect prediction. Algorithm 1 (Section 3.5, step 7) outlines the fold-wise pipeline, and Supplemental Table S7 enumerates its twelve data-dependent steps: multiple imputation with predictive mean matching (m = 20), Level-1 within-student centering, Level-2 to Level-4 grand-mean centering, growth-order and random-effects reselection, REML refitting, class-level and school-level BLUP estimation, grade-normed band-cutoff derivation, band-probability derivation, leave-one-covariate-out refits, and averaging over the 20 imputed datasets are all carried out on the training folds and applied unchanged to the validation folds, and validation observations without an observed outcome are excluded from the performance metrics. Supplemental Table S7 also records for every step whether validation outcomes were used: the column returns “No” for every estimation step and “Scoring only” for the three steps that score out-of-fold predictions (step 9, leave-one-covariate-out importance; step 11, performance metrics; step 12, the bootstrap interval of R²).

2. Number of Level-4 units and stability of school-level estimates.

The revised four-level specification resolves the previous ambiguity concerning the hierarchical structure by explicitly modeling measurement occasions within students, students within classes, and classes within schools. The manuscript also now reports several useful robustness analyses. Nevertheless, the highest level contains only 12 schools. The current robustness analyses address missing-data handling, outliers, centering, and alternative model specifications, but they do not directly establish the stability of Level-4 variance components and school-level or cross-level estimates with only 12 Level-4 units. The manuscript cites the relative robustness of Bayesian estimation with small higher-level cluster counts, but this does not by itself demonstrate stability in the present dataset. I therefore recommend an analysis or justification specifically addressing the sensitivity of the principal Level-4 and cross-level results to the limited number of schools.

Response:

We thank the reviewer for this important comment. The manuscript no longer relies on the general robustness of Bayesian estimation to small higher-level cluster counts as evidence of stability in the present data. Section 4.6.5 states that this general robustness is by itself insufficient to establish the stability of the Level-4 variance component and of the school-level and cross-level coefficients in the present dataset, and that each of these estimates was therefore examined with five dedicated analyses, which are specified in Section 3.5 and each target a distinct aspect of small-cluster inference:

(a) Twelve leave-one-school-out (LOSO) refits, each of which drops one school and re-estimates the four-level model on the remaining 11 schools, quantifying the influence of every individual school on each estimate; complete LOSO estimates are reported in Supplemental Table S10.

(b) A school-level wild cluster bootstrap using Webb weights (B = 9,999) for the coefficients, which adjusts the interval width for the small number of clusters without relying on the asymptotic Wald standard errors that are unreliable under these conditions, and a school-level case bootstrap for the school-level variance component and ICC_school.

(c) Bayesian re-estimation under three alternative priors on the random-effect standard deviations (half-Cauchy(0, 5), half-Student-t(3, 0, 10), and Uniform(0, 30)) in addition to the reference inverse-gamma prior, which characterizes the prior sensitivity of the Bayesian estimates; complete prior-sensitivity results are reported in Supplemental Table S11.

(d) A school fixed-effects specification that removes the between-school variance component entirely for the coefficients that vary within schools.

(e) A calibrated Monte Carlo simulation of 1,000 datasets generated from the fitted model with the observed 12-school design, which returns the empirical bias, sampling variability, and interval coverage that the inference procedures actually deliver under that design; complete simulation results are reported in Supplemental Table S12.

Table 11 (Section 4.6.5) collects the results of all five analyses for the six principal estimates: the school-level intercept variance τ_v000, ICC_school, Teaching × CogLoad, Grade × CogLoad, Environment × CogLoad, and the moderated-mediation index. Figure 12 displays the leave-one-school-out range of each estimate together with its Kenward-Roger, profile-likelihood, or Monte Carlo interval and its bootstrap interval, scaled to the main estimate. A coefficient is classified as Stable when its sign is unchanged in all 12 LOSO refits, its Kenward-Roger and wild-bootstrap 95% intervals exclude zero, its 95% credible interval excludes zero under all four priors, and the calibrated simulation returns nominal-95% coverage of at least 0.925, and a variance component or ICC whose intervals reach the boundary of the parameter space is classified as Imprecise (note to Table 11).

Applying these criteria separates the six estimates into three groups (Section 4.6.5). The Teaching × CogLoad interaction (γ = 0.47, LOSO range [0.39, 0.56], wild-bootstrap 95% CI [0.11, 0.83], simulated coverage 0.945) and the moderated-mediation index (0.07, LOSO range [0.04, 0.10], wild-bootstrap 95% CI [0.02, 0.12], simulated coverage 0.930) satisfy every criterion and remain interpretable under the 12-school design. The school-level intercept variance (τ_v000 = 10.03) and ICC_school (0.083) remain positive in all 12 LOSO refits (ranges [6.21, 14.88] and [0.053, 0.118]), and the boundary-corrected likelihood-ratio test supports a non-zero school variance (χ̄² = 3.53, p = .030); however, their 95% profile-likelihood intervals ([0.00, 38.69] and [0.000, 0.260]) and school-level bootstrap intervals ([0.00, 20.28] and [0.000, 0.160]) reach the boundary of the parameter space, the simulation shows that the school-level case bootstrap undercovers (0.818) whereas the profile-likelihood interval is close to nominal (0.959), and the likelihood-ratio test detects a school variance of this size in only 57% of the simulated datasets (Supplemental Table S12). Both are therefore classified as Imprecise: the school level is retained because it reflects the sampling design and protects the standard errors of the class-level and cross-level coefficients, and the magnitude of school-level clustering is not interpreted (Section 4.3.1; Section 5.1; Section 5.3). Table 6 (Section 4.3.1) reports the asymptotic SEs of the variance components for reference only, together with boundary-corrected likelihood-ratio statistics and 95% profile-likelihood and parametric bootstrap intervals, in place of the Wald z statistics, and Section 4.6.3 reports that the four-level specification is retained over a three-level alternative by the boundary-corrected likelihood-ratio test (χ̄² = 7.9, p = .034) and on design grounds, although AIC barely separates the two models and BIC favours the three-level model. The Grade × CogLoad (γ = 0.39) and Environment × CogLoad (γ = 0.31) interactions fail the wild-bootstrap and coverage criteria and are prior-sensitive; they are therefore marked Exploratory in Table 9 and are not used as support for instructional recommendations at Level 4. Complete results are provided in Supplemental Tables S10 (leave-one-school-out refits), S11 (prior sensitivity), and S12 (simulation), and the analytic registry in Supplemental Table S9 records the pre-specified stability criteria and the resulting classification (Stable, Exploratory, or Imprecise) of every Level-4 and cross-level estimate. The Limitations (Section 5.3) acknowledge that the highest level comprises only 12 schools, which limits precision at Level 4 and for cross-level estimates, and the Conclusion (Section 6) lists designs with more schools as the natural next step for sharpening Level-4 inference.

Overall, many of my previous concerns have been satisfactorily addressed, and the manuscript has improved substantially. My remaining concerns are considerably narrower than in the previous round. However, because they bear directly on the interpretation of the predictive validation and the reliability of inference at the highest level of the hierarchical structure, I consider them substantive rather than merely editorial. Addressing these points would strengthen the statistical support for the manuscript's conclusions.

Reviewer #3:Despite an interesting topic‚ a multi-year longitudinal dataset and advanced statistical analyzes‚ some issues need clarification․ The mapping of NASA-TLX dimensions onto intrinsic‚ extraneous and germane cognitive load should be seen as preliminary and should be interpreted with caution․ Interpretative assumptions are strong․ Since all analyzes (RI-CLPM‚ mediation) are based on observational data‚ it is wise to avoid overstating possible directional and causal claims․ The authors should clarify whether the multiple imputations‚ preprocessing‚ fitting of the model‚ and estimation of the BLUP were done separately in each cross-validation fold‚ to avoid accidental data leaks․ Since there are many tests and predictions conducted‚ the authors should clarify which tests are confirmatory and which are exploratory‚ and not overextend their conclusions or recommendations beyond what their results support․

Response:

We thank the reviewer for the careful reading. The four points are addressed explicitly throughout the manuscript, and interpretive statements are kept within the limits that the observational design and the provisional TLX mapping support.

(1) NASA-TLX to ICL/ECL/GCL mapping. Section 3.3 presents the mapping as provisional under three explicit assumptions, notes that the ECL indicator is the least direct link because perceived performance also reflects self-efficacy and task success, states that germane load is now regarded as the working-memory resources devoted to intrinsic load rather than as an independent additive load, and cautions that the labels ICL, ECL, and GCL denote TLX-indexed indicators throughout the article, so results that depend on their distinction are interpreted as preliminary. Section 4.6.4 reports four dedicated measurement analyses: competing measurement models of the TLX subscales at every wave, configural, metric, and scalar invariance across Grades 3 to 6, a multitrait-multimethod comparison with the Klepsch et al. instrument in a stratified 20% subsample (n = 384) at T3, and a sensitivity model with a single undifferentiated load score. The three-factor model fits best at every wave, invariance holds across grades, every monotrait-heteromethod correlation exceeds its heterotrait-monomethod counterparts, and Klepsch-based re-estimation preserves the signs of the three load effects, each within 0.05 of its NASA-TLX counterpart (ICL β = −0.37, ECL β = −0.47, GCL β = 0.48 versus −0.41, −0.49, 0.45), with a mediation index of 0.56 (95% CI [0.47, 0.65]) indistinguishable from 0.587. The undifferentiated load score removes the teaching-method interaction (γ = 0.09, p = .53), so the O5 finding depends on the load-type distinction and is interpreted with the same caution. Supplemental Table S6 provides the full results. The Discussion (Section 5.1) and the Limitations (Section 5.3) restrict any interpretation of the load-component differentiation to the provisional-mapping caveat, and Table 13 embeds the caveat in the O1 implication row.

(2) Directional and causal claims. All analyses based on observational data are stated as statistical associations rather than causal effects. Section 2.5 states that because the predictors, mediators, and outcome are observed rather than manipulated, the indirect effects are interpreted as statistical indirect associations under the hypothesized ordering rather than as demonstrated causal mechanisms. The RI-CLPM analysis (Section 4.3.5) reports within-person lagged associations rather than causal effects, and the mediation analysis (Section 4.4.1) reports statistical indirect associations under the hypothesized ordering rather than demonstrated causal mediation. Section 4.6.6 further examines the assumed ordering with three analyses: re-estimating the RI-CLPM with grade, gender, school location, and teaching method as time-invariant covariates and poetry comprehension as a time-varying covariate (Supplemental Table S13, Panel A); a temporally ordered mediation model from cognitive load at one wave through each mediator at the next wave to writing ability at the wave after (T1 to T3 and T2 to T4, Panel C); and the residual mediator-outcome correlation that would eliminate each temporally ordered indirect association. The three thresholds (0.21, 0.18, and 0.14) exceed the largest residual correlation produced by any measured covariate (0.09 for teaching method, Panel D), so an unmeasured confounder would need a stronger residual association than any measured covariate to remove an indirect association. Section 5.3 lists the observational design and the impossibility of ruling out unmeasured confounders such as shifts in reading habits or parental involvement as an explicit limitation, and the Conclusion (Section 6) restricts instructional recommendations to the confirmatory findings that meet the preset acceptance criteria under Holm familywise error control.

(3) Multiple imputation, preprocessing, model fitting, and BLUP estimation within each cross-validation fold. Section 3.6 states that all data-dependent steps are carried out within folds; Algorithm 1 (Section 3.5, step 7) outlines the fold-wise pipeline, and Supplemental Table S7 enumerates its twelve steps. Multiple imputation with predictive mean matching (m = 20) is estimated on the training folds only, validation outcomes are neither used in the imputation models nor imputed, and validation observations without an observed outcome are excluded from the performance metrics. Level-1 predictors are centered on each student’s own mean, and Level-2 to Level-4 predictors on training-fold grand means. The growth order and random-effects structure are reselected in each training fold by AIC/BIC, and the four-level model is re-estimated by REML in the training fold. BLUP estimation is confined to the training-fold outcomes, design matrices, and variance components, as detailed in Section 3.6 (lines 404-430) and in item (3) of the response to Reviewer #2, Comment 1 above. Band cutoffs, band probabilities for ROC-AUC, and leave-one-covariate-out refits are also confined to the training folds, and predictions are averaged over the 20 imputed datasets within each fold. Supplemental Table S7 documents the validation-outcome status of every step and returns “No” for every estimation step and “Scoring only” for the three steps that score out-of-fold predictions (steps 9, 11, and 12).

(4) Confirmatory versus exploratory tests. Section 2.5 designates four hypotheses as confirmatory: the three TLX-indexed load-component associations of O1, the three indirect effects of O3, the interaction of cognitive load with teaching method of O5, and the quadratic growth term of O6. These eight tests form one family whose p values are adjusted with the Holm procedure, and predictive performance for new students within the sampled classes and schools (Design A) serves as the confirmatory predictive criterion of O6. All remaining analyses are exploratory under Benjamini-Hochberg control within their own family and generate hypotheses rather than support conclusions or instructional recommendations. Table 9 (Section 4.5.2) attaches an explicit inferential tier (Confirmatory (Holm) or Exploratory (BH)) and both the raw and multiplicity-adjusted p values to every path. Table 9 records that the eight confirmatory tests retain significance under Holm control, that O6 is partially supported (RMSE 4.28 meets the 5.0-point threshold, whereas three-band accuracy of 80.3% falls short of the 85% target, so predicted bands are recommended for monitoring rather than placement decisions), and that the O2 and O4 paths, the Grade × CogLoad and Environment × CogLoad interactions, and the non-significant analyses of Section 4.5.3 are exploratory. The Discussion (Section 5.1) and Table 13 restrict the instructional and curricular implications to the confirmatory findings, and the Grade 4 ECL peak, the exploratory Grade × CogLoad and Environment × CogLoad interactions, and the exploratory O2 and O4 results are explicitly reported as hypothesis-generating extensions to be re-examined before informing instructional recommendations. Supplemental Table S9 provides the analytic registry that records the pre-specification, family membership, tier, and adjusted p value of every inferential test.
