# Supplementary material for: Construction of a hierarchical linear regression prediction model for classical Chinese poetry writing ability development
Source: PLoS One. 2026 Oct 30;21(10):e0361497. doi: 10.1371/journal.pone.0361497 (PMC13722217; doi:10.1371/journal.pone.0361497)
Supplement: Supplementary file 3 [file pone.0361497.s003.docx]

**Reviewer 1**

The manuscript addresses a genuinely under-studied question — the cognitive-load mechanisms linking classical poetry instruction to writing-skill development — with an ambitious multilevel longitudinal design. The review below is organized in two parts. Section 2 covers issues of basic verifiability (documentation, citations, data availability) that I would encourage the editor to weigh independently of the authors' scientific response. Section 3 covers substantive concerns about measurement, analytic design, and interpretation that bear on whether the paper's central claims are adequately supported as written.

**1. Summary of the Submission**

The authors report a one-year, four-wave longitudinal study of 1,920 students (grades 3–6, 48 classes, 12 schools) examining how three components of cognitive load (intrinsic, extraneous, germane) predict classical-poetry-linked writing ability, using three-level hierarchical linear models (student/class/school). The paper reports differentiated effects of the three load types, mediation via schema construction, imagery processing and emotional resonance (total indirect effect = 0.537), a cross-level moderation by teaching method, a quadratic growth trajectory for writing ability, and a final "prediction model" said to achieve 88.6% accuracy. All six pre-registered modeling objectives (O1–O6) are reported as supported.

Response: We thank the reviewer for the accurate summary of our submission. In the revised manuscript, we have refined the description of the modeling framework as a four-level hierarchical linear model (measurement occasions nested within students, students within classes, and classes within schools) rather than three-level, to correctly reflect the nesting of four measurement waves within each student (Section 3.5). We have also distinguished the two complementary metrics of the predictive pipeline, namely R² = 0.786 with RMSE = 4.82 for the continuous regression task and 88.6% overall accuracy for the derived three-band classification task (Abstract, Section 4.5, Fig. 10 caption).

**2. Major Concerns — Verifiability and Documentation**

**2.1 Ethics documentation is internally inconsistent**

The submission's Ethics Statement field (page 3 of the cover materials) is entered as "N/A", which under PLOS ONE policy should only be used when the study did not involve human participants. Yet the manuscript body states unambiguously that "This study has been approved by the Ethics Committee of Heze University" and references written informed consent. This is a direct contradiction that the editorial office will need resolved regardless of the scientific review: the authors must supply the IRB/ethics committee name, an approval number, and clarify the consent procedure used for minors (parental/guardian consent should be specified, not just student assent, given participants are grade 3–6 children).

Response: Thank you for your detailed review. We have provided more detailed ethical approval information in the sections '3.2 Research participants and sampling' and 'Ethics Declaration', including the ethical approval number and date. Additionally, the process of obtaining written informed consent from the legal guardians (parents) of research participants is detailed in the 'Consent to Participate' section.

2.2 Widespread mismatch between in-text citation numbers and the reference list

The reference list (Section "References") is alphabetized by first-author surname, but the in-text bracketed citation numbers do not appear to have been updated to match — the numbers instead follow what looks like an original by-order-of-appearance numbering. This is not a one-off typo; the pattern is consistent across at least twelve checked citations. A sample of the mismatches:

This needs a full, careful renumbering pass (or restoration of consistent citation-manager linking) before the reference list can be trusted; as it stands, essentially every in-text citation points to the wrong source, which undermines the traceability of every claim attributed to prior literature.

Response: Thank you for your careful review and examination. We have adjusted the reference order in the "References" section according to the descriptions in the main text.

**2.3 Data-availability link could not be verified**

The Data Availability statement points to a GitHub repository (github.com/a18907052542-lang/Chinese-Poetry-Writing-Ability-Development). I was unable to locate this repository or its owner through search, and direct access was blocked. This may simply reflect a private, unindexed, or very recently created repository — but given PLOS ONE's requirement that the minimal dataset be openly and fully available at submission, I'd ask the editor/authors to confirm the repository is public, resolves correctly, and actually contains the raw or minimal dataset (not just code) underlying every reported analysis, including item-level scale responses, before this claim is accepted at face value.

Response: We thank the reviewer for the concern. We have now verified the repository is publicly and openly accessible and have expanded the Data Availability statement to explicitly enumerate the six categories of materials archived in the repository. These include: (a) item-level raw data for 1,920 students by 4 waves across 19 xlsx sheets; (b) codebook with variable definitions and coding schemes; (c) complete R and Python analysis pipeline; (d) the three measurement instruments in Chinese adaptation (Cognitive Load Scale NASA-TLX adaptation, Poetry Comprehension Test, Writing Ability Scoring Rubric); (e) 16 anonymized representative composition samples; and (f) session metadata log. A snapshot of the repository has additionally been deposited on Zenodo with a citable DOI to provide a permanent archival identifier (see revised Data Availability).

**2.4 The "prediction model" and Algorithm 1 are underspecified and partly inconsistent with the**

**HLM described in Section 3.5**

Section 3.5 specifies REML estimation for the core multilevel models, and Bayesian/MCMC estimation "for complex models." Algorithm 1 then introduces a full machine-learning-style pipeline (10-fold stratified cross-validation, multiple imputation with 20 chains, 4 HMC chains × 10,000 iterations, leave-one-covariate-out feature importance) that is not clearly reconciled with the earlier description. Several concrete questions the authors should answer:

● What specific algorithm generates the point predictions in Fig. 10A (linear mixed-model fixed effects + BLUPs, or a separate learner such as random forest/gradient boosting)? The manuscript's prose suggests the former, but the ROC/AUC classification analysis in Fig. 10C implies a categorical classifier, and the two are never formally connected.

● The Abstract and Conclusion report "88.6% accuracy," while Section 4.5 separately reports R^2^

= .786 and RMSE = 4.82 for a continuous outcome. These are different quantities describing different modeling tasks (regression vs. three-band classification) and should not be conflated as a single headline number.

● No modeling software is named anywhere (R/lme4/nlme, Mplus, HLM7, SPSS, Python/statsmodels, etc.). This is necessary for reproducibility and for judging whether the described REML, Bayesian/MCMC, and cross-validation steps were actually run in the same pipeline.

Response: We have addressed all three concrete questions raised by the reviewer. First, we now explicitly clarify that the point predictions in Fig. 10A are BLUP-augmented fixed-effect predictions from the full four-level linear mixed model, formally Y_hat_tijk = X_tijk * beta_hat + Z_tijk * b_hat_ijk, not from an independently trained learner such as random forest or gradient boosting. Second, we now clearly separate the two complementary metrics of the predictive pipeline: R² = 0.786 (RMSE = 4.82) belongs to the continuous-outcome regression task, while 88.6% overall accuracy belongs to the three-band classification task derived post-hoc by discretizing the same regression predictions using grade-normed 30th and 70th percentile cutoffs. Both metrics are now reported side-by-side in the Abstract, Section 4.5, Fig. 10 caption, and Conclusion. Third, we have named all modeling software: R version 4.3.2 with lme4 and lmerTest for REML estimation, brms with cmdstanr backend for Bayesian estimation of the moderated-mediation model, lavaan for CFA, and Python 3.11 with statsmodels and scikit-learn for the prediction pipeline.

**2.5 Construct mapping for the cognitive-load instrument is not justified**

Cognitive load is measured with "an adapted NASA-TLX scale" whose three subscales are labeled Mental Effort, Task Difficulty, and Performance/self-efficacy (the manuscript uses both labels inconsistently — compare Section 3.3 text to Fig. 5A). The NASA-TLX was designed as a general workload inventory, not as an operational measure of Sweller's tripartite intrinsic/extraneous/germane distinction, and "Performance" is conceptually an outcome rather than a load. The authors should justify, ideally with a citation or their own validation evidence beyond the reported CFA fit indices, why these three subscales should be interpreted as ICL, ECL, and GCL respectively, rather than as three general facets of perceived workload.

Response: We have added a theoretical justification for the mapping from NASA-TLX subscales to Sweller's ICL/ECL/GCL distinction in Section 3.3. Specifically, the Task Difficulty subscale is interpreted as ICL because its items reference character density, syntactic complexity, and semantic distance of the material itself; the Performance/Self-efficacy subscale is reverse-scored and interpreted as ECL following the operationalization convention of Paas and van Merrienboer (2020, reference [28]); and the Mental Effort subscale is interpreted as GCL, consistent with the mental-effort formulation of germane processing in Klepsch et al. (2017, reference [18]). We also inconsistency between text and Fig. 5A labels resolved by consistently using "Performance/Self-efficacy" throughout. Additionally, we conducted an empirical convergent-validation study on a stratified 20% subsample (n = 384) at wave T3 using the Klepsch et al. three-factor cognitive load scale (Chinese adaptation). Disattenuated correlations between corresponding constructs were r = 0.69 for ICL, r = 0.73 for ECL, and r = 0.71 for GCL (all p < .001); regression coefficients from the Klepsch-based model preserved the sign and rank order of all three cognitive-load effects and lie within a 0.05 tolerance bound of the NASA-TLX-based estimates (see new Section 4.6.4).

**2.6 Uniformly confirmatory results across all six modeling objectives**

Table 6 reports that all six a priori objectives (O1–O6), spanning 16 separate parameter estimates, are "Supported," the great majority at p < .001. This is an unusually clean pattern for an exploratory, multi-path multilevel model with this many free parameters, and it is worth the authors explicitly addressing: were any hypothesized paths, subgroup contrasts, or robustness checks non-significant or in an unexpected direction at any stage of model-building? A brief account of what did not work as expected (or a statement that truly nothing did) would strengthen confidence in the reported effects.

Response: We have added a new subsection 4.5.3 ("Non-significant and directionally unexpected findings during model building") to document candidly the non-significant and directionally unexpected results encountered during the model-building process. Specifically, we report: (1) three non-significant paths that were not promoted to primary results, including ICL to Vocabulary Richness (beta = -0.09, p = 0.11), School Type by Grade interaction on trajectory intercepts (gamma = 0.14, p = 0.23), and Emotional Resonance by ECL interaction on writing outcomes (gamma = 0.10, p = 0.18); (2) a directionally unexpected finding preserved across robustness checks, namely the Grade 4 ECL peak (-0.52) rather than the initially expected Grade 3 peak, which we interpret as reflecting the transitional pedagogy of Grade 4 in the Chinese primary curriculum; and (3) a non-significant urban versus rural comparison on the mediation index (Delta = 0.03, p = 0.29), and a pre-planned mediation via prosodic fluency that was dropped after the pilot revealed unstable factor structure (Cronbach's alpha = 0.62, chi-square/df > 4).

**2.7 Framing of Table 8 ("Engineering contribution," "deployment pipeline") is misaligned with**

**the journal and audience**

Table 8 and the surrounding text describe findings using software/ML-engineering vocabulary — "fixed-effect priors reusable in downstream models," "regression anchors," "deployment pipeline," "grade-conditional gain table for adaptive model deployment." For a PLOS ONE education/psychology readership, this framing obscures rather than clarifies the pedagogical implications and reads as inconsistent with the rest of the paper's register. I'd recommend rewriting this table and the surrounding discussion in terms of instructional and curricular implications (e.g., what a classroom teacher or curriculum designer should change), which is presumably the intended contribution.

Response: We have completely rewritten the table and its surrounding discussion, and it is now presented as Table 10 ("Instructional and curricular implications for each modeling objective (O1 to O6)"). The two right-hand columns are renamed "Theoretical significance" and "Curriculum implications", and all engineering vocabulary (fixed-effect priors, downstream models, regression anchors, deployment pipeline, grade-conditional gain table) has been replaced with pedagogically oriented language addressed to classroom teachers and curriculum designers. Concrete examples include: for O1, teachers distinguish three sources of load (simplify displays to reduce ECL, stage difficulty to manage ICL, use why-questions to enlarge GCL); for O3, activate all three mediating channels through structural questions, mental imagery prompts, and emotional inquiry; for O5, prioritize interactive multimedia in Grades 3 to 4 and transition to inquiry-oriented instruction in Grades 5 to 6.

**3. Major Concerns — Content, Measurement, and Analytic Validity**

The items below concern the science itself: whether the instruments, design, and reported statistics can support the paper's claims as written, independent of the documentation issues in Section 2.

**3.1 The three mediators are never operationalized in the Methods**

Section 3.3 describes instruments for exactly three constructs: the Cognitive Load Scale, the Poetry Comprehension Test, and the Writing Assessment. Section 4.4 then reports full mediation results — including specific indirect effects (0.218, 0.175, 0.144) and Monte Carlo confidence intervals — for three additional constructs: schema construction, imagery processing, and emotional resonance. No scale, item count, reliability coefficient, or scoring procedure is given anywhere for these three mediators. This needs to be corrected before the mediation analysis (arguably the paper's central contribution, at 74.8% of total effect) can be evaluated at all.

Response: We thank the reviewer for identifying this critical omission. We have added a dedicated description of the three mediator instruments in Section 3.3. Schema Construction was measured via the Poetry Schema Construction Task (PSCT), 6 open-response items across three subtasks, anchored in classical schema theory (Anderson, 1977, reference [1]) and Ausubel's meaningful learning theory (reference [2]); Cronbach's alpha = 0.84, two-rater ICC(2,1) = 0.87. Imagery Processing was measured via the Imagery Generation and Verification Task (IGVT), 16 items adapted from Kosslyn (1980, reference [19]) mental imagery paradigm and Sadoski and Paivio (2001, reference [33]) dual coding framework; Cronbach's alpha = 0.81, ICC(2,1) = 0.84. Emotional Resonance was measured via the Poetry Emotional Resonance Scale (PERS), 12 Likert items plus 4 open-response items adapted from Kuiken et al. (2004, reference [21]) Literary Response Questionnaire; Cronbach's alpha = 0.86, ICC(2,1) = 0.83. The three instruments were administered at each of the four measurement waves (T1 to T4) immediately following the poetry comprehension test and prior to the writing task. Reliability and validity indices for the three instruments have also been added to Table 4. Full item pools, scoring rubrics, and rater training materials are archived as Supplemental Appendices S2 (PSCT) , S3 (IGVT), and S4 (PERS) in the project GitHub repository.

**3.2 Discriminant validity between predictors, mediators, and outcome is not demonstrated**

Writing ability is scored on six dimensions including thematic coherence and creative expression (Fig. 5C); the mediators are schema construction, imagery, and emotion; and the poetry comprehension test spans literal/inferential/critical/creative levels (Fig. 5B). These instruments plausibly share conceptual and item content, which would mechanically inflate both the Table 3 correlations and the reported 74.8%-of-total-effect mediation — an unusually complete mediation for a psychological process. I'd ask the authors to report discriminant validity (e.g., HTMT ratios, or at minimum the inter-construct correlations from the measurement model) between the mediators, the poetry comprehension test, and the writing outcome, rather than the CFA fit indices for cognitive load and writing alone.

Response: We have added a new subsection 4.2.3 ("Discriminant validity among core constructs") reporting three complementary discriminant-validity analyses. First, HTMT ratios (Henseler et al., 2015, reference [16]) for all pairwise combinations of the seven core constructs are all below the 0.85 conservative threshold. The highest HTMT was observed between Schema Construction and Poetry Comprehension (0.72), which is theoretically expected. HTMT values between the three mediators and the writing outcome ranged from 0.61 (Emotional Resonance) to 0.70 (Schema Construction), confirming meaningful shared variance while remaining well below the tautological threshold. The full HTMT matrix is presented in new Table 5. Second, the Fornell-Larcker criterion was satisfied: square root of AVE for Schema was 0.79 (max off-diagonal r = 0.65); for Imagery 0.76 (0.62); for Emotion 0.74 (0.58); for Writing 0.76 (0.68). Third, an exploratory factor analysis with promax rotation showed no cross-loading was greater than or equal to 0.40, indicating item-level distinctness.

**3.3 Causal language exceeds what the design supports**

The models are concurrent/longitudinal mean-level associations, not cross-lagged panel models. That supports describing how cognitive load and writing ability co-develop over the year, but not the causal language used throughout ("cognitive load ... predicted writing ability," "GCL positively predicted it"). A plausible reverse pathway — weaker writers experiencing more load, or stronger poetry processors reporting less load — is never raised or ruled out. I'd recommend either a cross-lagged analysis establishing temporal precedence, or systematically softening the causal language to "was associated with" / "forecast."

Response: We adopted the reviewer's first recommendation and added a random-intercept cross-lagged panel model (RI-CLPM; Hamaker et al., 2015, reference [15]) as a new subsection 4.3.5 with results in the new Table 7. The RI-CLPM results provide partial temporal-precedence support for the causal direction assumed in the main model. The lagged path from GCL to Writing was consistently significant across three lags with mean beta = 0.22 (p < .001), while the reverse path from Writing to GCL was significant but smaller (mean beta = 0.12, p = .007). The lagged path from ECL to Writing was significantly negative (mean beta = -0.19, p < .001) while the reverse path was non-significant, consistent with a substantively unidirectional hindering association. ICL exhibited a similar unidirectional pattern. In addition, we systematically softened causal language throughout the manuscript to "longitudinally associated with" wherever the RI-CLPM directional dominance was not unambiguous; see revised Abstract, Section 4.4, Section 5.1, and Conclusion.

**3.4 Self-report cognitive-load measurement in grade 3–6 children**

The adapted NASA-TLX is an introspective self-report instrument, collected here from children as young as ~8 years old. Whether children this age can reliably introspect on and differentiate intrinsic, extraneous, and germane load — a distinction that is subtle even for adult learners — is a construct-validity question the manuscript does not address, and it compounds the NASA-TLX-mapping concern raised in Section 2.5.

Response: We have added a dedicated paragraph at the end of Section 2.2 reviewing the developmental and psychometric evidence supporting self-report cognitive-load differentiation in the Grade 3 to Grade 6 age range. Two lines of evidence are presented. First, developmental research (Roebers, 2017, reference [31]) indicates that metacognitive monitoring and executive function undergo substantial refinement between ages 7 and 12, with children in this window becoming capable of introspective reports on specific, task-anchored cognitive experiences. Second, cognitive load theory has been progressively operationalized for younger learners: Leppink and van den Heuvel (2015, reference [23]) and Klepsch et al. (2017, reference [18]) developed and validated a three-factor cognitive load scale in adolescent samples (Cronbach's alpha = 0.75 to 0.83; chi-square/df = 2.14, CFI = 0.94, RMSEA = 0.052). Consistent with these findings, our items were anchored to the immediately preceding poetry learning task and phrased in age-appropriate language during pilot testing with 120 Grade 3 to 6 students. The empirical convergent-validation with the Klepsch instrument (Section 4.6.4) further supports the construct validity of self-reported cognitive-load differentiation at these grade levels.

**3.5 ICC / variance-decomposition formulas appear internally inconsistent**

Equations (11)–(12) define the class and school ICCs using only τ_00_ (class intercept variance) and τ_11_ (defined in Section 3.5 as class-level slope variance) — the school-level variance term ψ_00_ from equation (5) never enters either formula. Yet Table 5's reported "school ICC" of 0.318 numerically matches (τ_00_ +ψ_00_)/total rather than what equation (12) actually specifies. Either the printed formula is wrong or the notation in Section 3.5 and Table 5 do not correspond — this should be confirmed directly with the authors rather than assumed to be a typo.

Response: We have corrected the ICC formulation and clarified the notation. Because the model has four levels, the ICC is now specified with all variance components explicitly included. Specifically, let sigma-squared denote the within-student residual variance (Level 1), tau_r0 the between-student variance in the intercept (Level 2), tau_u00 the between-class variance (Level 3), and tau_v000 the between-school variance (Level 4). Two ICC quantities are of primary interest: ICC_class = tau_u00 / V_total = 0.235 (Level 3 dependency) and ICC_school = tau_v000 / V_total = 0.083 (Level 4 dependency). The value 0.318 previously labeled as "school ICC" is the cumulative higher-level dependency (tau_u00 + tau_v000) / V_total, indicating the proportion of total variance attributable to differences at the class level and above. This is now made explicit in the revised Note to Table 6 (the former Table 5).

**3.6 Ambiguity between a three-level and a four-level model**

Equation (1) already embeds Time and Time2 at "Level 1 (student)," collapsing occasions into the student level, while the growth-curve section (eq. 8–10) treats time as effectively its own level. With four waves nested in students nested in classes nested in schools, this could reasonably be framed as a four-level model. It matters for interpretation: does the 68.2% "student-level" variance in Table 5 include within-student change over the year, or only stable between-student differences? The manuscript is not consistent enough on this point to tell.

Response: We have explicitly reframed the model as four-level throughout the manuscript. In Section 3.5, we now specify that repeated measurement occasions (Level 1, four waves per student) are nested within students (Level 2), students within classes (Level 3), and classes within schools (Level 4). Making Level 1 explicit resolves the ambiguity: the student-level residual variance reported in the old three-level null model (68.2%) is now decomposed into two conceptually distinct components: within-student variance across measurement occasions (Level 1, σ² = 40.3%) and stable between-student baseline differences (Level 2 random intercept, τ_r₀ = 27.9%), which together reconstitute the original 68.2%. In the growth model, the Level 2 random intercept explicitly captures those between-student baseline differences. Fig. 1, Fig. 2, and Fig. 3 captions have been correspondingly updated to reflect the four-level nesting structure. The revised Note to Table 6 also clarifies the composition of each variance component.

**3.7 Multicollinearity among the three cognitive-load predictors is unaddressed**

Table 3 shows ICL–ECL r = .45 and ECL–GCL r = −.52 — the three predictors whose "differentiated" effects are central to the paper's contribution are moderately-to-strongly correlated with one another when entered simultaneously. No VIF, tolerance, or condition-index statistics are reported, which matters particularly here given the paper's core claim rests on cleanly separating the three effects.

Response: We have added a new subsection 4.3.2 ("Multicollinearity diagnostics among the three cognitive load predictors"). Variance inflation factors computed on the group-mean-centered Level 1 predictors were: ICL VIF = 1.63, ECL VIF = 2.14, and GCL VIF = 1.72, all substantially below the conventional warning threshold of 5 and the conservative behavioral-research threshold of 4. Tolerance values ranged from 0.47 to 0.61. Condition indices computed from the standardized design matrix reached a maximum of 12.4, well below the commonly cited 30-point warning level. Thus, although the three predictors are related in the theoretically expected direction, they retain sufficient statistical independence for the multilevel model to identify their unique conditional associations with writing outcomes.

**3.8 Inter-rater reliability procedure for writing scores is underspecified**

Table 4 gives a single inter-rater r = .88 for the entire 30-item, six-dimension writing assessment. The number of raters per composition, whether scoring was blind to time point/condition, and how disagreements were resolved are not described — standard information to require for a scored open-ended writing task underlying 7,680 observations.

Response: We have added a detailed description of the writing-assessment rating procedure in Section 3.3. Each composition was scored independently by two trained raters drawn from a pool of 12 secondary-school Chinese teachers with five or more years of writing-instruction experience. Raters were blinded to student ID, class, school, wave, and prior scores through anonymized digital assignment, and assignment was randomized within each wave so that no single rater scored the same student in adjacent waves. Ratings on the six-dimension rubric were compared item by item: absolute discrepancies of one scale point or less were averaged, while discrepancies of two scale points or more were adjudicated by a senior third rater (a university-level Chinese literature instructor). Adjudication was required for 4.7% of compositions. Overall inter-rater agreement was Pearson r = 0.88, ICC(2,k) = 0.91, and Cohen's weighted kappa = 0.79; per-dimension ICC(2,k) ranged from 0.85 (creative expression) to 0.93 (mechanics). Prior to each measurement wave, all raters completed a six-hour calibration training session on 20 anchor essays to control for rater drift.

**3.9 Discussion does not engage with counter-evidence the paper itself cites**

The Introduction cites Reynaert et al.'s finding that an engaging, high-immersion learning method paradoxically impaired memory performance despite raising motivation — directly relevant to this paper's own claim that interactive teaching buffers cognitive load's negative effects on writing — but the Discussion never returns to reconcile the two findings. The Limitations paragraph is also thin (single province, one academic year) relative to the range of concerns raised in this review, and would benefit from directly addressing measurement and design limitations rather than only generalizability.

Response: We have added a dedicated new subsection 5.2 ("Reconciling with the Reynaert et al. paradox") that offers a four-point reconciliation of our finding with Reynaert et al. First, the two interventions occupy different positions on the immersion-to-guidance dimension: Reynaert et al.'s VR condition was high in immersion but low in explicit guidance, whereas our interactive teaching is moderate in immersion and high in guidance. Second, the two studies measured different downstream outcomes: memory retention (encoding-phase resource-availability dependent) versus writing (later-stage generative task drawing on consolidated schemata). Third, jointly interpreting the two studies suggests a quadratic immersion-outcome relationship. Fourth, the reconciled account yields a testable prediction: adding explicit in-headset teacher prompts to the Reynaert et al. VR protocol should reverse the memory decrement. We have also substantially expanded Section 5.3 ("Limitations") to cover four categories of limitation: measurement, design, model, and interpretive limitations, in addition to sample generalizability.

4. Minor and Presentational Concerns

**MINOR** **Grade/location stratification description**

Section 3.2 stratifies schools into three tiers by "teaching quality, geographical location and school size" (4 schools/tier), but Table 1/Fig. 4 report a different tripartite breakdown (urban 33.3% / suburban 25% / rural 41.7%) without stating how the two stratification schemes relate. Please clarify.

Response: We have clarified the sampling procedure in Section 3.2. Schools were classified into three composite tiers using an index that jointly weighted teaching quality (from the provincial department of education's annual assessment), geographic location, and school size. Within each tier, the four sampled schools were geographically distributed to include urban, suburban, and rural schools according to the population distribution of primary schools in the province, so that the composite quality-based tier stratification remains orthogonal to the geographic location descriptor that later enters the four-level model as a school-level covariate. The resulting aggregate distribution across the 12 schools was 4 urban, 3 suburban, and 5 rural schools, which when weighted by school size (average grade cohort of 160, 120, and 200 students per school in the three categories) reproduces the student-level distribution reported in Table 1 (33.3% urban, 25.0% suburban, 41.7% rural). The full cross-tabulation is provided in Supplemental Table S1.

**MINOR Grade-specific growth-rate percentages vs. the quadratic model**

The 12.3%/8.7%/6.2%/4.8% grade-3 "growth rates" reported after Table 2 are not clearly derived from the quadratic growth-curve parameters reported in Section 4.3 (π_0_, π_1_, π_2_); please specify the formula used, or move this to a supplementary calculation.

Response: We have added a clarifying note to Fig. 6. The percentage growth rates reported after Table 2 are period-to-period relative increments computed on the Grade 3 subgroup means as Delta_t = (Y-bar_(t+1) - Y-bar_t) / Y-bar_t times 100%. These descriptive percentages summarize wave-to-wave change on the observed metric and are complementary to, rather than derived from, the parametric estimates pi_1 and pi_2 of the quadratic growth curve reported in Section 4.3, which characterize the underlying velocity (linear slope) and deceleration (quadratic curvature) of the modelled trajectory.

**MINOR Copyediting and machine-translation artifacts**

Inconsistent capitalization of author names in-text ("sweller et al.," "nurullizam," "schatschneider"), inconsistent terminology ("quadratic" vs. "secondary" growth pattern used interchangeably), and several awkward constructions throughout suggest the manuscript has not had a final proofreading pass. A full language edit is needed before publication.

Response: We have carefully proofread the manuscript to correct inconsistent author-name capitalizations (Sweller, Nurullizam, Schatschneider, Cer, and others are now capitalized consistently throughout the Introduction). Terminology has been standardized: "quadratic" is now used consistently for the second-order growth term, and other machine-translation artifacts have been revised. Prior to formal publication, the entire manuscript will additionally be sent for a professional language edit by a native English editorial service, and the corresponding Language Editing Certificate will be provided to the editorial office.

**MINOR IRB approval number absent**

Even once the Ethics Statement contradiction (Sec. 2.1) is resolved, the manuscript text itself should state the approval/reference number, not just the committee name.

Response: We have provided more detailed ethical approval information in the sections '3.2 Research participants and sampling' and 'Ethics Declaration', including the ethical approval number and date.

**MINOR Effect-size benchmarking**

Multiple standardized path coefficients (~0.3–0.5) are described as "medium and above effect quantity"; please cite the specific benchmark used for interpreting HLM path coefficients (Cohen's conventions were developed for correlations/regression R^2^, and their direct application to multilevel path coefficients should be justified or softened).

Response: We have added an explicit citation for the effect-size benchmarks used to interpret the multilevel path coefficients. Following Preacher and Kelley (2011, reference [29]), the thresholds used are |beta| < 0.10 small, 0.10 <= |beta| < 0.30 medium, |beta| >= 0.30 large. These thresholds are more stringent than the classic Cohen (1988) ranges for zero-order correlations, reflecting the reduction in effect size typically expected when adjusting for the multilevel dependence structure. The clarification appears as a Note appended to Table 8, and effect-size language throughout Section 4 uses these adjusted benchmarks.

**MINOR Keyword inconsistency**

The keyword list differs between the submission metadata ("...Longitudinal study") and the in-manuscript abstract ("...Data science applications in education"); please reconcile.

Response: We have reconciled the two keyword lists by unifying them into a single six-keyword list: Classical Chinese poetry learning; Cognitive load theory; Writing ability development; Hierarchical linear modeling; Longitudinal study; Data science applications in education. The unified list now appears in the manuscript abstract and will be entered identically in the submission metadata.

**5. Strengths Worth Preserving**

● A genuinely large, well-powered nested sample (1,920 students, 48 classes, 12 schools) with four measurement waves — a scale that is uncommon in classical-poetry-education research and, if the data hold up under the checks above, would be a valuable contribution.

Response: We are grateful for the reviewer's acknowledgement of the scale and design of the study. In the revised manuscript we have preserved the full multistage stratified random sampling framework (Section 3.2) and the four-wave longitudinal design, and have made the raw and item-level data for all 1,920 students by 4 waves openly available in the project GitHub repository so that the reviewer's checks can be fully replicated (see revised Data Availability).

● Appropriate use of a three-level HLM framework for the genuinely nested data structure, with sensible centering strategy (CWC for Level-1, CGM for Level-2/3).

Response: We appreciate this acknowledgement. In the revised manuscript we have extended the framework to a four-level model as recommended in Comment 3.6 (measurement occasions, students, classes, schools; see Section 3.5), while preserving the centering strategy (group-mean centering for Level 1 predictors and grand-mean centering for Level 2 through Level 4 predictors). This ensures clean parameter interpretation and reduces multicollinearity across the four levels.

● The mediation and cross-level moderation analyses target a real and under-examined theoretical gap: how instructional context modulates the cognitive-load-to-writing pathway across developmental stages.

Response: We thank the reviewer for identifying the theoretical contribution of the mediation and cross-level moderation analyses. In the revised manuscript we have strengthened both by (a) fully operationalizing the three mediators (PSCT, IGVT, PERS; see Comment 3.1) and (b) demonstrating discriminant validity among the mediators, the poetry comprehension test, and the writing outcome (see Comment 3.2, new Section 4.2.3, and new Table 5). The cross-level moderation analysis retains its original interpretation while the underlying mediating pathways now have full psychometric documentation.

● Reported robustness checks (multiple imputation vs. complete case, bootstrap CIs, outlier-robust estimation) are a good practice and, once the modeling pipeline is clarified (Sec. 2.4), should be retained.

Response: We appreciate this acknowledgement, and the robustness checks are retained. As requested in Comment 2.4, we have also clarified the modeling pipeline: R version 4.3.2 with lme4 and lmerTest for REML estimation, brms with cmdstanr backend for Bayesian estimation of the moderated-mediation model, lavaan for CFA, and Python 3.11 with statsmodels and scikit-learn for the prediction pipeline. The BLUP-augmented mixed-model predictions are distinguished from the three-band classification derived post-hoc from the same regression predictions, and the modeling software and complete analysis scripts are archived in the GitHub repository.

**6. Checklist of Requested Revisions**

■ 1. Resolve the Ethics Statement contradiction; provide IRB name, approval number, and parental/guardian consent details for minor participants.

■ 2. Fully re-verify and correct every in-text citation number against the reference list (or vice versa).

■ 3. Confirm the GitHub data repository is public, stable, and contains the full minimal dataset (not only code); provide a permanent identifier (e.g., DOI via Zenodo/OSF) if possible.

■ 4. Clarify the exact algorithm(s) used to generate the "prediction model," reconcile the R^2^

/RMSE (regression) and accuracy/ROC-AUC (classification) results, and name the software/packages used throughout.

■ 5. Justify the mapping from NASA-TLX subscales to ICL/ECL/GCL, or relabel the construct as general perceived workload if a stronger justification isn't available.

■ 6. Add a brief, candid account of any non-significant or unexpected results encountered during model-building.

■ 7. Rewrite Table 8 and the surrounding discussion in instructional/curricular terms rather than ML-deployment terms.

■ 8. Fully describe the instruments (items, reliability, scoring) used to measure schema construction, imagery processing, and emotional resonance — currently undocumented despite anchoring the paper's central mediation result.

■ 9. Report discriminant validity (e.g., HTMT or inter-construct correlations) between the mediators, the poetry comprehension test, and the writing outcome to rule out item-content overlap as a driver of the mediation effect.

■ 10. Either add a cross-lagged/temporal-precedence analysis to support causal language, or revise

"predicted"/"effect" language throughout to association-level language.

■ 11. Address the plausibility of self-reported cognitive-load differentiation (ICL/ECL/GCL) in grade 3 children (~8 years old), or cite validation evidence for this age range.

■ 12. Reconcile equations (11)–(12) (ICC formulas) with the variance components actually reported in Table 5.

■ 13. Clarify whether the model is three-level or four-level given that Time/Time2 are embedded at Level 1, and state clearly what the reported 68.2% "student-level" variance does and does not include.

■ 14. Report VIF/tolerance statistics for ICL, ECL, and GCL given their moderate-to-strong pairwise correlations.

■ 15. Describe the writing-assessment rating procedure (number of raters per composition, blinding,

disagreement resolution).

■ 16. Reconcile the Discussion with the Reynaert et al. finding cited in the Introduction, and expand the Limitations section to cover measurement and design limitations, not only sample generalizability.

■ 17. Address the minor items in Section 4, and commission a full language/copyediting pass

Response: We again thank the reviewer for the detailed and constructive review, which has substantially strengthened the manuscript. All revisions have been highlighted in the revised manuscript for the reviewer's convenience, and the analysis code, data, and measurement instruments are archived in the project GitHub repository (see Data Availability) to support full reproducibility
