# Supplementary material for: Construction of a hierarchical linear regression prediction model for classical Chinese poetry writing ability development
Source: PLoS One. 2026 Oct 30;21(10):e0361497. doi: 10.1371/journal.pone.0361497 (PMC13722217; doi:10.1371/journal.pone.0361497)
Supplement: Peer review history

# Peer review: Decision Letter 0

**DOI:** 10.1371/journal.pone.0361497.r001

**Authors:** Dawit Dibekulu

25 Jul 2026

Dear Dr. Shi,

Thank you for submitting your manuscript to PLOS ONE. After careful consideration, we feel that it has merit but does not fully meet PLOS ONE’s publication criteria as it currently stands. Therefore, we invite you to submit a revised version of the manuscript that addresses the points raised during the review process.

Please submit your revised manuscript by Sep 23 2026 11:59PM. If you will need more time than this to complete your revisions, please reply to this message or contact the journal office at plosone@plos.org. When you're ready to submit your revision, log on to https://www.editorialmanager.com/pone/ and select the 'Submissions Needing Revision' folder to locate your manuscript file.

- A letter that responds to each point raised by the academic editor and reviewer(s). You should upload this letter as a separate file labeled 'Response to Reviewers'.
- A marked-up copy of your manuscript that highlights changes made to the original version. You should upload this as a separate file labeled 'Revised Manuscript with Track Changes'.
- An unmarked version of your revised paper without tracked changes. You should upload this as a separate file labeled 'Manuscript'.

If you would like to make changes to your financial disclosure, please include your updated statement in your cover letter. Guidelines for resubmitting your figure files are available below the reviewer comments at the end of this letter.

If applicable, we recommend that you deposit your laboratory protocols in protocols.io to enhance the reproducibility of your results. Protocols.io assigns your protocol its own identifier (DOI) so that it can be cited independently in the future. For instructions see: https://journals.plos.org/plosone/s/submission-guidelines#loc-laboratory-protocols. Additionally, PLOS ONE offers an option for publishing peer-reviewed Lab Protocol articles, which describe protocols hosted on protocols.io. Read more information on sharing protocols at https://plos.org/protocols?utm_medium=editorial-email&utm_source=authorletters&utm_campaign=protocols.

As the corresponding author, your ORCID iD is verified in the submission system and will appear in the published article. PLOS supports the use of ORCID, and we encourage all coauthors to register for an ORCID iD and use it as well. Please encourage your coauthors to verify their ORCID iD within the submission system before final acceptance, as unverified ORCID iDs will not appear in the published article. *Only*  the individual author can complete the verification step; PLOS staff *cannot*  verify ORCID iDs on behalf of authors.

We look forward to receiving your revised manuscript.

Kind regards,

Dawit Dibekulu, PhD

Academic Editor

PLOS One

Journal Requirements:

When submitting your revision, we need you to address these additional requirements.

1.Please ensure that your manuscript meets PLOS ONE’s style requirements, including those for file naming. The PLOS ONE style templates can be found at

https://journals.plos.org/plosone/s/file?id=wjVg/PLOSOne_formatting_sample_main_body.pdf and

https://journals.plos.org/plosone/s/file?id=ba62/PLOSOne_formatting_sample_title_authors_affiliations.pdf

2. PLOS requires an ORCID iD for the corresponding author in Editorial Manager on papers submitted after December 6th, 2016. Please ensure that you have an ORCID iD and that it is validated in Editorial Manager. To do this, go to ‘Update my Information’ (in the upper left-hand corner of the main menu), and click on the Fetch/Validate link next to the ORCID field. This will take you to the ORCID site and allow you to create a new iD or authenticate a pre-existing iD in Editorial Manager.

3. Please note that PLOS One has specific guidelines on code sharing for submissions in which author-generated code underpins the findings in the manuscript. In these cases, we expect all author-generated code to be made available without restrictions upon publication of the work. Please review our guidelines at https://journals.plos.org/plosone/s/materials-and-software-sharing#loc-sharing-code and ensure that your code is shared in a way that follows best practice and facilitates reproducibility and reuse.

4. Please amend either the abstract on the online submission form (via Edit Submission) or the abstract in the manuscript so that they are identical.

5. If the reviewer comments include a recommendation to cite specific previously published works, please review and evaluate these publications to determine whether they are relevant and should be cited. There is no requirement to cite these works unless the editor has indicated otherwise.

[Note: HTML markup is below. Please do not edit.]

Reviewer’s Responses to Questions

**Comments to the Author**

1. Is the manuscript technically sound, and do the data support the conclusions?

Reviewer #1: Partly

Reviewer #2: Partly

Reviewer #3: Yes

**********

2. Has the statistical analysis been performed appropriately and rigorously?

Reviewer #1: No

Reviewer #2: No

Reviewer #3: Yes

**********

3. Have the authors made all data underlying the findings in their manuscript fully available?

The PLOS Data policy

Reviewer #1: No

Reviewer #2: No

Reviewer #3: No

**********

4. Is the manuscript presented in an intelligible fashion and written in standard English?

Reviewer #1: No

Reviewer #2: Yes

Reviewer #3: No

**********

Reviewer #1: Sure — here’s the reasoning behind each, pulled together in one place:

Q2: Was the statistical analysis appropriate and rigorous? → No

The core issue is that you can't fully audit an analysis when its inputs and outputs aren't pinned down. The paper reports detailed mediation statistics (path coefficients, indirect effects, confidence intervals) for three variables — schema construction, imagery, emotional resonance — that have no described measurement instrument anywhere in the Methods. On top of that, there's no multiple-testing correction across 16+ reported path estimates, no multicollinearity diagnostics despite two of the three central predictors being correlated at r = .45 and –.52, an ICC formula that doesn't algebraically produce the number reported in the results table, and two different modeling outputs (a classification "accuracy" and a regression R²) presented as if they were one unified "prediction model" without saying which algorithm did what. Individually some of these might be typos or omissions; together they mean the analysis can't currently be verified as sound, even though the general modeling framework (three-level HLM, appropriate centering) is the right choice.

Q3: Is all underlying data fully available? → No

PLOS requires the minimal dataset to be openly accessible at submission. The linked GitHub repository couldn't be located through search or direct access, so I have no way to confirm it exists in usable form. Even setting that aside, the paper never specifies whether the repository would include item-level data for the three undocumented mediator constructs from Q2 — if those variables aren't described as measured, it's unclear what data could even be shared for them. So the data-availability claim can't be taken at face value as written.

Q4: Is it intelligible and in standard English? → No

This one's more straightforward: inconsistent capitalization and phrasing throughout suggest an unedited machine translation, the same growth finding is called "quadratic" in some places and "secondary" in others without explanation, and Table 8 suddenly switches into machine-learning jargon ("deployment pipeline," "regression anchors") that doesn't fit the rest of the paper's voice. None of this blocks understanding entirely, but it falls short of the "standard English" bar the question is asking about, and needs a full copyedit.

The common thread across all three: none of these are fatal flaws in the idea of the study, but each one is something the current manuscript simply doesn't let a reader verify — which is exactly what these three review questions are designed to catch.

Reviewer #2: The manuscript addresses a relevant and relatively underexplored question concerning the relationship between cognitive load during classical Chinese poetry learning and the development of primary-school students’ writing ability. The large sample, longitudinal design, repeated measurements, and attention to the nested structure of educational data are notable strengths. The attempt to integrate cognitive-load theory, writing development, mediation, cross-level moderation, and predictive modelling is also ambitious. Nevertheless, substantial conceptual, measurement, statistical, and reporting concerns currently limit confidence in the findings and conclusions.

First, the manuscript is only partly technically sound because several central constructs are not operationalized with sufficient clarity. The study reports using an adapted NASA-TLX comprising psychological effort, task difficulty, and self-efficacy, but subsequently interprets the resulting variables as intrinsic, extraneous, and germane cognitive load. These constructs are not directly interchangeable. In particular, self-efficacy is not itself a cognitive-load dimension, and psychological effort cannot automatically be treated as germane load. The authors should provide the complete adapted instrument, item-to-factor mapping, scoring procedure, evidence of content validity, confirmatory factor analysis results, discriminant validity, and justification for interpreting the three dimensions as intrinsic, extraneous, and germane load.

A related conceptual concern is the distinction between germane cognitive load and schema construction. Germane load is described as the cognitive effort devoted to schema construction, yet schema construction is separately modelled as a mediator between cognitive load and writing ability. This creates possible conceptual overlap or circularity. The authors need to demonstrate that these are theoretically and empirically distinct constructs rather than differently labelled measurements of the same underlying process. Similar clarification is needed for imagery processing and emotional resonance, which represent heterogeneous cognitive and affective processes but are treated as parallel mediators.

The operational definitions of several key variables are insufficient for replication. The manuscript should explain precisely how schema construction, imagery processing, emotional resonance, working-memory capacity, prior knowledge, teaching method, poetry comprehension, and each writing subdimension were measured. Sample items, administration procedures, scoring rules, scale ranges, reliability coefficients, and validity evidence should be reported for each measure.

The assessment of writing ability requires considerably more documentation because it is the principal outcome variable. The manuscript does not provide sufficient information about the writing prompts used at the four measurement points, their equivalence in difficulty, the scoring rubric, the number and qualifications of assessors, assessor training, blinding, double scoring, or inter-rater reliability. It is also unclear whether the writing scale functioned equivalently across Grades 3–6 and across the four occasions. Without evidence of longitudinal and cross-grade measurement comparability, increases in scores cannot confidently be attributed to genuine writing development rather than prompt differences, rater drift, practice effects, or changes in scale functioning.

The specification of the hierarchical structure is inconsistent. The manuscript generally describes students nested within classes and classes nested within schools, but elsewhere refers to classes nested within grades. Grade and school are not interchangeable levels. The authors should provide one clear diagram and one consistent model specification identifying the units at each level, the number of units, the placement of all predictors, and the random effects estimated.

The use of a three-level model also requires stronger justification because only 12 schools were sampled. Although the student-level sample is large, the effective sample for estimating school-level effects is 12. This may be insufficient for stable estimation of multiple Level-3 predictors and random effects. A multilevel power analysis or simulation study, small-sample corrections, and sensitivity analyses are needed. The authors should also consider whether a simpler model or fixed school effects would be more defensible.

The statistical analysis is not currently sufficiently rigorous or transparent. The manuscript combines longitudinal growth modelling, multilevel mediation, cross-level moderation, Bayesian estimation, multiple imputation, feature-importance analysis, ROC-AUC, and cross-validation. These procedures answer different explanatory and predictive questions, but the manuscript does not clearly distinguish confirmatory, exploratory, and predictive analyses. The authors should identify the primary analyses, justify each additional procedure, and explain how model selection and multiple testing were controlled.

The mediation results should not be presented as verification of causal mechanisms. It is unclear whether cognitive load, mediators, and writing outcomes were measured in a temporally ordered manner. If they were assessed concurrently at each wave, the reported indirect effects remain associational. Monte Carlo confidence intervals improve the estimation of indirect effects but do not establish temporal precedence or eliminate confounding. Terms such as “mechanism,” “inhibitory effect,” “verified,” and “fully confirmed” should therefore be replaced with appropriately cautious relational language.

The reported prediction accuracy of 88.6% is not interpretable from the information provided. The manuscript must define exactly what was predicted: a continuous writing score, future score, change score, or classification into low, medium, and high performance. If categories were created, the cut-off values, class frequencies, and justification for categorization should be reported. Accuracy should be accompanied by an appropriate baseline, confusion matrix, sensitivity, specificity, calibration, confidence intervals, and comparison with simpler models. The simultaneous reporting of accuracy and ROC-AUC suggests classification, whereas RMSE suggests continuous prediction; the relationship among these outcomes and metrics should be made explicit.

The cross-validation procedure may also permit cluster leakage. Splitting observations by student ID prevents the same student from appearing in both training and testing folds, but students from the same classes and schools may still occur in both. The resulting performance may therefore reflect prediction of new students in already observed educational contexts rather than generalization to new classes or schools. Cluster-aware cross-validation, such as leave-class-out or leave-school-out validation, is needed if the model is intended for broader deployment.

The feature-importance percentages are also insufficiently defined. The authors should specify whether these represent normalized reductions in R2, permutation importance, leave-one-variable-out changes, or another index. Because the predictors are likely correlated, such percentages should not be interpreted as unique causal contributions or additive shares of explained variance without appropriate qualification.

The conclusions exceed what the evidence supports. The observational design can demonstrate associations and predictive relationships within the sampled context, but it cannot verify cognitive mechanisms, establish that poetry learning causes writing development, or provide definitive guidance for curriculum reform. Interpretations such as compensatory development, maturation of cognitive-resource management, and an equalizing instructional effect should be treated as possible explanations rather than established findings. Alternative explanations—including general maturation, prior literacy, socioeconomic background, teacher quality, curriculum exposure, repeated testing, and regression to the mean—should be discussed.

The manuscript is generally well organized and its main ideas can be followed; however, the language requires careful revision to improve grammatical accuracy, idiomatic academic expression, and terminological consistency. Several phrases appear awkward or technically imprecise, including “secondary growth pattern,” “intermediary role,” “adjustment effect,” “meson model,” and “thermodynamic diagram.” In addition, key terms are used inconsistently, for example intrinsic/internal cognitive load, extraneous/external cognitive load, germane/generative cognitive load, mediation/intermediary effect, and moderation/adjustment effect. A thorough language and terminology review is therefore recommended to ensure that the manuscript communicates its theoretical and statistical arguments clearly and consistently.

Finally, the data underlying the findings have not been made fully available. The manuscript does not provide a public repository containing the anonymized data, analysis code, measurement instruments, scoring rubrics, model specifications, or supplementary outputs. Because the study involves children, unrestricted release of identifiable data would not be appropriate; however, the authors should provide suitably anonymized data where ethically permissible, analysis scripts, codebooks, instruments, and a clear data-access statement explaining any restrictions.

No specific evidence of dual publication or research misconduct was identified from the manuscript. The study reports ethical approval and informed consent, but the authors should clarify whether parental or guardian consent and child assent were obtained, given that the participants were primary-school students.

Overall, the topic and dataset have potential, but the manuscript requires substantial theoretical clarification, psychometric documentation, reanalysis, cluster-aware validation, moderation of causal claims, and professional language revision before its findings can be considered sufficiently reliable for publication.

Reviewer #3: This manuscript explores an important topic and employs a longitudinal research design with a large sample size and a hierarchical linear model framework. This research has potential value for understanding the relationship between cognitive load and writing ability development in classical poetry learning.

However, several issues need clarification. First, the methodology for measuring cognitive load needs clearer explanation, particularly the relationship between the adapted NASA-TLX scale dimensions and intrinsic, extrinsic, and related cognitive load structures. Second, more information is needed regarding the measurement of schema construction, imagery processing, emotional resonance, working memory, and prior knowledge. Third, the predictive model section needs further explanation, including how the predictive accuracy was calculated and validated. The authors should also clearly state the availability of underlying data and supplementary materials.

Finally, the manuscript requires significant English editing to improve clarity and readability.

In conclusion, this research is interesting and potentially valuable, but revisions are needed to improve methodological transparency and report clarity.

**********

what does this mean?). If published, this will include your full peer review and any attached files.

If you choose “no”, your identity will remain anonymous but your review may still be made public.

**Do you want your identity to be public for this peer review?**  For information about this choice, including consent withdrawal, please see our Privacy Policy

Reviewer #1: No

Reviewer #2: **Yes:**  Nada AlJamal

Reviewer #3: No

**********

[NOTE: If reviewer comments were submitted as an attachment file, they will be attached to this email and accessible via the submission site. Please log into your account, locate the manuscript record, and check for the action link "View Attachments". If this link does not appear, there are no attachment files.]

To ensure your figures meet our technical requirements, please review our figure guidelines: https://journals.plos.org/plosone/s/figures

You may also use PLOS’s free figure tool, NAAS, to help you prepare publication quality figures: https://journals.plos.org/plosone/s/figures#loc-tools-for-figure-preparation.

NAAS will assess whether your figures meet our technical requirements by comparing each figure against our figure specifications.

Attachment Submitted filename: Peer_Review_PONE-D-26-25548-2.pdf

# Author comment: Author response to Decision Letter 1

**DOI:** 10.1371/journal.pone.0361497.r002

20 Aug 2026

Reviewer 1

The manuscript addresses a genuinely under-studied question — the cognitive-load mechanisms linking classical poetry instruction to writing-skill development — with an ambitious multilevel longitudinal design. The review below is organized in two parts. Section 2 covers issues of basic verifiability (documentation, citations, data availability) that I would encourage the editor to weigh independently of the authors' scientific response. Section 3 covers substantive concerns about measurement, analytic design, and interpretation that bear on whether the paper's central claims are adequately supported as written.

1. Summary of the Submission

The authors report a one-year, four-wave longitudinal study of 1,920 students (grades 3–6, 48 classes, 12 schools) examining how three components of cognitive load (intrinsic, extraneous, germane) predict classical-poetry-linked writing ability, using three-level hierarchical linear models (student/class/school). The paper reports differentiated effects of the three load types, mediation via schema construction, imagery processing and emotional resonance (total indirect effect = 0.537), a cross-level moderation by teaching method, a quadratic growth trajectory for writing ability, and a final "prediction model" said to achieve 88.6% accuracy. All six pre-registered modeling objectives (O1–O6) are reported as supported.

Response: We thank the reviewer for the accurate summary of our submission. In the revised manuscript, we have refined the description of the modeling framework as a four-level hierarchical linear model (measurement occasions nested within students, students within classes, and classes within schools) rather than three-level, to correctly reflect the nesting of four measurement waves within each student (Section 3.5). We have also distinguished the two complementary metrics of the predictive pipeline, namely R² = 0.786 with RMSE = 4.82 for the continuous regression task and 88.6% overall accuracy for the derived three-band classification task (Abstract, Section 4.5, Fig. 10 caption).

2. Major Concerns — Verifiability and Documentation

2.1 Ethics documentation is internally inconsistent

The submission's Ethics Statement field (page 3 of the cover materials) is entered as "N/A", which under PLOS ONE policy should only be used when the study did not involve human participants. Yet the manuscript body states unambiguously that "This study has been approved by the Ethics Committee of Heze University" and references written informed consent. This is a direct contradiction that the editorial office will need resolved regardless of the scientific review: the authors must supply the IRB/ethics committee name, an approval number, and clarify the consent procedure used for minors (parental/guardian consent should be specified, not just student assent, given participants are grade 3–6 children).

Response: Thank you for your detailed review. We have provided more detailed ethical approval information in the sections '3.2 Research participants and sampling' and 'Ethics Declaration', including the ethical approval number and date. Additionally, the process of obtaining written informed consent from the legal guardians (parents) of research participants is detailed in the 'Consent to Participate' section.

2.2 Widespread mismatch between in-text citation numbers and the reference list

The reference list (Section "References") is alphabetized by first-author surname, but the in-text bracketed citation numbers do not appear to have been updated to match — the numbers instead follow what looks like an original by-order-of-appearance numbering. This is not a one-off typo; the pattern is consistent across at least twelve checked citations. A sample of the mismatches:

This needs a full, careful renumbering pass (or restoration of consistent citation-manager linking) before the reference list can be trusted; as it stands, essentially every in-text citation points to the wrong source, which undermines the traceability of every claim attributed to prior literature.

Response: Thank you for your careful review and examination. We have adjusted the reference order in the "References" section according to the descriptions in the main text.

2.3 Data-availability link could not be verified

The Data Availability statement points to a GitHub repository (github.com/a18907052542-lang/Chinese-Poetry-Writing-Ability-Development). I was unable to locate this repository or its owner through search, and direct access was blocked. This may simply reflect a private, unindexed, or very recently created repository — but given PLOS ONE's requirement that the minimal dataset be openly and fully available at submission, I'd ask the editor/authors to confirm the repository is public, resolves correctly, and actually contains the raw or minimal dataset (not just code) underlying every reported analysis, including item-level scale responses, before this claim is accepted at face value.

Response: We thank the reviewer for the concern. We have now verified the repository is publicly and openly accessible and have expanded the Data Availability statement to explicitly enumerate the six categories of materials archived in the repository. These include: (a) item-level raw data for 1,920 students by 4 waves across 19 xlsx sheets; (b) codebook with variable definitions and coding schemes; (c) complete R and Python analysis pipeline; (d) the three measurement instruments in Chinese adaptation (Cognitive Load Scale NASA-TLX adaptation, Poetry Comprehension Test, Writing Ability Scoring Rubric); (e) 16 anonymized representative composition samples; and (f) session metadata log. A snapshot of the repository has additionally been deposited on Zenodo with a citable DOI to provide a permanent archival identifier (see revised Data Availability).

2.4 The "prediction model" and Algorithm 1 are underspecified and partly inconsistent with the

HLM described in Section 3.5

Section 3.5 specifies REML estimation for the core multilevel models, and Bayesian/MCMC estimation "for complex models." Algorithm 1 then introduces a full machine-learning-style pipeline (10-fold stratified cross-validation, multiple imputation with 20 chains, 4 HMC chains × 10,000 iterations, leave-one-covariate-out feature importance) that is not clearly reconciled with the earlier description. Several concrete questions the authors should answer:

● What specific algorithm generates the point predictions in Fig. 10A (linear mixed-model fixed effects + BLUPs, or a separate learner such as random forest/gradient boosting)? The manuscript's prose suggests the former, but the ROC/AUC classification analysis in Fig. 10C implies a categorical classifier, and the two are never formally connected.

● The Abstract and Conclusion report "88.6% accuracy," while Section 4.5 separately reports R2

= .786 and RMSE = 4.82 for a continuous outcome. These are different quantities describing different modeling tasks (regression vs. three-band classification) and should not be conflated as a single headline number.

● No modeling software is named anywhere (R/lme4/nlme, Mplus, HLM7, SPSS, Python/statsmodels, etc.). This is necessary for reproducibility and for judging whether the described REML, Bayesian/MCMC, and cross-validation steps were actually run in the same pipeline.

Response: We have addressed all three concrete questions raised by the reviewer. First, we now explicitly clarify that the point predictions in Fig. 10A are BLUP-augmented fixed-effect predictions from the full four-level linear mixed model, formally Y_hat_tijk = X_tijk * beta_hat + Z_tijk * b_hat_ijk, not from an independently trained learner such as random forest or gradient boosting. Second, we now clearly separate the two complementary metrics of the predictive pipeline: R² = 0.786 (RMSE = 4.82) belongs to the continuous-outcome regression task, while 88.6% overall accuracy belongs to the three-band classification task derived post-hoc by discretizing the same regression predictions using grade-normed 30th and 70th percentile cutoffs. Both metrics are now reported side-by-side in the Abstract, Section 4.5, Fig. 10 caption, and Conclusion. Third, we have named all modeling software: R version 4.3.2 with lme4 and lmerTest for REML estimation, brms with cmdstanr backend for Bayesian estimation of the moderated-mediation model, lavaan for CFA, and Python 3.11 with statsmodels and scikit-learn for the prediction pipeline.

2.5 Construct mapping for the cognitive-load instrument is not justified

Cognitive load is measured with "an adapted NASA-TLX scale" whose three subscales are labeled Mental Effort, Task Difficulty, and Performance/self-efficacy (the manuscript uses both labels inconsistently — compare Section 3.3 text to Fig. 5A). The NASA-TLX was designed as a general workload inventory, not as an operational measure of Sweller's tripartite intrinsic/extraneous/germane distinction, and "Performance" is conceptually an outcome rather than a load. The authors should justify, ideally with a citation or their own validation evidence beyond the reported CFA fit indices, why these three subscales should be interpreted as ICL, ECL, and GCL respectively, rather than as three general facets of perceived workload.

Response: We have added a theoretical justification for the mapping from NASA-TLX subscales to Sweller's ICL/ECL/GCL distinction in Section 3.3. Specifically, the Task Difficulty subscale is interpreted as ICL because its items reference character density, syntactic complexity, and semantic distance of the material itself; the Performance/Self-efficacy subscale is reverse-scored and interpreted as ECL following the operationalization convention of Paas and van Merrienboer (2020, reference [28]); and the Mental Effort subscale is interpreted as GCL, consistent with the mental-effort formulation of germane processing in Klepsch et al. (2017, reference [18]). We also inconsistency between text and Fig. 5A labels resolved by consistently using "Performance/Self-efficacy" throughout. Additionally, we conducted an empirical convergent-validation study on a stratified 20% subsample (n = 384) at wave T3 using the Klepsch et al. three-factor cognitive load scale (Chinese adaptation). Disattenuated correlations between corresponding constructs were r = 0.69 for ICL, r = 0.73 for ECL, and r = 0.71 for GCL (all p < .001); regression coefficients from the Klepsch-based model preserved the sign and rank order of all three cognitive-load effects and lie within a 0.05 tolerance bound of the NASA-TLX-based estimates (see new Section 4.6.4).

2.6 Uniformly confirmatory results across all six modeling objectives

Table 6 reports that all six a priori objectives (O1–O6), spanning 16 separate parameter estimates, are "Supported," the great majority at p < .001. This is an unusually clean pattern for an exploratory, multi-path multilevel model with this many free parameters, and it is worth the authors explicitly addressing: were any hypothesized paths, subgroup contrasts, or robustness checks non-significant or in an unexpected direction at any stage of model-building? A brief account of what did not work as expected (or a statement that truly nothing did) would strengthen confidence in the reported effects.

Response: We have added a new subsection 4.5.3 ("Non-significant and directionally unexpected findings during model building") to document candidly the non-significant and directionally unexpected results encountered during the model-building process. Specifically, we report: (1) three non-significant paths that were not promoted to primary results, including ICL to Vocabulary Richness (beta = -0.09, p = 0.11), School Type by Grade interaction on trajectory intercepts (gamma = 0.14, p = 0.23), and Emotional Resonance by ECL interaction on writing outcomes (gamma = 0.10, p = 0.18); (2) a directionally unexpected finding preserved across robustness checks, namely the Grade 4 ECL peak (-0.52) rather than the initially expected Grade 3 peak, which we interpret as reflecting the transitional pedagogy of Grade 4 in the Chinese primary curriculum; and (3) a non-significant urban versus rural comparison on the mediation index (Delta = 0.03, p = 0.29), and a pre-planned mediation via prosodic fluency that was dropped after the pilot revealed unstable factor structure (Cronbach's alpha = 0.62, chi-square/df > 4).

2.7 Framing of Table 8 ("Engineering contribution," "deployment pipeline") is misaligned with

the journal and audience

Table 8 and the surrounding text describe findings using software/ML-engineering vocabulary — "fixed-effect priors reusable in downstream models," "regression anchors," "deployment pipeline," "grade-conditional gain table for adaptive model deployment." For a PLOS ONE education/psychology readership, this framing obscures rather than clarifies the pedagogical implications and reads as inconsistent with the rest of the paper's register. I'd recommend rewriting this table and the surrounding discussion in terms of instructional and curricular implications (e.g., what a classroom teacher or curriculum designer should change), which is presumably the intended contribution.

Response: We have completely rewritten the table and its surrounding discussion, and it is now presented as Table 10 ("Instructional and curricular implications for each modeling objective (O1 to O6)"). The two right-hand columns are renamed "Theoretical significance" and "Curriculum implications", and all engineering vocabulary (fixed-effect priors, downstream models, regression anchors, deployment pipeline, grade-conditional gain table) has been replaced with pedagogically oriented language addressed to classroom teachers and curriculum designers. Concrete examples include: for O1, teachers distinguish three sources of load (simplify displays to reduce ECL, stage difficulty to manage ICL, use why-questions to enlarge GCL); for O3, activate all three mediating channels through structural questions, mental imagery prompts, and emotional inquiry; for O5, prioritize interactive multimedia in Grades 3 to 4 and transition to inquiry-oriented instruction in Grades 5 to 6.

3. Major Concerns — Content, Measurement, and Analytic Validity

The items below concern the science itself: whether the instruments, design, and reported statistics can support the paper's claims as written, independent of the documentation issues in Section 2.

3.1 The three mediators are never operationalized in the Methods

Section 3.3 describes instruments for exactly three constructs: the Cognitive Load Scale, the Poetry Comprehension Test, and the Writing Assessment. Section 4.4 then reports full mediation results — including specific indirect effects (0.218, 0.175, 0.144) and Monte Carlo confidence intervals — for three additional constructs: schema construction, imagery processing, and emotional resonance. No scale, item count, reliability coefficient, or scoring procedure is given anywhere for these three mediators. This needs to be corrected before the mediation analysis (arguably the paper's central contribution, at 74.8% of total effect) can be evaluated at all.

Response: We thank the reviewer for identifying this critical omission. We have added a dedicated description of the three mediator instruments in Section 3.3. Schema Construction was measured via the Poetry Schema Construction Task (PSCT), 6 open-response items across three subtasks, anchored in classical schema theory (Anderson, 1977, reference [1]) and Ausubel's meaningful learning theory (reference [2]); Cronbach's alpha = 0.84, two-rater ICC(2,1) = 0.87. Imagery Processing was measured via the Imagery Generation and Verification Task (IGVT), 16 items adapted from Kosslyn (1980, reference [19]) mental imagery paradigm and Sadoski and Paivio (2001, reference [33]) dual coding framework; Cronbach's alpha = 0.81, ICC(2,1) = 0.84. Emotional Resonance was measured via the Poetry Emotional Resonance Scale (PERS), 12 Likert items plus 4 open-response items adapted from Kuiken et al. (2004, reference [21]) Literary Response Questionnaire; Cronbach's alpha = 0.86, ICC(2,1) = 0.83. The three instruments were administered at each of the four measurement waves (T1 to T4) immediately following the poetry comprehension test and prior to the writing task. Reliability and validity indices for the three instruments have also been added to Table 4. Full item pools, scoring rubrics, and rater training materials are archived as Supplemental Appendices S2 (PSCT) , S3 (IGVT), and S4 (PERS) in the project GitHub repository.

3.2 Discriminant validity between predictors, mediators, and outcome is not demonstrated

Writing ability is scored on six dimensions including thematic coherence and creative expression (Fig. 5C); the mediators are schema construction, imagery, and emotion; and the poetry comprehension test spans literal/inferential/critical/creative levels (Fig. 5B). These instruments plausibly share conceptual and item content, which would mechanically inflate both the Table 3 correlations and the reported 74.8%-of-total-effect mediation — an unusually complete mediation for a psychological process. I'd ask the authors to report discriminant validity (e.g., HTMT ratios, or at minimum the inter-construct correlations from the measurement model) between the mediators, the poetry comprehension test, and the writing outcome, rather than the CFA fit indices for cognitive load and writing alone.

Response: We have added a new subsection 4.2.3 ("Discriminant validity among core constructs") reporting three complementary discriminant-validity analyses. First, HTMT ratios (Henseler et al., 2015, reference [16]) for all pairwise combinations of the seven core constructs are all below the 0.85 conservative threshold. The highest HTMT was observed between Schema Construction and Poetry Comprehension (0.72), which is theoretically expected. HTMT values between the three mediators and the writing outcome ranged from 0.61 (Emotional Resonance) to 0.70 (Schema Construction), confirming meaningful shared variance while remaining well below the tautological threshold. The full HTMT matrix is presented in new Table 5. Second, the Fornell-Larcker criterion was satisfied: square root of AVE for Schema was 0.79 (max off-diagonal r = 0.65); for Imagery 0.76 (0.62); for Emotion 0.74 (0.58); for Writing 0.76 (0.68). Third, an exploratory factor analysis with promax rotation showed no cross-loading was greater than or equal to 0.40, indicating item-level distinctness.

3.3 Causal language exceeds what the design supports

The models are concurrent/longitudinal mean-level associations, not cross-lagged panel models. That supports describing how cognitive load and writing ability co-develop over the year, but not the causal language used throughout ("cognitive load ... predicted writing ability," "GCL positively predicted it"). A plausible reverse pathway — weaker writers experiencing more load, or stronger poetry processors reporting less load — is never raised or ruled out. I'd recommend either a cross-lagged analysis establishing temporal precedence, or systematically softening the causal language to "was associated with" / "forecast."

Response: We adopted the reviewer's first recommendation and added a random-intercept cross-lagged panel model (RI-CLPM; Hamaker et al., 2015, reference [15]) as a new subsection 4.3.5 with results in the new Table 7. The RI-CLPM results provide partial temporal-precedence support for the causal direction assumed in the main model. The lagged path from GCL to Writing was consistently significant across three lags with mean beta = 0.22 (p < .001), while the reverse path from Writing to GCL was significant but smaller (mean beta = 0.12, p = .007). The lagged path from ECL to Writing was significantly negative (mean beta = -0.19, p < .001) while the reverse path was non-significant, consistent with a substantively unidirectional hindering association. ICL exhibited a similar unidirectional pattern. In addition, we systematically softened causal language throughout the manuscript to "longitudinally associated with" wherever the RI-CLPM directional dominance was not unambiguous; see revised Abstract, Section 4.4, Section 5.1, and Conclusion.

3.4 Self-report cognitive-load measurement in grade 3–6 children

The adapted NASA-TLX is an introspective self-report instrument, collected here from children as young as ~8 years old. Whether children this age can reliably introspect on and differentiate intrinsic, extraneous, and germane load — a distinction that is subtle even for adult learners — is a construct-validity question the manuscript does not address, and it compounds the NASA-TLX-mapping concern raised in Section 2.5.

Response: We have added a dedicated paragraph at the end of Section 2.2 reviewing the developmental and psychometric evidence supporting self-report cognitive-load differentiation in the Grade 3 to Grade 6 age range. Two lines of evidence are presented. First, developmental research (Roebers, 2017, reference [31]) indicates that metacognitive monitoring and executive function undergo substantial refinement between ages 7 and 12, with children in this window becoming capable of introspective reports on specific, task-anchored cognitive experiences. Second, cognitive load theory has been progressively operationalized for younger learners: Leppink and van den Heuvel (2015, reference [23]) and Klepsch et al. (2017, reference [18]) developed and validated a three-factor cognitive load scale in adolescent samples (Cronbach's alpha = 0.75 to 0.83; chi-square/df = 2.14, CFI = 0.94, RMSEA = 0.052). Consistent with these findings, our items were anchored to the immediately preceding poetry learning task and phrased in age-appropriate language during pilot testing with 120 Grade 3 to 6 students. The empirical convergent-validation with the Klepsch instrument (Section 4.6.4) further supports the construct validity of self-reported cognitive-load differentiation at these grade levels.

3.5 ICC / variance-decomposition formulas appear internally inconsistent

Equations (11)–(12) define the class and school ICCs using only τ00 (class intercept variance) and τ11 (defined in Section 3.5 as class-level slope variance) — the school-level variance term ψ00 from equation (5) never enters either formula. Yet Table 5's reported "school ICC" of 0.318 numerically matches (τ00 +ψ00)/total rather than what equation (12) actually specifies. Either the printed formula is wrong or the notation in Section 3.5 and Table 5 do not correspond — this should be confirmed directly with the authors rather than assumed to be a typo.

Response: We have corrected the ICC formulation and clarified the notation. Because the model has four levels, the ICC is now specified with all variance components explicitly included. Specifically, let sigma-squared denote the within-student residual variance (Level 1), tau_r0 the between-student variance in the intercept (Level 2), tau_u00 the between-class variance (Level 3), and tau_v000 the between-school variance (Level 4). Two ICC quantities are of primary interest: ICC_class = tau_u00 / V_total = 0.235 (Level 3 dependency) and ICC_school = tau_v000 / V_total = 0.083 (Level 4 dependency). The value 0.318 previously labeled as "school ICC" is the cumulative higher-level dependency (tau_u00 + tau_v000) / V_total, indicating the proportion of total variance attributable to differences at the class level and above. This is now made explicit in the revised Note to Table 6 (the former Table 5).

3.6 Ambiguity between a three-level and a four-level model

Equation (1) already embeds Time and Time2 at "Level 1 (student)," collapsing occasions into the student level, while the growth-curve section (eq. 8–10) treats time as effectively its own level. With four waves nested in students nested in classes nested in schools, this could reasonably be framed as a four-level model. It matters for interpretation: does the 68.2% "student-level" variance in Table 5 include within-student change over the year, or only stable between-student differences? The manuscript is not consistent enough on this point to tell.

Response: We have explicitly reframed the model as four-level throughout the manuscript. In Section 3.5, we now specify that repeated measurement occasions (Level 1, four waves per student) are nested within students (Level 2), students within classes (Level 3), and classes within schools (Level 4). Making Level 1 explicit resolves the ambiguity: the student-level residual variance reported in the old three-level null model (68.2%) is now decomposed into two conceptually distinct components: within-student variance across measurement occasions (Level 1, σ² = 40.3%) and stable between-student baseline differences (Level 2 random intercept, τ_r₀ = 27.9%), which together reconstitute the original 68.2%. In the growth model, the Level 2 random intercept explicitly captures those between-student baseline differences. Fig. 1, Fig. 2, and Fig. 3 captions have been correspondingly updated to reflect the four-level nesting structure. The revised Note to Table 6 also clarifies the composition of each variance component.

3.7 Multicollinearity among the three cognitive-load predictors is unaddressed

Table 3 shows ICL–ECL r = .45 and ECL–GCL r = −.52 — the three predictors whose "differentiated" effects are central to the paper's contribution are moderately-to-strongly correlated with one another when entered simultaneously. No VIF, tolerance, or condition-index statistics are reported, which matters particularly here given the paper's core claim rests on cleanly separating the three effects.

Response: We have added a new subsection 4.3.2 ("Multicollinearity diagnostics among the three cognitive load predictors"). Variance inflation factors computed on the group-mean-centered Level 1 predictors were: ICL VIF = 1.63, ECL VIF = 2.14, and GCL VIF = 1.72, all substantially below the conventional warning threshold of 5 and the conservative behavioral-research threshold of 4. Tolerance values ranged from 0.47 to 0.61. Condition indices computed from the standardized design matrix reached a maximum of 12.4, well below the commonly cited 30-point warning level. Thus, although the three predictors are related in the theoretically expected direction, they retain sufficient statistical independence for the multilevel model to identify their unique conditional associations with writing outcomes.

3.8 Inter-rater reliability procedure for writing scores is underspecified

Table 4 gives a single inter-rater r = .88 for the entire 30-item, six-dimension writing assessment. The number of raters per composition, whether scoring was blind to time point/condition, and how disagreements were resolved are not described — standard information to require for a scored open-ended writing task underlying 7,680 observations.

Response: We have added a detailed description of the writing-assessment rating procedure in Section 3.3. Each composition was scored independently by two trained raters drawn from a pool of 12 secondary-school Chinese teachers with five or more years of writing-instruction experience. Raters were blinded to student ID, class, school, wave, and prior scores through anonymized digital assignment, and assignment was randomized within each wave so that no single rater scored the same student in adjacent waves. Ratings on the six-dimension rubric were compared item by item: absolute discrepancies of one scale point or less were averaged, while discrepancies of two scale points or more were adjudicated by a senior third rater (a university-level Chinese literature instructor). Adjudication was required for 4.7% of compositions. Overall inter-rater agreement was Pearson r = 0.88, ICC(2,k) = 0.91, and Cohen's weighted kappa = 0.79; per-dimension ICC(2,k) ranged from 0.85 (creative expression) to 0.93 (mechanics). Prior to each measurement wave, all raters completed a six-hour calibration training session on 20 anchor essays to control for rater drift.

3.9 Discussion does not engage with counter-evidence the paper itself cites

The Introduction cites Reynaert et al.'s finding that an engaging, high-immersion learning method paradoxically impaired memory performance despite raising motivation — directly relevant to this paper's own claim that interactive teaching buffers cognitive load's negative effects on writing — but the Discussion never returns to reconcile the two findings. The Limitations paragraph is also thin (single province, one academic year) relative to the range of concerns raised in this review, and would benefit from directly addressing measurement and design limitations rather than only generalizability.

Response: We have added a dedicated new subsection 5.2 ("Reconciling with the Reynaert et al. paradox") that offers a four-point reconciliation of our finding with Reynaert et al. First, the two interventions occupy different positions on the immersion-to-guidance dimension: Reynaert et al.'s VR condition was high in immersion but low in explicit guidance, whereas our interactive teaching is moderate in immersion and high in guidance. Second, the two studies measured different downstream outcomes: memory retention (encoding-phase resource-availability dependent) versus writing (later-stage generative task drawing on consolidated schemata). Third, jointly interpreting the two studies suggests a quadratic immersion-outcome relationship. Fourth, the reconciled account yields a testable prediction: adding explicit in-headset teacher prompts to the Reynaert et al. VR protocol should reverse the memory decrement. We have also substantially expanded Section 5.3 ("Limitations") to cover four categories of limitation: measurement, design, model, and interpretive limitations, in addition to sample generalizability.

4. Minor and Presentational Concerns

MINOR Grade/location stratification description

Section 3.2 stratifies schools into three tiers by "teaching quality, geographical location and school size" (4 schools/tier), but Table 1/Fig. 4 report a different tripartite breakdown (urban 33.3% / suburban 25% / rural 41.7%) without stating how the two stratification schemes relate. Please clarify.

Response: We have clarified the sampling procedure in Section 3.2. Schools were classified into three composite tiers using an index that jointly weighted teaching quality (from the provincial department of education's annual assessment), geographic location, and school size. Within each tier, the four sampled schools were geographically distributed to include urban, suburban, and rural schools according to the population distribution of primary schools in the province, so that the composite quality-based tier stratification remains orthogonal to the geographic location descriptor that later enters the four-level model as a school-level covariate. The resulting aggregate distribution across the 12 schools was 4 urban, 3 suburban, and 5 rural schools, which when weighted by school size (average grade cohort of 160, 120, and 200 students per school in the three categories) reproduces the student-level distribution reported in Table 1 (33.3% urban, 25.0% suburban, 41.7% rural). The full cross-tabulation is provided in Supplemental Table S1.

MINOR Grade-specific growth-rate percentages vs. the quadratic model

The 12.3%/8.7%/6.2%/4.8% grade-3 "growth rates" reported after Table 2 are not clearly derived from the quadratic growth-curve parameters reported in Section 4.3 (π0, π1, π2); please specify the formula used, or move this to a supplementary calculation.

Response: We have added a clarifying note to Fig. 6. The percentage growth rates reported after Table 2 are period-to-period relative increments computed on the Grade 3 subgroup means as Delta_t = (Y-bar_(t+1) - Y-bar_t) / Y-bar_t times 100%. These descriptive percentages summarize wave-to-wave change on the observed metric and are complementary to, rather than derived from, the parametric estimates pi_1 and pi_2 of the quadratic growth curve reported in Section 4.3, which characterize the underlying velocity (linear slope) and deceleration (quadratic curvature) of the modelled trajectory.

MINOR Copyediting and machine-translation artifacts

Inconsistent capitalization of author names in-text ("sweller et al.," "nurullizam," "schatschneider"), inconsistent terminology ("quadratic" vs. "secondary" growth pattern used interchangeably), and several awkward constructions throughout suggest the manuscript has not had a final proofreading pass. A full language edit is needed before publication.

Response: We have carefully proofread the manuscript to correct inconsistent author-name capitalizations (Sweller, Nurullizam, Schatschneider, Cer, and others are now capitalized consistently throughout the Introduction). Terminology has been standardized: "quadratic" is now used consistently for the second-order growth term, and other machine-translation artifacts have been revised. Prior to formal publication, the entire manuscript will additionally be sent for a professional language edit by a native English editorial service, and the corresponding Language Editing Certificate will be provided to the editorial office.

MINOR IRB approval number absent

Even once the Ethics Statement contradiction (Sec. 2.1) is resolved, the manuscript text itself should state the approval/reference number, not just the committee name.

Response: We have provided more detailed ethical approval information in the sections '3.2 Research participants and sampling' and 'Ethics Declaration', including the ethical approval number and date.

MINOR Effect-size benchmarking

Multiple standardized path coefficients (~0.3–0.5) are described as "medium and above effect quantity"; please cite the specific benchmark used for interpreting HLM path coefficients (Cohen's conventions were developed for correlations/regression R2, and their direct application to multilevel path coefficients should be justified or softened).

Response: We have added an explicit citation for the effect-size benchmarks used to interpret the multilevel path coefficients. Following Preacher and Kelley (2011, reference [29]), the thresholds used are |beta| < 0.10 small, 0.10 <= |beta| < 0.30 medium, |beta| >= 0.30 large. These thresholds are more stringent than the classic Cohen (1988) ranges for zero-order correlations, reflecting the reduction in effect size typically expected when adjusting for the multilevel dependence structure. The clarification appears as a Note appended to Table 8, and effect-size language throughout Section 4 uses these adjusted benchmarks.

MINOR Keyword inconsistency

The keyword list differs between the submission metadata ("...Longitudinal study") and the in-manuscript abstract ("...Data science applications in education"); please reconcile.

Response: We have reconciled the two keyword lists by unifying them into a single six-keyword list: Classical Chinese poetry learning; Cognitive load theory; Writing ability development; Hierarchical linear modeling; Longitudinal study; Data science applications in education. The unified list now appears in the manuscript abstract and will be entered identically in the submission metadata.

5. Strengths Worth Preserving

● A genuinely large, well-powered nested sample (1,920 students, 48 classes, 12 schools) with four measurement waves — a scale that is uncommon in classical-poetry-education research and, if the data hold up under the checks above, would be a valuable contribution.

Response: We are grateful for the reviewer's acknowledgement of the scale and design of the study. In the revised manuscript we have preserved the full multistage stratified random sampling framework (Section 3.2) and the four-wave longitudinal design, and have made the raw and item-level data for all 1,920 students by 4 waves openly available in the project GitHub repository so that the reviewer's checks can be fully replicated (see revised Data Availability).

● Appropriate use of a three-level HLM framework for the genuinely nested data structure, with sensible centering strategy (CWC for Level-1, CGM for Level-2/3).

Response: We appreciate this acknowledgement. In the revised manuscript we have extended the framework to a four-level model as recommended in Comment 3.6 (measurement occasions, students, classes, schools; see Section 3.5), while preserving the centering strategy (group-mean centering for Level 1 predictors and grand-mean centering for Level 2 through Level 4 predictors). This ensures clean parameter interpretation and reduces multicollinearity across the four levels.

● The mediation and cross-level moderation analyses target a real and under-examined theoretical gap: how instructional context modulates the cognitive-load-to-writing pathway across developmental stages.

Response: We thank the reviewer for identifying the theoretical contribution of the mediation and cross-level moderation analyses. In the revised manuscript we have strengthened both by (a) fully operationalizing the three mediators (PSCT, IGVT, PERS; see Comment 3.1) and (b) demonstrating discriminant validity among the mediators, the poetry comprehension test, and the writing outcome (see Comment 3.2, new Section 4.2.3, and new Table 5). The cross-level moderation analysis retains its original interpretation while the underlying mediating pathways now have full psychometric documentation.

● Reported robustness checks (multiple imputation vs. complete case, bootstrap CIs, outlier-robust estimation) are a good practice and, once the modeling pipeline is clarified (Sec. 2.4), should be retained.

Response: We appreciate this acknowledgement, and the robustness checks are retained. As requested in Comment 2.4, we have also clarified the modeling pipeline: R version 4.3.2 with lme4 and lmerTest for REML estimation, brms with cmdstanr backend for Bayesian estimation of the moderated-mediation model, lavaan for CFA, and Python 3.11 with statsmodels and scikit-learn for the prediction pipeline. The BLUP-augmented mixed-model predictions are distinguished from the three-band classification derived post-hoc from the same regression predictions, and the modeling software and complete analysis scripts are archived in the GitHub repository.

6. Checklist of Requested Revisions

■ 1. Resolve the Ethics Statement contradiction; provide IRB name, approval number, and parental/guardian consent details for minor participants.

■ 2. Fully re-verify and correct every in-text citation number against the reference list (or vice versa).

■ 3. Confirm the GitHub data repository is public, stable, and contains the full minimal dataset (not only code); provide a permanent identifier (e.g., DOI via Zenodo/OSF) if possible.

■ 4. Clarify the exact algorithm(s) used to generate the "prediction model," reconcile the R2

/RMSE (regression) and accuracy/ROC-AUC (classification) results, and name the software/packages used throughout.

■ 5. Justify the mapping from NASA-TLX subscales to ICL/ECL/GCL, or relabel the construct as general perceived workload if a stronger justification isn't available.

■ 6. Add a brief, candid account of any non-significant or unexpected results encountered during model-building.

■ 7. Rewrite Table 8 and the surrounding discussion in instructional/curricular terms rather than ML-deployment terms.

■ 8. Fully describe the instruments (items, reliability, scoring) used to measure schema construction, imagery processing, and emotional resonance — currently undocumented despite anchoring the paper's central mediation result.

■ 9. Report discriminant validity (e.g., HTMT or inter-construct correlations) between the mediators, the poetry comprehension test, and the writing outcome to rule out item-content overlap as a driver of the mediation effect.

■ 10. Either add a cross-lagged/temporal-precedence analysis to support causal language, or revise

"predicted"/"effect" language throughout to association-level language.

■ 11. Address the plausibility of self-reported cognitive-load differentiation (ICL/ECL/GCL) in grade 3 children (~8 years old), or cite validation evidence for this age range.

■ 12. Reconcile equations (11)–(12) (ICC formulas) with the variance components actually reported in Table 5.

■ 13. Clarify whether the model is three-level or four-level given that Time/Time2 are embedded at Level 1, and state clearly what the reported 68.2% "student-level" variance does and does not include.

■ 14. Report VIF/tolerance statistics for ICL, ECL, and GCL given their moderate-to-strong pairwise correlations.

■ 15. Describe the writing-assessment rating procedure (number of raters per composition, blinding,

disagreement resolution).

■ 16. Reconcile the Discussion with the Reynaert et al. finding cited in the Introduction, and expand the Limitations section to cover measurement and design limitations, not only sample generalizability.

■ 17. Address the minor items in Section 4, and commission a full language/copyediting pass

Response: We again thank the reviewer for the detailed and constructive review, which has substantially strengthened the manuscript. All revisions have been highlighted in the revised manuscript for the reviewer's convenience, and the analysis code, data, and measurement instruments are archived in the project GitHub repository (see Data Availability) to support full reproducibility

Attachment Submitted filename: Response to Reviewers.docx

# Peer review: Decision Letter 1

**DOI:** 10.1371/journal.pone.0361497.r003

**Authors:** Dawit Dibekulu

22 Sep 2026

Dear Dr. Shi,

Thank you for submitting your manuscript to PLOS One. After careful consideration, we feel that it has merit but does not fully meet PLOS One’s publication criteria as it currently stands. Therefore, we invite you to submit a revised version of the manuscript that addresses the points raised during the review process.

Please submit your revised manuscript by Nov 21 2026 11:59PM. If you will need more time than this to complete your revisions, please reply to this message or contact the journal office at plosone@plos.org. When you're ready to submit your revision, log on to https://www.editorialmanager.com/pone/ and select the 'Submissions Needing Revision' folder to locate your manuscript file.

- A letter that responds to each point raised by the academic editor and reviewer(s). You should upload this letter as a separate file labeled 'Response to Reviewers'.
- A marked-up copy of your manuscript that highlights changes made to the original version. You should upload this as a separate file labeled 'Revised Manuscript with Track Changes'.
- An unmarked version of your revised paper without tracked changes. You should upload this as a separate file labeled 'Manuscript'.

If you would like to make changes to your financial disclosure, please include your updated statement in your cover letter. Guidelines for resubmitting your figure files are available below the reviewer comments at the end of this letter.

If applicable, we recommend that you deposit your laboratory protocols in protocols.io to enhance the reproducibility of your results. Protocols.io assigns your protocol its own identifier (DOI) so that it can be cited independently in the future. For instructions see: https://journals.plos.org/plosone/s/submission-guidelines#loc-laboratory-protocols. Additionally, PLOS One offers an option for publishing peer-reviewed Lab Protocol articles, which describe protocols hosted on protocols.io. Read more information on sharing protocols at https://plos.org/protocols?utm_medium=editorial-email&utm_source=authorletters&utm_campaign=protocols.

As the corresponding author, your ORCID iD is verified in the submission system and will appear in the published article. PLOS supports the use of ORCID, and we encourage all coauthors to register for an ORCID iD and use it as well. Please encourage your coauthors to verify their ORCID iD within the submission system before final acceptance, as unverified ORCID iDs will not appear in the published article. *Only*  the individual author can complete the verification step; PLOS staff *cannot*  verify ORCID iDs on behalf of authors.

We look forward to receiving your revised manuscript.

Kind regards,

Dawit Dibekulu, PhD

Academic Editor

PLOS One

Journal Requirements:

If the reviewer comments include a recommendation to cite specific previously published works, please review and evaluate these publications to determine whether they are relevant and should be cited. There is no requirement to cite these works unless the editor has indicated otherwise.

[Note: HTML markup is below. Please do not edit.]

Reviewers' comments:

Reviewer's Responses to Questions

**Comments to the Author**

Reviewer #2: (No Response)

Reviewer #3: (No Response)

**********

2. Is the manuscript technically sound, and do the data support the conclusions?

Reviewer #2: Partly

Reviewer #3: Partly

**********

3. Has the statistical analysis been performed appropriately and rigorously?

Reviewer #2: No

Reviewer #3: I Don't Know

**********

4. Have the authors made all data underlying the findings in their manuscript fully available?

The PLOS Data policy

Reviewer #2: Yes

Reviewer #3: Yes

**********

5. Is the manuscript presented in an intelligible fashion and written in standard English?

Reviewer #2: Yes

Reviewer #3: Yes

**********

Reviewer #2: The authors have made substantial efforts to address the comments raised in the previous round of review. The revised manuscript is considerably clearer in its methodological description, including the specification of the four-level longitudinal structure, operationalization and psychometric evaluation of the study measures, documentation of the writing assessment and inter-rater reliability procedures, clarification of the prediction outcomes, and additional sensitivity analyses. These revisions have strengthened the manuscript considerably. However, two methodological issues raised in my previous review remain insufficiently resolved and prevent me from considering the manuscript fully technically and statistically sound at this stage.

1. Cross-validation and the hierarchical structure of the data.

My previous review raised concern regarding whether the cross-validation procedure adequately reflects the hierarchical structure of the data. The revised manuscript clarifies that the prediction model is evaluated using “10-fold cross-validation stratified by student ID and grade” and that predictions incorporate BLUP random-effect terms from the four-level model. However, it remains unclear what level of out-of-sample generalization the reported performance is intended to represent. Student-level splitting can retain classes and schools across training and validation folds and therefore evaluates prediction for students drawn from already represented higher-level contexts rather than prediction to entirely unseen classes or schools. Please clarify the intended prediction target. If the reported performance is intended to support generalization beyond the sampled classes and schools, an additional cluster-aware validation analysis, such as leave-class-out and/or leave-school-out validation, would be more appropriate. The authors should also clarify how BLUPs were obtained within each validation fold and confirm that outcome information from validation observations was not used in estimating the corresponding random effects.

2. Number of Level-4 units and stability of school-level estimates.

The revised four-level specification resolves the previous ambiguity concerning the hierarchical structure by explicitly modeling measurement occasions within students, students within classes, and classes within schools. The manuscript also now reports several useful robustness analyses. Nevertheless, the highest level contains only 12 schools. The current robustness analyses address missing-data handling, outliers, centering, and alternative model specifications, but they do not directly establish the stability of Level-4 variance components and school-level or cross-level estimates with only 12 Level-4 units. The manuscript cites the relative robustness of Bayesian estimation with small higher-level cluster counts, but this does not by itself demonstrate stability in the present dataset. I therefore recommend an analysis or justification specifically addressing the sensitivity of the principal Level-4 and cross-level results to the limited number of schools.

Overall, many of my previous concerns have been satisfactorily addressed, and the manuscript has improved substantially. My remaining concerns are considerably narrower than in the previous round. However, because they bear directly on the interpretation of the predictive validation and the reliability of inference at the highest level of the hierarchical structure, I consider them substantive rather than merely editorial. Addressing these points would strengthen the statistical support for the manuscript's conclusions.

Reviewer #3: Despite an interesting topic， a multi-year longitudinal dataset and advanced statistical analyzes， some issues need clarification․ The mapping of NASA-TLX dimensions onto intrinsic， extraneous and germane cognitive load should be seen as preliminary and should be interpreted with caution․ Interpretative assumptions are strong․ Since all analyzes (RI-CLPM， mediation) are based on observational data， it is wise to avoid overstating possible directional and causal claims․ The authors should clarify whether the multiple imputations， preprocessing， fitting of the model， and estimation of the BLUP were done separately in each cross-validation fold， to avoid accidental data leaks․ Since there are many tests and predictions conducted， the authors should clarify which tests are confirmatory and which are exploratory， and not overextend their conclusions or recommendations beyond what their results support․

**********

what does this mean?). If published, this will include your full peer review and any attached files.

If you choose “no”, your identity will remain anonymous but your review may still be made public.

**Do you want your identity to be public for this peer review?**  For information about this choice, including consent withdrawal, please see our Privacy Policy

Reviewer #2: **Yes:**  Nada AlJamal

Reviewer #3: No

**********

[NOTE: If reviewer comments were submitted as an attachment file, they will be attached to this email and accessible via the submission site. Please log into your account, locate the manuscript record, and check for the action link "View Attachments". If this link does not appear, there are no attachment files.]

To ensure your figures meet our technical requirements, please review our figure guidelines: https://journals.plos.org/plosone/s/figures

You may also use PLOS’s free figure tool, NAAS, to help you prepare publication quality figures: https://journals.plos.org/plosone/s/figures#loc-tools-for-figure-preparation.

NAAS will assess whether your figures meet our technical requirements by comparing each figure against our figure specifications.

# Author comment: Author response to Decision Letter 2

**DOI:** 10.1371/journal.pone.0361497.r004

6 Oct 2026

Reviewer #2:The authors have made substantial efforts to address the comments raised in the previous round of review. The revised manuscript is considerably clearer in its methodological description, including the specification of the four-level longitudinal structure, operationalization and psychometric evaluation of the study measures, documentation of the writing assessment and inter-rater reliability procedures, clarification of the prediction outcomes, and additional sensitivity analyses. These revisions have strengthened the manuscript considerably. However, two methodological issues raised in my previous review remain insufficiently resolved and prevent me from considering the manuscript fully technically and statistically sound at this stage.

1. Cross-validation and the hierarchical structure of the data.

My previous review raised concern regarding whether the cross-validation procedure adequately reflects the hierarchical structure of the data. The revised manuscript clarifies that the prediction model is evaluated using “10-fold cross-validation stratified by student ID and grade” and that predictions incorporate BLUP random-effect terms from the four-level model. However, it remains unclear what level of out-of-sample generalization the reported performance is intended to represent. Student-level splitting can retain classes and schools across training and validation folds and therefore evaluates prediction for students drawn from already represented higher-level contexts rather than prediction to entirely unseen classes or schools. Please clarify the intended prediction target. If the reported performance is intended to support generalization beyond the sampled classes and schools, an additional cluster-aware validation analysis, such as leave-class-out and/or leave-school-out validation, would be more appropriate. The authors should also clarify how BLUPs were obtained within each validation fold and confirm that outcome information from validation observations was not used in estimating the corresponding random effects.

Response:

We thank the reviewer for this comment. Three prediction targets are now distinguished, each evaluated with its own cross-validation design, and the fold-wise computation of the BLUPs and the exclusion of validation outcomes from every random-effect prediction are stated explicitly.

(1) Intended prediction target and cluster-aware validation designs. Section 3.6 states that the predictive performance of a multilevel model depends on which units are unseen at the time of prediction, and that student-level splitting supports generalization only to new students drawn from higher-level contexts already represented in the training folds. Three prediction targets are therefore distinguished. Design A (student-grouped 10-fold cross-validation) evaluates new students within the sampled classes and schools and is the confirmatory predictive criterion of O6; Design B (leave-one-class-out cross-validation over all 48 classes) evaluates students in entirely unseen classes within the sampled schools; and Design C (leave-one-school-out cross-validation over all 12 schools, holding out the four classes and 160 students of a school together) evaluates students in entirely unseen schools. Designs B and C are the leave-class-out and leave-school-out validation analyses suggested in the comment, and they are reported without acceptance thresholds because O6 does not preset criteria for those two targets; Figure 6A summarizes the three designs and the random effects that enter each prediction. Table 8 (Section 4.5.1) reports R² = 0.847 (95% CI [0.834, 0.859]), RMSE = 4.28, and 80.3% three-band accuracy under Design A (the preset RMSE target is met, whereas the 85% accuracy target is not), R² = 0.768 and RMSE = 5.24 under Design B, and R² = 0.656 and RMSE = 6.38 under Design C. Fold-level, class-level, and school-level metrics for the three designs are provided in Supplemental Table S8.

(2) How BLUPs are obtained within each validation fold. Section 3.6 formalizes the BLUP computation within each fold and states, for each of the three designs, from which training-fold observations the class-level and school-level BLUPs are estimated. In Design A, the class and school BLUPs of a validation student are estimated jointly from the training-fold observations of that student’s classmates and of the other three classes of that school, and the four measurement waves of the validation student are excluded from both. In Design B, the class BLUP is unavailable because every observation in the held-out class is held out, and the school BLUP is estimated from the training-fold observations of the other three classes of that school. In Design C, every class and school random effect of the held-out school is set to zero because no training-fold observation from that school is available for either BLUP. Equations (19) to (21) give the prediction expression of each design, and Section 4.5.1 restates this rule before the predictive results are reported.

(3) Exclusion of validation outcome information from BLUP estimation. The BLUP expression b̂^(−f) = Ĝ Z′_(−f) V̂_(−f)^(−1) (Y_(−f) − X_(−f) γ̂^(−f)) in Section 3.6 makes explicit that the outcomes, fixed effects, and variance components that enter every BLUP are computed within each training fold from the training-fold outcomes, design matrices, and variance components alone, so outcome information from the validation fold enters neither the fixed effects, the variance components, nor any random-effect prediction. Algorithm 1 (Section 3.5, step 7) outlines the fold-wise pipeline, and Supplemental Table S7 enumerates its twelve data-dependent steps: multiple imputation with predictive mean matching (m = 20), Level-1 within-student centering, Level-2 to Level-4 grand-mean centering, growth-order and random-effects reselection, REML refitting, class-level and school-level BLUP estimation, grade-normed band-cutoff derivation, band-probability derivation, leave-one-covariate-out refits, and averaging over the 20 imputed datasets are all carried out on the training folds and applied unchanged to the validation folds, and validation observations without an observed outcome are excluded from the performance metrics. Supplemental Table S7 also records for every step whether validation outcomes were used: the column returns “No” for every estimation step and “Scoring only” for the three steps that score out-of-fold predictions (step 9, leave-one-covariate-out importance; step 11, performance metrics; step 12, the bootstrap interval of R²).

2. Number of Level-4 units and stability of school-level estimates.

The revised four-level specification resolves the previous ambiguity concerning the hierarchical structure by explicitly modeling measurement occasions within students, students within classes, and classes within schools. The manuscript also now reports several useful robustness analyses. Nevertheless, the highest level contains only 12 schools. The current robustness analyses address missing-data handling, outliers, centering, and alternative model specifications, but they do not directly establish the stability of Level-4 variance components and school-level or cross-level estimates with only 12 Level-4 units. The manuscript cites the relative robustness of Bayesian estimation with small higher-level cluster counts, but this does not by itself demonstrate stability in the present dataset. I therefore recommend an analysis or justification specifically addressing the sensitivity of the principal Level-4 and cross-level results to the limited number of schools.

Response:

We thank the reviewer for this important comment. The manuscript no longer relies on the general robustness of Bayesian estimation to small higher-level cluster counts as evidence of stability in the present data. Section 4.6.5 states that this general robustness is by itself insufficient to establish the stability of the Level-4 variance component and of the school-level and cross-level coefficients in the present dataset, and that each of these estimates was therefore examined with five dedicated analyses, which are specified in Section 3.5 and each target a distinct aspect of small-cluster inference:

(a) Twelve leave-one-school-out (LOSO) refits, each of which drops one school and re-estimates the four-level model on the remaining 11 schools, quantifying the influence of every individual school on each estimate; complete LOSO estimates are reported in Supplemental Table S10.

(b) A school-level wild cluster bootstrap using Webb weights (B = 9,999) for the coefficients, which adjusts the interval width for the small number of clusters without relying on the asymptotic Wald standard errors that are unreliable under these conditions, and a school-level case bootstrap for the school-level variance component and ICC_school.

(c) Bayesian re-estimation under three alternative priors on the random-effect standard deviations (half-Cauchy(0, 5), half-Student-t(3, 0, 10), and Uniform(0, 30)) in addition to the reference inverse-gamma prior, which characterizes the prior sensitivity of the Bayesian estimates; complete prior-sensitivity results are reported in Supplemental Table S11.

(d) A school fixed-effects specification that removes the between-school variance component entirely for the coefficients that vary within schools.

(e) A calibrated Monte Carlo simulation of 1,000 datasets generated from the fitted model with the observed 12-school design, which returns the empirical bias, sampling variability, and interval coverage that the inference procedures actually deliver under that design; complete simulation results are reported in Supplemental Table S12.

Table 11 (Section 4.6.5) collects the results of all five analyses for the six principal estimates: the school-level intercept variance τ_v000, ICC_school, Teaching × CogLoad, Grade × CogLoad, Environment × CogLoad, and the moderated-mediation index. Figure 12 displays the leave-one-school-out range of each estimate together with its Kenward-Roger, profile-likelihood, or Monte Carlo interval and its bootstrap interval, scaled to the main estimate. A coefficient is classified as Stable when its sign is unchanged in all 12 LOSO refits, its Kenward-Roger and wild-bootstrap 95% intervals exclude zero, its 95% credible interval excludes zero under all four priors, and the calibrated simulation returns nominal-95% coverage of at least 0.925, and a variance component or ICC whose intervals reach the boundary of the parameter space is classified as Imprecise (note to Table 11).

Applying these criteria separates the six estimates into three groups (Section 4.6.5). The Teaching × CogLoad interaction (γ = 0.47, LOSO range [0.39, 0.56], wild-bootstrap 95% CI [0.11, 0.83], simulated coverage 0.945) and the moderated-mediation index (0.07, LOSO range [0.04, 0.10], wild-bootstrap 95% CI [0.02, 0.12], simulated coverage 0.930) satisfy every criterion and remain interpretable under the 12-school design. The school-level intercept variance (τ_v000 = 10.03) and ICC_school (0.083) remain positive in all 12 LOSO refits (ranges [6.21, 14.88] and [0.053, 0.118]), and the boundary-corrected likelihood-ratio test supports a non-zero school variance (χ̄² = 3.53, p = .030); however, their 95% profile-likelihood intervals ([0.00, 38.69] and [0.000, 0.260]) and school-level bootstrap intervals ([0.00, 20.28] and [0.000, 0.160]) reach the boundary of the parameter space, the simulation shows that the school-level case bootstrap undercovers (0.818) whereas the profile-likelihood interval is close to nominal (0.959), and the likelihood-ratio test detects a school variance of this size in only 57% of the simulated datasets (Supplemental Table S12). Both are therefore classified as Imprecise: the school level is retained because it reflects the sampling design and protects the standard errors of the class-level and cross-level coefficients, and the magnitude of school-level clustering is not interpreted (Section 4.3.1; Section 5.1; Section 5.3). Table 6 (Section 4.3.1) reports the asymptotic SEs of the variance components for reference only, together with boundary-corrected likelihood-ratio statistics and 95% profile-likelihood and parametric bootstrap intervals, in place of the Wald z statistics, and Section 4.6.3 reports that the four-level specification is retained over a three-level alternative by the boundary-corrected likelihood-ratio test (χ̄² = 7.9, p = .034) and on design grounds, although AIC barely separates the two models and BIC favours the three-level model. The Grade × CogLoad (γ = 0.39) and Environment × CogLoad (γ = 0.31) interactions fail the wild-bootstrap and coverage criteria and are prior-sensitive; they are therefore marked Exploratory in Table 9 and are not used as support for instructional recommendations at Level 4. Complete results are provided in Supplemental Tables S10 (leave-one-school-out refits), S11 (prior sensitivity), and S12 (simulation), and the analytic registry in Supplemental Table S9 records the pre-specified stability criteria and the resulting classification (Stable, Exploratory, or Imprecise) of every Level-4 and cross-level estimate. The Limitations (Section 5.3) acknowledge that the highest level comprises only 12 schools, which limits precision at Level 4 and for cross-level estimates, and the Conclusion (Section 6) lists designs with more schools as the natural next step for sharpening Level-4 inference.

Overall, many of my previous concerns have been satisfactorily addressed, and the manuscript has improved substantially. My remaining concerns are considerably narrower than in the previous round. However, because they bear directly on the interpretation of the predictive validation and the reliability of inference at the highest level of the hierarchical structure, I consider them substantive rather than merely editorial. Addressing these points would strengthen the statistical support for the manuscript's conclusions.

Reviewer #3:Despite an interesting topic‚ a multi-year longitudinal dataset and advanced statistical analyzes‚ some issues need clarification․ The mapping of NASA-TLX dimensions onto intrinsic‚ extraneous and germane cognitive load should be seen as preliminary and should be interpreted with caution․ Interpretative assumptions are strong․ Since all analyzes (RI-CLPM‚ mediation) are based on observational data‚ it is wise to avoid overstating possible directional and causal claims․ The authors should clarify whether the multiple imputations‚ preprocessing‚ fitting of the model‚ and estimation of the BLUP were done separately in each cross-validation fold‚ to avoid accidental data leaks․ Since there are many tests and predictions conducted‚ the authors should clarify which tests are confirmatory and which are exploratory‚ and not overextend their conclusions or recommendations beyond what their results support․

Response:

We thank the reviewer for the careful reading. The four points are addressed explicitly throughout the manuscript, and interpretive statements are kept within the limits that the observational design and the provisional TLX mapping support.

(1) NASA-TLX to ICL/ECL/GCL mapping. Section 3.3 presents the mapping as provisional under three explicit assumptions, notes that the ECL indicator is the least direct link because perceived performance also reflects self-efficacy and task success, states that germane load is now regarded as the working-memory resources devoted to intrinsic load rather than as an independent additive load, and cautions that the labels ICL, ECL, and GCL denote TLX-indexed indicators throughout the article, so results that depend on their distinction are interpreted as preliminary. Section 4.6.4 reports four dedicated measurement analyses: competing measurement models of the TLX subscales at every wave, configural, metric, and scalar invariance across Grades 3 to 6, a multitrait-multimethod comparison with the Klepsch et al. instrument in a stratified 20% subsample (n = 384) at T3, and a sensitivity model with a single undifferentiated load score. The three-factor model fits best at every wave, invariance holds across grades, every monotrait-heteromethod correlation exceeds its heterotrait-monomethod counterparts, and Klepsch-based re-estimation preserves the signs of the three load effects, each within 0.05 of its NASA-TLX counterpart (ICL β = −0.37, ECL β = −0.47, GCL β = 0.48 versus −0.41, −0.49, 0.45), with a mediation index of 0.56 (95% CI [0.47, 0.65]) indistinguishable from 0.587. The undifferentiated load score removes the teaching-method interaction (γ = 0.09, p = .53), so the O5 finding depends on the load-type distinction and is interpreted with the same caution. Supplemental Table S6 provides the full results. The Discussion (Section 5.1) and the Limitations (Section 5.3) restrict any interpretation of the load-component differentiation to the provisional-mapping caveat, and Table 13 embeds the caveat in the O1 implication row.

(2) Directional and causal claims. All analyses based on observational data are stated as statistical associations rather than causal effects. Section 2.5 states that because the predictors, mediators, and outcome are observed rather than manipulated, the indirect effects are interpreted as statistical indirect associations under the hypothesized ordering rather than as demonstrated causal mechanisms. The RI-CLPM analysis (Section 4.3.5) reports within-person lagged associations rather than causal effects, and the mediation analysis (Section 4.4.1) reports statistical indirect associations under the hypothesized ordering rather than demonstrated causal mediation. Section 4.6.6 further examines the assumed ordering with three analyses: re-estimating the RI-CLPM with grade, gender, school location, and teaching method as time-invariant covariates and poetry comprehension as a time-varying covariate (Supplemental Table S13, Panel A); a temporally ordered mediation model from cognitive load at one wave through each mediator at the next wave to writing ability at the wave after (T1 to T3 and T2 to T4, Panel C); and the residual mediator-outcome correlation that would eliminate each temporally ordered indirect association. The three thresholds (0.21, 0.18, and 0.14) exceed the largest residual correlation produced by any measured covariate (0.09 for teaching method, Panel D), so an unmeasured confounder would need a stronger residual association than any measured covariate to remove an indirect association. Section 5.3 lists the observational design and the impossibility of ruling out unmeasured confounders such as shifts in reading habits or parental involvement as an explicit limitation, and the Conclusion (Section 6) restricts instructional recommendations to the confirmatory findings that meet the preset acceptance criteria under Holm familywise error control.

(3) Multiple imputation, preprocessing, model fitting, and BLUP estimation within each cross-validation fold. Section 3.6 states that all data-dependent steps are carried out within folds; Algorithm 1 (Section 3.5, step 7) outlines the fold-wise pipeline, and Supplemental Table S7 enumerates its twelve steps. Multiple imputation with predictive mean matching (m = 20) is estimated on the training folds only, validation outcomes are neither used in the imputation models nor imputed, and validation observations without an observed outcome are excluded from the performance metrics. Level-1 predictors are centered on each student’s own mean, and Level-2 to Level-4 predictors on training-fold grand means. The growth order and random-effects structure are reselected in each training fold by AIC/BIC, and the four-level model is re-estimated by REML in the training fold. BLUP estimation is confined to the training-fold outcomes, design matrices, and variance components, as detailed in Section 3.6 (lines 404-430) and in item (3) of the response to Reviewer #2, Comment 1 above. Band cutoffs, band probabilities for ROC-AUC, and leave-one-covariate-out refits are also confined to the training folds, and predictions are averaged over the 20 imputed datasets within each fold. Supplemental Table S7 documents the validation-outcome status of every step and returns “No” for every estimation step and “Scoring only” for the three steps that score out-of-fold predictions (steps 9, 11, and 12).

(4) Confirmatory versus exploratory tests. Section 2.5 designates four hypotheses as confirmatory: the three TLX-indexed load-component associations of O1, the three indirect effects of O3, the interaction of cognitive load with teaching method of O5, and the quadratic growth term of O6. These eight tests form one family whose p values are adjusted with the Holm procedure, and predictive performance for new students within the sampled classes and schools (Design A) serves as the confirmatory predictive criterion of O6. All remaining analyses are exploratory under Benjamini-Hochberg control within their own family and generate hypotheses rather than support conclusions or instructional recommendations. Table 9 (Section 4.5.2) attaches an explicit inferential tier (Confirmatory (Holm) or Exploratory (BH)) and both the raw and multiplicity-adjusted p values to every path. Table 9 records that the eight confirmatory tests retain significance under Holm control, that O6 is partially supported (RMSE 4.28 meets the 5.0-point threshold, whereas three-band accuracy of 80.3% falls short of the 85% target, so predicted bands are recommended for monitoring rather than placement decisions), and that the O2 and O4 paths, the Grade × CogLoad and Environment × CogLoad interactions, and the non-significant analyses of Section 4.5.3 are exploratory. The Discussion (Section 5.1) and Table 13 restrict the instructional and curricular implications to the confirmatory findings, and the Grade 4 ECL peak, the exploratory Grade × CogLoad and Environment × CogLoad interactions, and the exploratory O2 and O4 results are explicitly reported as hypothesis-generating extensions to be re-examined before informing instructional recommendations. Supplemental Table S9 provides the analytic registry that records the pre-specification, family membership, tier, and adjusted p value of every inferential test.

Attachment Submitted filename: Response to Reviewers.doc

# Editor report: Decision Letter 2

**DOI:** 10.1371/journal.pone.0361497.r005

**Authors:** Dawit Dibekulu

12 Oct 2026

Construction of a Hierarchical Linear Regression Prediction Model for Classical Chinese Poetry Writing Ability Development

PONE-D-26-25548R2

Dear Dr. Shi,

We’re pleased to inform you that your manuscript has been judged scientifically suitable for publication and will be formally accepted for publication once it meets all outstanding technical requirements.

Within one week, you’ll receive an e-mail detailing the required amendments. When these have been addressed, you’ll receive a formal acceptance letter and your manuscript will be scheduled for publication.

An invoice will be generated when your article is formally accepted. Please note, if your institution has a publishing partnership with PLOS and your article meets the relevant criteria, all or part of your publication costs will be covered. Please make sure your user information is up-to-date by logging into Editorial Manager at Editorial Manager® and clicking the ‘Update My Information' link at the top of the page. For questions related to billing, please contact billing support.

If your institution or institutions have a press office, please notify them about your upcoming paper to help maximize its impact. If they’ll be preparing press materials, please inform our press team as soon as possible -- no later than 48 hours after receiving the formal acceptance. Your manuscript will remain under strict press embargo until 2 pm Eastern Time on the date of publication. For more information, please contact onepress@plos.org.

Kind regards,

Dawit Dibekulu, PhD

Academic Editor

PLOS One

Additional Editor Comments (optional):

Reviewers' comments:

# Editor report: Acceptance letter

**DOI:** 10.1371/journal.pone.0361497.r006

**Authors:** Dawit Dibekulu

PONE-D-26-25548R2

PLOS One

Dear Dr. Shi,

I'm pleased to inform you that your manuscript has been deemed suitable for publication in PLOS One. Congratulations! Your manuscript is now being handed over to our production team.

At this stage, our production department will prepare your paper for publication. This includes ensuring the following:

* All references, tables, and figures are properly cited

* All relevant supporting information is included in the manuscript submission,

* There are no issues that prevent the paper from being properly typeset

You will receive further instructions from the production team, including instructions on how to review your proof when it is ready. Please keep in mind that we are working through a large volume of accepted articles, so please give us a few days to review your paper and let you know the next and final steps.

Lastly, if your institution or institutions have a press office, please let them know about your upcoming paper now to help maximize its impact. If they'll be preparing press materials, please inform our press team within the next 48 hours. Your manuscript will remain under strict press embargo until 2 pm Eastern Time on the date of publication. For more information, please contact onepress@plos.org.

You will receive an invoice from PLOS for your publication fee after your manuscript has reached the completed accept phase. If you receive an email requesting payment before acceptance or for any other service, this may be a phishing scheme. Learn how to identify phishing emails and protect your accounts at https://explore.plos.org/phishing.

If we can help with anything else, please email us at customercare@plos.org.

Thank you for submitting your work to PLOS One and supporting open access.

Kind regards,

PLOS One Editorial Office Staff

on behalf of

Dr. Dawit Dibekulu

Academic Editor

PLOS One
